# Leveraging mathematical models of disease dynamics and machine learning to improve development of novel malaria interventions

**DOI:** 10.1186/s40249-022-00981-1

**Published:** 2022-06-04

**Authors:** Monica Golumbeanu, Guo-Jing Yang, Flavia Camponovo, Erin M. Stuckey, Nicholas Hamon, Mathias Mondy, Sarah Rees, Nakul Chitnis, Ewan Cameron, Melissa A. Penny

**Affiliations:** 1 Swiss Tropical and Public Health Institute, Allschwil, Switzerland; 2 Key Laboratory of Tropical Translational Medicine of Ministry of Education and School of Tropical Medicine and Laboratory Medicine, The First and Second Affiliated Hospital of Hainan Medical University, Hainan Medical University, Haikou, Hainan People’s Republic of China; 3 University of Basel, Basel, Switzerland; 4 Center for Communicable Disease Dynamics, Department of Epidemiology, Harvard T. H. Chan School of Public Health, Boston, MA 02115 USA; 5 The Bill & Melinda Gates Foundation, Seattle, WA USA; 6 Innovative Vector Control Consortium, Liverpool, UK; 7 Malaria Atlas Project, Big Data Institute, University of Oxford, Oxford, UK; 8 Curtin University, Perth, Australia; 9 Telethon Kids Institute, Perth Children’s Hospital, Perth, Australia

**Keywords:** Infectious diseases, Malaria, Novel interventions, Mathematical modelling, Machine learning

## Abstract

**Background:**

Substantial research is underway to develop next-generation interventions that address current malaria control challenges. As there is limited testing in their early development, it is difficult to predefine intervention properties such as efficacy that achieve target health goals, and therefore challenging to prioritize selection of novel candidate interventions. Here, we present a quantitative approach to guide intervention development using mathematical models of malaria dynamics coupled with machine learning. Our analysis identifies requirements of efficacy, coverage, and duration of effect for five novel malaria interventions to achieve targeted reductions in malaria prevalence.

**Methods:**

A mathematical model of malaria transmission dynamics is used to simulate deployment and predict potential impact of new malaria interventions by considering operational, health-system, population, and disease characteristics. Our method relies on consultation with product development stakeholders to define the putative space of novel intervention specifications. We couple the disease model with machine learning to search this multi-dimensional space and efficiently identify optimal intervention properties that achieve specified health goals.

**Results:**

We apply our approach to five malaria interventions under development. Aiming for malaria prevalence reduction, we identify and quantify key determinants of intervention impact along with their minimal properties required to achieve the desired health goals. While coverage is generally identified as the largest driver of impact, higher efficacy, longer protection duration or multiple deployments per year are needed to increase prevalence reduction. We show that interventions on multiple parasite or vector targets, as well as combinations the new interventions with drug treatment, lead to significant burden reductions and lower efficacy or duration requirements.

**Conclusions:**

Our approach uses disease dynamic models and machine learning to support decision-making and resource investment, facilitating development of new malaria interventions. By evaluating the intervention capabilities in relation to the targeted health goal, our analysis allows prioritization of interventions and of their specifications from an early stage in development, and subsequent investments to be channeled cost-effectively towards impact maximization. This study highlights the role of mathematical models to support intervention development. Although we focus on five malaria interventions, the analysis is generalizable to other new malaria interventions.

**Graphical abstract:**

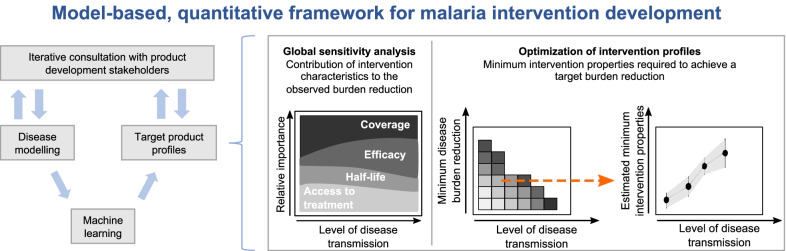

**Supplementary Information:**

The online version contains supplementary material available at 10.1186/s40249-022-00981-1.

## Background

Significant efforts to deploy malaria interventions worldwide have led to considerable progress and have reduced global malaria prevalence in Africa by half over the 2000 to 2015 period [1]. Reductions were achieved through a diverse range of interventions including mass distribution of insecticide-treated mosquito nets, indoor residual spraying, rapid diagnosis, as well as artemisinin-based combination therapies. However, since 2015 progress has stalled, and several countries have seen an increase in malaria incidence of over 40% [2]. Current interventions and malaria control programs are facing major challenges due to lack of funding, increases in drug and insecticide resistance and diagnostic-resistant parasites, as well as supply chain and deployment difficulties [3, 4]. Strategic investment and timely development of novel interventions is crucial to maintain the progress made and to advance towards malaria elimination [2, 5]. Two recent reports issued by the World Health Organization (WHO) [6] and the *Lancet Commission* [7] emphasize the need for novel malaria products, calling for a sustained investment in research and development (R&D).

Consequently, the malaria product development space has steadily expanded over the last 15 years. Novel products are diverse, ranging from therapeutic and immunological interventions, such as drugs and vaccines, to new vector control tools. Here we provide an overview of these novel interventions and explore the properties for a subset of key interventions.

With over 13 new drug compounds in early clinical development [2, 8], new antimalarial therapies will hopefully be available in the next 5 years [9]. Nevertheless, emergence of drug resistance remains a threat for novel drugs, advocating for products that ensure sustained protection. Several malaria vaccine candidates are under development [10, 11], and after 30 years including phase 3 clinical trial and pilot implementation [12–14] the RTS,S/AS01 vaccine has potential to avert mortality in children in combination with other interventions [15–17], including as seasonal prevention [18]. Several other vaccines are in phase 2 clinical studies, such as R21 which demonstrated a clinical efficacy of up to 77% [19]. More recently, biological alternatives to vaccines include passive immunization with injectable small molecules [20] or monoclonal antibodies [21–23]. Conferring protection against malaria during several months and being safe to administer during pregnancy, monoclonal antibodies are seen as potential interventions for seasonal malaria chemoprevention and protection for certain risk groups, with first human trials of monoclonal antibodies ongoing [7, 23].

Vector control has seen active development over the past years, with over 10 different categories of novel products aiming to reduce both indoor and outdoor mosquito biting [24–26]. These include new insecticides [27], vector traps [28, 29], as well as genetically-altered mosquitoes that will eradicate mosquito populations (called ‘gene drives’) [30, 31]. Furthermore, improved housing has been shown to significantly reduce indoor mosquito biting [32, 33], and effective house traps or lures are being developed to supplement traditional indoor interventions [34].

Malaria products under development are defined through Target Product Profiles (TPPs), which constitute a vital reference for dialogue between various stakeholders (listed in the “Methods” section) to guide R&D investments. TPPs are dynamic documents used during the development of a cutting-edge medical product, defining its required characteristics to fulfill an unmet health need [35]. Given the large amount of malaria interventions currently in the development pipeline, a systematic approach is essential to inform development decisions and prioritization of novel interventions to ensure a sustainable investment of resources and a fast pace of innovation. Currently, there is no approach systematically incorporating quantitative evidence and the aforementioned operational aspects in malaria product development (including intervention deployment, efficacy, duration, decay, and public health impact) from early development stages.

**Fig. 1:**
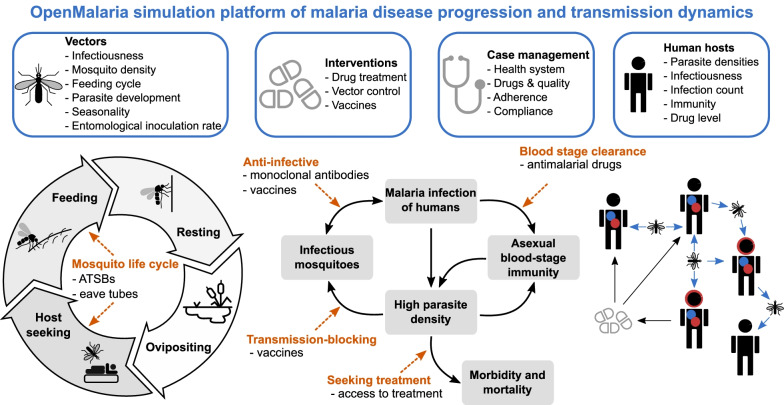
Simulation of malaria transmission dynamics with OpenMalaria: model schematic. Key components specified in the model are displayed on the top row including vector, intervention, case management, and human host-specific factors. Below, a detailed representation of the intrinsic modelled mosquito feeding and host transmission cycles is provided. The dashed, orange arrows mark the action and targets of the modelled interventions in this study, as indicated below the arrows. ATSBs stands for attractive targeted sugar baits. A detailed overview of model assumptions and parameters is provided in the “Methods” section and Additional file 1: Sect. 1 and Tables S1.1‒S1.3

Mathematical models of malaria transmission dynamics can be used to bridge this gap, as they quantitatively estimate the impact of interventions while including considerable evidence of disease progression and transmission, host immunity, as well as environmental or health system dynamics and their interaction with interventions [36] (Fig. 1). These models have been used extensively to estimate the impact of malaria interventions and to optimize intervention packages for specific geographies [37, 38]. Here an established individual-based malaria-transmission model, OpenMalaria [37, 39–43], has been used to simulate epidemiological disease and intervention dynamics to project the impact on public health, as has been employed to conduct several consensus modeling and validation studies [37, 41–43].

**Fig. 2:**
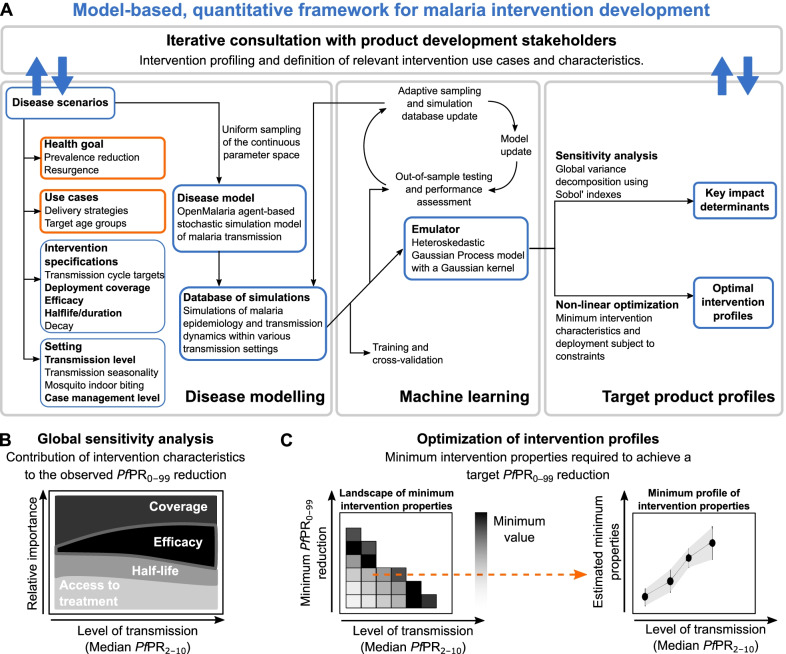
Overview of the approach to quantitatively define TPPs for novel malaria interventions. **A** Schematic description of the proposed model-based, quantitative framework to guide malaria product development. Results for applying this framework to guide development of five novel malaria interventions are provided for a range of simulated, true median *Pf*PR_2–10_ (before intervention deployment), and schematically described in subsequent figure panels. **B** Global sensitivity analysis for identifying the determinants of intervention impact: colors define intervention specifications, deployment coverage, and health system access levels varied in the analysis; the magnitude of the colored area at different levels of transmission (x-axis) represents the relative importance (y-axis) attributable to factors driving the observed *Pf*PR_0–99_ reductions following intervention deployment. **C** Optimization of intervention properties to achieve desired health goals: the heatmap (left panel) displays, for a given intervention property (coverage, efficacy, or half-life), the landscape of minimum required values to achieve various target *Pf*PR_0–99_ reductions. Each row of the heatmap corresponds to a target of *Pf*PR_0–99_ reduction and constitutes a minimum profile of the considered intervention characteristic (right panel)

Due to absence of data at early intervention development stages and computational limitations in exploring a highly combinatorial parameter space of presumed intervention characteristics, models have mainly been used at late stages of intervention development. To date, models have only been minimally applied in directing the design of new interventions, or in understanding how intervention-specific, epidemiological, and systems factors jointly contribute to impact. Model investigations are usually informed by scenario analysis accounting for delivery and target age groups, as well as with properties of the new intervention pre-defined or informed by late clinical trials [41, 44, 45]. In these constrained scenarios, the detailed disease and intervention dynamics captured by the model tends to obscure the complex relationships between intervention parameters, operational factors, health outcomes, and public health impact [46]. Exhaustive scenario analyses are computationally expensive, rendering the full exploration of all possible interventions, in conjunction with all possible delivery scenarios, combinatorically infeasible. Previous approaches using disease models to inform TPPs have tackled the combinatorically complex parameter space by only exploring a discrete, constrained set of parameters [47–49]. These approaches have provided insightful knowledge and have emphasized the importance of using disease models for defining TPPs. Nevertheless, they have provided a constrained view of intervention specifications. Here, we propose a different approach (Fig. 2), where epidemiological models guide development of novel disease interventions designed to achieve quantified health goals from the early stages, placing the end goal of public health impact at the center of decision-making.

We covered a diverse spectrum of interventions in the malaria development space, pertaining to (1) anti-infective monoclonal antibodies, (2) anti-infective vaccines, (3) transmission-blocking vaccines, (4) outdoor attractive targeted sugar baits, and (5) eave tubes. We used our approach to understand the link between intervention characteristics and resulting impact, and to define the requirements of these interventions in terms of coverage, efficacy, and impact duration to reach desired prevalence reduction goals, contingent on operational constraints (Fig. 2). We show how modelling can support the development process, and introduce a framework that quantitatively defines malaria product characteristics within TPPs. Our approach illustrates how modelling enables translation of R&D efforts into potential impact.

## Methods

The approach introduced here combines infectious disease modeling with machine learning to understand determinants and define quantitative properties of target product profiles of new malaria interventions. In summary, we undertook an iteratively engaged exchange with malaria product development stakeholders to define desired outcomes and likely delivery use-cases of new malaria interventions. We then used mathematical models combined with machine learning to perform a directed search of the entire space of intervention profiles, to define properties of new interventions towards achieving the desired health goals. We used Gaussian processes (GPs) [50] to generate computationally light emulators that work in combination with the established OpenMalaria model of malaria dynamics. These emulators accurately captured the non-linear relationships between properties of deployed interventions, operational factors, and resulting health outcomes. They allowed efficient sensitivity analyses of intervention and health system parameters on predicted public health impacts at low computational cost. Furthermore, by coupling emulators with nonlinear optimization techniques, we constructed a predictive framework that identified key determinants of intervention impact as well as the minimal intervention profiles required for achieving a given health goal. The methodological approach is schematically outlined in Fig. 2 with a description of its components provided in the following sub-sections and complemented in Additional file 1.

### Stakeholder engagement and expert group discussions

There was active engagement and regular exchanges with different expert groups during development of the methodological framework for guiding TPPs of novel malaria interventions. Stakeholders involved in these discussions were the Bill and Melinda Gates Foundation (BMGF), the Innovative Vector Control Consortium (IVCC), the Program for Appropriate Technology in Health—Malaria Vaccine Initiative (PATH-MVI), and the World Health Organization (WHO).

Guided by the BMGF, the stakeholder engagement process included initial meetings, interim meetings to define analyses, and presentations of results. The aims of these exchanges were (1) to create a communication environment with stakeholders to be able to incorporate their feedback to ensure realistic representation of novel malaria interventions and of the simulated disease transmission settings in this approach (2), to continuously shape this methodology to enable generating the relevant quantitative evidence to inform TPPs of novel malaria interventions from early stages of product development, and (3) to define and refine the priority research questions to be addressed with our analysis.

The exchanges with the stakeholders were coordinated and guided by BMGF through regular meetings as follows. First, there was an initial convening with all stakeholders to frame the key study questions, to establish a network for iterative dialogue and to inform the partners about OpenMalaria, its components, features, and validity of assumptions. At these meetings, there were over 15 participants from BMGF, IVCC, PATH-MVI, and Swiss TPH. The health goal of malaria prevalence reduction in all ages was chosen during this meeting, as well as the five malaria interventions on which to focus our analysis. Second, several one-to-one iterative meetings were held with each stakeholder to define the way the five chosen interventions are implemented in OpenMalaria and to define relevant aspects to consider for their deployment (e.g., targets in the malaria transmission cycle, shape of decay, mass deployment, assessing impact at early and late follow-up), as well as putative ranges of their characteristics. We consulted with BMGF and PATH-MVI to refine the modeled vaccines, monoclonal antibodies, and drugs, and with IVCC to refine attractive targeted sugar baits and eave tubes. The set of simulated transmission settings (seasonality, indoor biting) were also defined during these meetings. Interim meetings were held with several stakeholders where we presented proof-of-concept and intermediate analyses. These meetings ensured building trust and a high level of confidence from our stakeholders in this analysis, while shaping the research questions. Additional context describing the iterative expert group discussions are provided in Additional file 1: Sect. 1.1

### Establishment of the open-source model

We used OpenMalaria v38.0 [39, 40], an established open-source stochastic, individual-based model to simulate malaria epidemiology and transmission dynamics across humans and mosquitoes in various settings with an overview in Fig. 1 and fully described in Additional file 1: Sect. 1.2 and Tables S1.1‒S1.3, with source code available from https://github.com/SwissTPH/openmalaria/. The OpenMalaria model was calibrated and validated in previous studies using historical epidemiological data [39, 40, 51], and this calibration was used for this study (as fully described in Additional file 1: Sect. 1.2.1‒1.2.2, including model components, core parameters and mosquito cycle dynamics in Additional file 1: Tables S1.1‒S1.3).

### Description of simulation experiments

The simulated human population size in this analysis was 10,000 individuals, with its age structure informed by health and demographic surveillance data for Ifakara, Tanzania [52]. It is assumed that no infections were imported over the entire study period. Health system characteristics, mosquito entomological parameters driving infection patters, and seasonal exposure patterns are described in Additional file 1: Sects. 1.2.1‒1.2.4, Figs. S2.1‒S2.3, and Tables S1.1‒S1.3. Parasite infections in simulated hosts are simulated individually and disease effects such as immunity, infectiousness to mosquitoes, morbidity, or mortality are tracked. Setting-specific characteristics include demographics, mosquito species entomological profiles are explicitly modelled and a wide range of human and vector interventions can be applied. Various health outcomes are monitored over time, including *Plasmodium falciparum* prevalence of infections (*Pf*PR), uncomplicated clinical or severe disease, hospitalization, and malaria mortality. Model assumptions have been described and validated with field data in previous studies [39, 53].

### Definition of intervention profiles, their impact and health goals

We built a standardized representation for each malaria intervention and modelled each intervention by identifying their action on parasite or vector targets during the malaria transmission cycle (orange arrows in Fig. 1). Accordingly, each intervention was defined by its target, the ranges of the deployment coverage, initial efficacy, half-life, or duration of effect as well as the type of efficacy decay (see Additional file 1: Sect. 1, Fig. S2.2 and Table 1 for detailed intervention specifications). For simplification, the words ‘half-life’ and ‘duration’ are used interchangeably to describe the longevity of an intervention effect. The simulated malaria transmission settings were defined by the yearly entomological inoculation rate (EIR), seasonality level, access to treatment, as well as proportion of indoor mosquitoes. Descriptions of quantification of the efficacy of a given therapeutic or immunologic intervention, as well as definitions of intervention targets (as shown in Fig. 1) are provided in Additional file 1: Sect. 1.2.3. Each intervention or combination of interventions was applied as mass intervention targeting to all ages equally, along with continuous case management. Additional file 1 also provides descriptions of deployment of mass intervention packages (Additional file 1: Sect. 1.2.3), how the input the EIR was translated to *Pf*PR_2–10_ (Additional file 1: Sect. 1.2.4), as well as definitions of intervention impact and health goals (Additional file 1: Sect. 1.2.5).

**Table 1:** Description and ranges of OpenMalaria simulation interventions and transmission settings

	Intervention	Coverage	Initial efficacy	Half-life or duration (years)	Decay type
	*Preventing infection*				
Simulated malaria intervention properties	Anti-infective vaccine	0–1	0.3–0.95	0.5–5	Weibull (k = 0.8) (Sigmoidal)
	Anti-infective monoclonal antibody	0–1	0.3–0.95	0.167–0.667	Weibull (k = 3) (Biphasic)
	*Blood stage clearance*				
	Antimalarial drugs	0–1	0.8–1	0–0.1667	Exponential
	*Transmission blocking*				
	Vaccine	0–1	0.3–0.95	0.5–5	Weibull (k = 0.8) (Biphasic)
	*Preprandial killing effect (affects indoor mosquito biting)*				
	Eave tubes	0–1	0.3–0.99	0.5–5	Weibull (k = 3) (Sigmoidal)
	*Preprandial and postprandial killing effect (affects outdoor mosquito biting)*				
	Attractive targeted sugar baits	0–1	0.7–0.99	0.167–0.667	Step
Simulated malaria transmission settings	*EIR range*: 1–25, representing a *Pf*PR_0–99_ of 13–88% and a *Pf*PR_2–10_ of 7.2–74.0%				
	*Case management (baseline scenario) range:* 0–0.8, corresponding to a probability range of seeking care within 5 days from the onset of fever of 0–0.5 *Seasonality levels* 1. High seasonal setting with one transmission peak over a year 2. Perennial setting with constant yearly transmission				
	*Proportion of indoor-biting mosquitoes, out of total indoor and outdoor biting mosquitoes:* 3. High (0.8) 4. Medium (0.5) 5. Low (0.2)				

Interventions and transmission settings were defined through consultation with product development stakeholders. Parameters varied within the OpenMalaria simulations include characteristics of applied malaria interventions (see Additional file 1: Fig. S2.2 for visual ranges of these parameters), as well as malaria transmission setting characteristics.

EIR entomological inoculation rate, *Pf*PR_0–99_ prevalence of *Plasmodium falciparum* malaria in all ages, *Pf*PR_2–10_ prevalence of *Plasmodium falciparum* malaria in 2-to-10-year-olds

### Disease model emulator with Gaussian processes

As it was computationally intensive to simulate an exhaustive number of simulations to explore the entire parameter space for diverse combinations of interventions, settings, and deployments, machine learning techniques and kernel methods were applied. A full description of how the model emulator was built with Gaussian processes is provided in Additional file 1: Sect. 2.1, along with a description of how the training dataset was built for each intervention and setting (Additional file 1: Sect. 2.2) and how emulators were trained on this dataset (Additional file 1: Sect. 2.3).

### Identifying impact determinants through sensitivity analysis

To estimate the contribution of each model input and its interactions with the other inputs to the variance of the model outcome, a global sensitivity analysis based on variance decomposition [54] was conducted. A complete description of the sensitivity analysis process can be found in Additional file 1: Sect. 4.1.

### Finding minimal intervention properties

The trained GP models for each transmission setting and intervention were used within a general-purpose optimization scheme to identify minimum intervention properties that reach a defined *Pf*PR_0–99_ reduction goal given operational and intervention constraints. The calculations for finding the minimal intervention properties are provided in Additional file 1: Sect. 5.1.

## Results

### Establishment of the approach to quantitatively define the framework guiding malaria product development

**Fig. 3:**
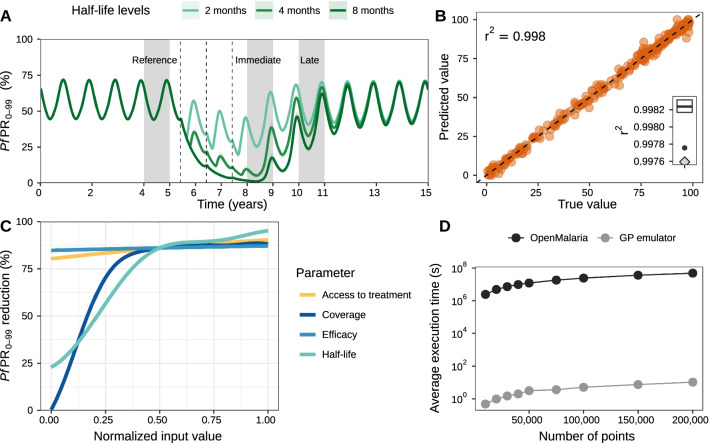
Training predictive Gaussian process emulators of simulated intervention impact with OpenMalaria. Examples are shown for attractive targeted sugar baits (ATSBs); results for other interventions are shown in Additional file 1: Figs. S3.1 and S4.2‒S4.7 and Table S4.1. **A** Simulated malaria *Pf*PR_0–99_ time series at EIR = 10 where ATSBs were deployed at a coverage of 70% and had an efficacy of 70%. Results are shown for three intervention half-life levels. The dotted lines indicate when interventions were applied (beginning of June). The effect of the interventions was assessed by evaluating the yearly average *Pf*PR_0–99_ reduction in all ages relative to the year prior to deployment (first grey block). Two outcomes were assessed, depending on whether the average prevalence was calculated over the year following deployment (immediate follow-up), or over the third year following deployment (late follow-up). **B** Correlation between simulated true (horizontal axis) and predicted (vertical axis) *Pf*PR_0–99_ reduction with a GP emulator trained to predict the immediate impact of ATSBs. The GP emulator was trained in a cross-validation scheme (distribution of the Pearson correlation coefficient *r*^*2*^ shown in the boxplot) and validated on an out-of-sample test set (*r*^*2*^ left upper corner and grey diamond lower right corner of the boxplot). **C** Relationship between each normalized input parameter and the resulting *Pf*PR_0–99_ reduction predicted with the trained GP emulator. Each parameter was in turn varied within its defined ranges (Table 1) while other parameters were set to their average values. **D** Estimated CPU execution time for varying sizes of input parameter sets evaluated with OpenMalaria (black) and with the trained GP emulator (grey)

We developed a disease model and machine learning approach to quantitatively define malaria interventions. Our approach consists of three components: “Disease modelling”, “Machine learning”, and “Target product profiles” (Fig. 2). The “Disease modelling” component of our approach included the results of iterative consultations with product development experts to build sensibly informed TPP simulation scenarios, i.e., to define the breadth, range, and intervention profiles to simulate with OpenMalaria for the five malaria interventions considered, as well as a public health goal to optimize (see “Methods” and Additional file 1: Sect. 1 for a description of the iterative stakeholder engagement process). Following the expert discussions, the public health goal chosen in this study was to reduce the prevalence of *Plasmodium falciparum* malaria (denoted as *Pf*PR_0–99_ when evaluated for all ages and *Pf*PR_2–10_ when evaluated for 2‒10-year-old) between years one and three following deployment (Fig. 3A). Table 1 summarizes the results of the stakeholder discussions to set-up the OpenMalaria simulation scenarios and presents a comprehensive description of all subsequent intervention characteristics explored in this study, as well as the simulated malaria transmission settings.

We used a previously published calibration of the OpenMalaria model (Figs. 1 and 3A, and Additional file 1: Sect. 1 and Tables S1.1‒S1.3), which reflects demographics, epidemiology, entomology, health system access, and seasonality (Additional file 1: Fig. S2.1) for a catchment area in Tanzania [55]. Intervention impact was assessed through predicted reduction in *Pf*PR_0–99_, corresponding to true infection prevalence and not patent [detected with a diagnostic such as rapid diagnostic test (RDT) or polymerase chain reaction (PCR), Fig. 3A and Additional file 1: Figs. S2.3 and S3.1–S3.4]. The simulated settings covered a broad spectrum of transmission and mosquito biting behavior archetypes relevant for attaining general guiding principles in the early development phase of new malaria interventions. A comprehensive set of simulated scenarios was built by uniformly sampling the parameter space defined by intervention and transmission setting characteristics (defined in Fig. 2A and detailed in Table 1). These scenarios were simulated with OpenMalaria, yielding an extensive database of disease outcomes for the defined scenarios.

In the machine learning part of the approach (illustrated in the *“Machine learning”* panel of Fig. 2A), the database of simulated scenarios and corresponding outcomes (*Pf*PR_0–99_ reductions following intervention deployment) was used to train predictive models for the OpenMalaria simulation results (for an example of simulated *Pf*PR_0–99_ time series with OpenMalaria see Fig. 3A and Additional file 1: Fig. S3.1). A Heteroskedastic Gaussian process (GP) model was trained for each intervention and transmission setting (see detailed training procedure in Additional file 1: Sect. 2.3). Trained GP models accurately captured the dependencies between the disease model input parameters and the output intervention impact (Fig. 3B and C) and were able to reliably predict the reduction in *Pf*PR_0–99_ attributable to any input intervention characteristics in a given malaria transmission setting. Precisely, the correlation between true and predicted *Pf*PR_0–99_ reduction on out-of-sample test sets exceeded 95% while the absolute mean error was below 3% for all trained GP models (Fig. 3B and Additional file 1: Figs. S4.1‒S4.3 and Table S4.1). As a result, the trained predictive GP models acted as emulators of the detailed modelled parameter dynamics and non-linear relationships within the individual-based mathematical model of malaria transmission (Fig. 3C and Additional file 1: Figs. S4.5‒S4.6) and could predict the disease outcome for the given health goal and for any set of input parameters.

Due to the significantly less intensive computational requirements of our emulator-based approach compared with OpenMalaria, we could reduce the analysis execution time by several orders of magnitude. This allowed us to conduct global sensitivity and optimization analyses, which required a large number of parameter set evaluations and would otherwise not have been possible (Fig. 3D and Additional file 1: Fig. S4.7). Thus, the trained GP emulators could be efficiently and promptly used in downstream analyses to explore the multi-dimensional space of intervention properties to design TPPs of new malaria interventions (panel *“Target product profiles”* in Fig. 2A), i.e., to identify the drivers of their impact and their quantitative properties in meeting the health goals previously defined (Fig. 2B and C). Specifically, through global sensitivity analysis, we identified the key determinants of intervention impact (Fig. 2B). In addition, we performed a constrained search for intervention and delivery profiles (TPPs) that maximized impact under a particular health goal, given concrete, expert-informed, operational constraints such as possible deployment coverage, or feasible intervention properties such as efficacy or duration of protection (Fig. 2C). Results of these analyses are detailed in the following sections and illustrated for seasonal transmission settings with high indoor mosquito biting. Results for the other simulated transmission settings (perennial settings and for other mosquito biting patterns) are provided in the supplement (additional sensitivity analysis results presented in Additional file 1: Figs. S5.1‒S5.2, additional optimization results presented in Additional file 1: Figs. S6.1‒S6.6 and S7.1‒S7.5) and summarized in Table 2.

**Table 2:** Key findings of our quantitative approach guiding target product profiles of new malaria interventions

Intervention	Summary of analysis results
Immunological interventions -Anti-infective monoclonal antibodies -Anti-infective vaccines -Transmission-blocking vaccines	*Key determinants of impact* (Fig. 4, Additional file 1: Fig. S5.1) -The main driver of intervention impact was coverage -The second determinant of intervention impact depended on intervention half-life. For interventions with short half-lives such as monoclonal antibodies, the half-life was the second driver, while for long-term interventions such as vaccines, efficacy played a key role -As opposed to long-term vaccines whose impact was mainly driven by coverage and efficacy, interventions with short half-lives (e.g., anti-infective monoclonal antibodies) relied on case management to prevent resurgence -The various biting patterns of mosquitoes did not influence the intervention determinants of impact
	*Optimal intervention profiles* (Fig. 5, Additional file 1: Fig. S6.1‒S6.4 and S7.1‒S7.3) -As opposed to vaccines, anti-infective monoclonal antibodies required high efficacy and deployment coverage while achieving limited reduction in *Pf*PR_0–99_ with very little impact in perennial settings -Increasing the deployment frequency for anti-infective monoclonal antibodies from once to twice per year, extended the landscape of feasible health targets mainly in seasonal settings -Combination with a blood-stage drug proved more impactful compared with increasing the deployment frequency for anti-infective monoclonal antibodies, extending the achievable health goals in perennial settings as well
Vector control interventions -Attractive targeted sugar baits -Eave tubes	*Key determinants of impact* (Fig. 4, Additional file 1: Fig. S5.2) -As with short-term immunological interventions, attractive targeted sugar baits relied on case management to prevent resurgence -Limited difference between key drivers for attractive targeted sugar baits in different mosquito biting settings was observed because mosquitoes sugar feed before biting indoors or outdoors -It was observed that intervention properties of eave tubes rather than health system access were larger drivers of impact in high indoor biting settings, as mosquitoes in those settings will be more likely to contact the eave tube
	*Optimal intervention profiles* (Figs. 3 and 6, Additional File 1: Figs. S6.5, S6.6, S7.4, and S7.5) -Increasing deployment frequency from once to twice per year for attractive targeted sugar baits resulted in a significant increase in intervention impact and less requirements in terms of coverage and half-life -Increasing efficacy of attractive targeted sugar baits did not have a significant impact

### Impact of malaria interventions and the importance of their characteristics

**Fig. 4:**
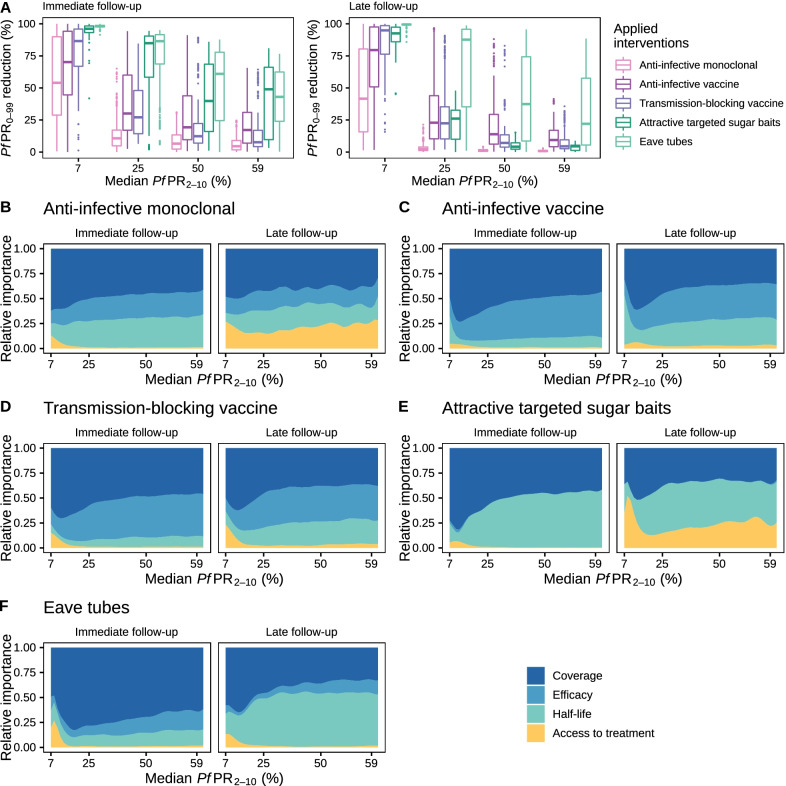
Effects of novel malaria interventions on *Pf*PR_0–99_ and their key drivers of impact. **A** Distribution of obtained reduction in *Pf*PR_0–99_ across the simulated scenarios with OpenMalaria following deployment of various malaria interventions under development (shown with different colors) for a range of simulated transmission settings (specified by median true *Pf*PR_2–10_ rounded values, x-axis). Each boxplot displays the interquartile range (box), the median value (horizontal line), the largest and smallest values within 1.5 times the interquartile range (whiskers), and the remaining outside values (points) of the *Pf*PR_0–99_ reduction values obtained across all the simulations for each given setting. The remaining panels present the results of global sensitivity analysis showing, across the same simulated *Pf*PR_2–10_ settings, the contribution of intervention characteristics to the resulting *Pf*PR_0–99_ reduction for anti-infective monoclonal antibodies (**B**), anti-infective vaccines (**C**), transmission-blocking vaccines (**D**), attractive targeted sugar baits (**E**), and eave tubes (**F**)

Following simulation with OpenMalaria of deployment of each of the studied interventions through mass administration campaigns over several years (see “Methods”), we analyzed the predicted distributions of reduction in true *Pf*PR_0–99_. We found that, in general, when aiming for substantial, prompt reductions in prevalence for this particular health target, vector control was by far the most impactful intervention across all settings. Conversely, monoclonal antibodies, anti-infective and transmission-blocking vaccines had a more pronounced impact in low-transmission settings compared to endemic settings (Fig. 4A and Additional file 1: Figs. S3.1–S3.4 and Table 2). Figure 4A displays the reductions of *Pf*PR_0–99_ for all the five interventions in a seasonal malaria transmission setting, with high indoor mosquito biting, for both immediate follow-up (during the year after intervention deployment) and late follow-up (during the third year after intervention deployment). Accordingly, at immediate follow-up, attractive targeted sugar baits achieved a median *Pf*PR_0–99_ reduction of 96% in low transmission settings (*Pf*PR_2–10_ ≈ 7%) decreasing to a median *Pf*PR_0–99_ reduction of 49% in high transmission settings (*Pf*PR_2–10_ ≈ 59%). Similarly, eave tubes reduced *Pf*PR_0–99_ by 98% (median value) in low transmission settings, decreasing to 43% reduction (median value) in high transmission settings. Monoclonal antibodies yielded the smallest impact compared to all the five tested interventions, reducing *Pf*PR_0–99_ by 54% (median value) in low transmission settings to only 4.5% (median value) in high transmission settings. Anti-infective vaccines reduced *Pf*PR_0–99_ by 70% (median value) in low transmission settings and by 17% (median value) in high transmission settings. Transmission-blocking vaccines reduced *Pf*PR_0–99_ by 86.5% (median value) in low transmission settings and by 8% (median value) in high transmission settings. At long follow-up, long-lasting eave tubes maintained the high reductions in prevalence, ranging from 99% (median value) in low transmission settings to 23% (median value) in high transmission settings. Attractive targeted sugar baits, anti-infective and transmission-blocking vaccines led to similar reductions (e.g., 79.5% median reduction for anti-infective vaccines in low transmission settings and 9% in high transmission settings), while monoclonal antibodies only achieved a maximum of 41.5% median *Pf*PR_0–99_ reduction in the low transmission settings, decreasing below 1% reduction in high transmission settings.

Sensitivity analysis indicated that the impact of these interventions on malaria prevalence was driven by different characteristics of their efficacy profiles, deployment strategies, or access to care for treatment of clinical cases, for short- and long-impact follow-up. Across a large proportion of the simulated scenarios, for all parasite and vector targets and interventions, deployed intervention coverage was overwhelmingly the primary driver of impact, especially in low-transmission settings (Fig. 4B–F and Additional file 1: Figs. S5.1–S5.2). For immunological interventions, the impact of short-term passive immunizations such as monoclonal antibodies relied on their deployment coverage and the health system (Fig. 4B and Additional file 1: Fig. S5.1). In contrast, for long-acting interventions such as vaccines, impact was driven by deployment coverage and efficacy (Fig. 4C and D and Additional file 1: Fig. S5.1). Highly efficient vector control interventions such as attractive targeted sugar baits had a strong effect on prevalence (Fig. 4A), and their duration of effect was the most important determinant (Fig. 4E and Additional file 1: Fig. S5.2). The immediate impact of long-term vector control interventions such as eave tubes was driven by deployment coverage, while their half-life was a key determinant for preventing resurgence (Fig. 4F and Additional file 1: Fig. S5.2). Determinants of impact were identified for both immediate and late follow-up when interventions were applied once per year for three years. A detailed description of the determinants of impact affecting the effectiveness of each intervention is provided in Table 2 (rows labelled “Key determinants of impact”).

### Minimal requirements of novel malaria interventions to achieve a defined health goal

**Fig. 5:**
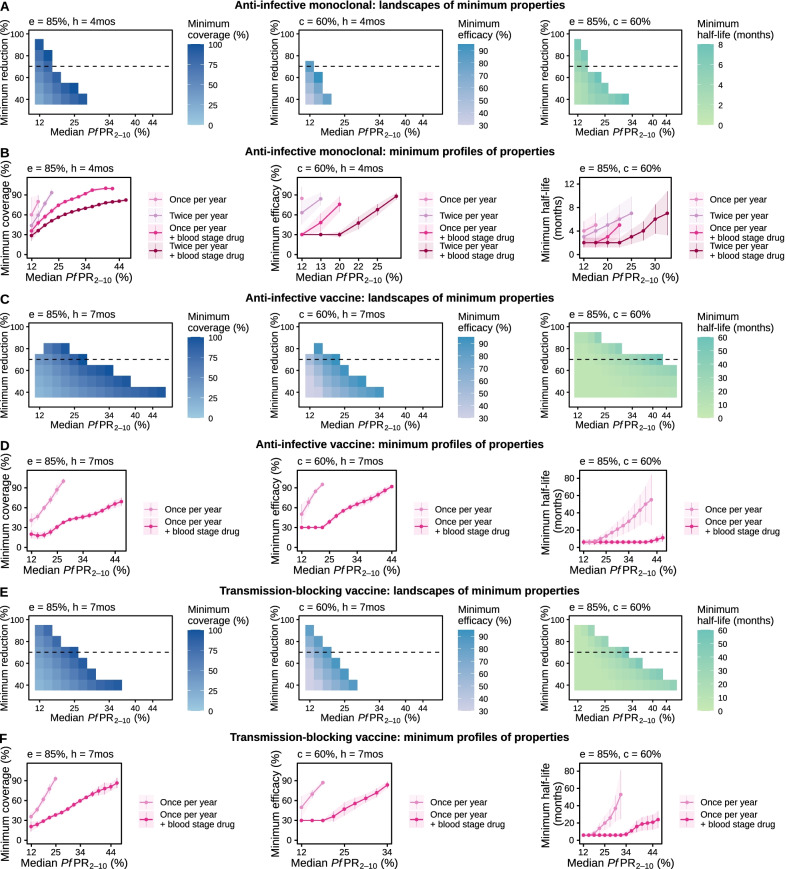
Estimated optimal intervention TPPs for immunological interventions. The heatmaps in panels (**A**), (**C**) and (**E**) display, for each intervention property (coverage, efficacy, or half-life), the landscape of minimum required values to achieve various target *Pf*PR_0–99_ reductions (y-axis) across different simulated transmission settings (true *Pf*PR_2–10_ rounded values, x-axis). Each row of the heatmap corresponds to a target of *Pf*PR_0–99_ reduction and constitutes the minimum required profile of the considered intervention. For a health goal of 70% *Pf*PR_0–99_ reduction (dotted line on each heatmap), panels (**B**), (**D**), and (**F**) present in detail how the minimum profile changes with transmission intensity. Each intervention characteristic was minimized in turn, while keeping other characteristics fixed (values marked on each panel where c = coverage, e = efficacy, and h = half-life). The simulated access to treatment, corresponding to a probability of seeking care within 5 days, was 25%. TPP = Target Product Profiles, mos = months

**Fig. 6:**
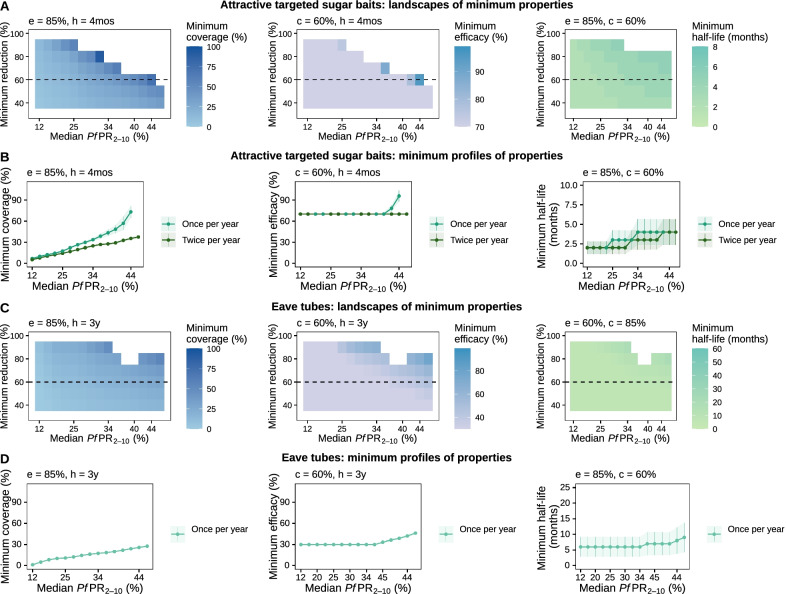
Estimated optimal intervention TPPs for vector control interventions. The heatmaps in panels (**A**) and (**C**) display, for each intervention property (coverage, efficacy, or half-life), the landscape of minimum required values to achieve various target *Pf*PR_0–99_ reductions (y-axis) across different simulated transmission settings (true *Pf*PR_2–10_ rounded values, x-axis). Each row of the heatmap corresponds to a target of *Pf*PR_0–99_ reduction and constitutes the minimum required profile of the considered intervention. For a selected health goal of 60% *Pf*PR_0–99_ reduction (dotted line on each heatmap), panels (**B**) and (**D**) present in detail how the minimum profile changes with transmission intensity. Each intervention characteristic was minimized in turn, while keeping the other characteristics fixed (values marked on each panel where c = coverage, e = efficacy, and h = half-life). The simulated access to treatment, corresponding to a probability of seeking care within 5 days, was 25%. TPP = Target Product Profiles

For the five aforementioned malaria interventions, we explored their optimal properties for a broad set of *Pf*PR_0–99_ reduction targets, creating landscapes of intervention profiles according to their minimal characteristics across various transmission settings (Fig. 5 and 6 and Additional file 1: Figs. S6.1‒S6.6 and S7.1‒S7.5). These landscapes provide a comprehensive overview of the intervention potential capabilities and limitations in achieving a desired health goal. As opposed to an anti-infective monoclonal antibody which required high efficacy and duration to achieve large *Pf*PR_0–99_ reductions in only a limited number of settings (Fig. 5A and B and Additional file 1: Figs. S6.1 and S6.2), attractive targeted sugar baits that kill mosquitoes also achieved a wider range of target *Pf*PR_0–99_ reductions in high-transmission settings (Fig. 6A and B and Additional file 1: Fig. S6.5). Similarly, while anti-infective and transmission-blocking vaccines had comparable requirements in achieving similar *Pf*PR_0–99_ reduction targets in settings with lower transmission (*Pf*PR_2–10_ < 30%), anti-infective vaccines showed a higher potential and reached additional targets in high-transmission, endemic settings (Fig. 5C–F).

For a detailed overview of landscapes of intervention profiles for all simulated settings and interventions see Additional file 1: Figs. S6.1‒S6.6. These landscapes together with results of the sensitivity analysis offer an evidence-based prioritization of resources during product development. We found that while both efficacy and half-life were important for immediate prevalence reductions with monoclonal antibodies, their effect was limited in preventing resurgence and was only supported by high case-management levels (Figs. 4 and 5 and Additional file 1: Figs. S5.1 and S6.1‒S6.2). Conversely, the efficacy of anti-infective vaccines determined their immediate impact, whereas half-life of effect had greater importance for achieving and maintaining *Pf*PR_0–99_ reductions (Figs. 4 and 5 and Additional file 1: Figs. S5.1, S6.3, and S6.4).

Our analysis showed that coverage was a primary driver of impact (Fig. 4B‒F and Additional File 1: Figs. S5.1 and S5.2). This has important implications for interventions requiring multiple applications to achieve high efficacy, indicating that it is of crucial importance to target both vulnerable populations and the proportion of the population missed by the intervention. While, for some interventions, high coverage deployment might be difficult or impossible to achieve, our analysis showed that this can be alleviated by increasing the deployment frequency or through deploying combinations of interventions, which may also have cost implications (Figs. 5B, D, F, 6B and Additional file 1: Figs. S6.1‒S6.5 and S7.1‒S7.4).

We found that combining several interventions targeting different stages in the transmission cycle can strongly affect the minimum requirements of a putative new intervention, potentially increasing the impact of an otherwise weaker intervention. For an anti-infective monoclonal antibody with an initial half-life of 4 months that is deployed at a coverage of 60% reflecting completion of multiple doses, achieving 80% prevalence reduction was impossible when deployed once yearly for three years (Fig. 5A, and Additional file 1: Fig. S6.1). Furthermore, achieving the aforementioned health goal required an efficacy of over 80% when the intervention was deployed twice per year for three years (Additional file 1: Fig. S6.2). However, when monoclonal antibody deployment was coupled with a short half-life blood-stage parasite treatment such as dihydroartemisinin-piperaquine or artemether-lumefantrine, its minimum required efficacy was considerably reduced for both delivery frequencies (Fig. 5B and Additional file 1: Figs. S6.1, S6.2, and S7.1). Conversely, if an initial efficacy of 85% for the monoclonal antibody was assumed, its minimal required half-life could be reduced if this intervention was deployed in combination with a blood-stage parasite-clearing drug (Fig. 5B and Additional file 1: Figs. S6.1, S6.2, and S7.1). These results partly motivated the current development of anti-infective monoclonal antibodies; use-cases will likely include deployment with existing or new antimalarial treatment.

When coupled with a short half-life blood-stage parasite treatment, requirements of coverage, efficacy and half-life were also reduced for anti-infective and transmission blocking vaccines to achieve targeted reductions of *Pf*PR_0–99_ (Fig. 5C–F and Additional file 1: Figs. S6.3, S6.4, S7.2, and S7.3). In particular, for high-transmission settings (*Pf*PR_2–10_ > 30%), given an RTS,S-like half-life of seven months, both anti-infective and transmission-blocking vaccines could not achieve a defined prevalence reduction goal of 70% if deployed singly (Figs. 5D and F). This was the case for any deployment coverage given an initial efficacy of 85%, as well as for any efficacy given a 60% deployment coverage. Combining vaccine deployment with a blood-stage drug not only significantly expanded the achievable health targets in high-transmission settings, but also reduced vaccine properties requirements. Our analysis revealed that anti-infective vaccines had a higher potential than transmission-blocking vaccines, requiring less performance and achieving higher prevalence reductions targets in higher transmission settings. When combined with blood-stage parasite treatment, the coverage, efficacy, and half-life requirements of anti-infective vaccines were lower compared with those of transmission-blocking vaccines for the same prevalence reduction targets (Fig. 5 and Additional file 1: Figs. S6.3, S6.4, S7.2, and S7.3).

We also showed that a modified deployment schedule could reduce requirements for properties of some interventions. For highly efficacious attractive targeted sugar baits, higher coverage and half-life were required when implemented once per year for three years compared with accelerated delivery of twice per year for three years (Fig. 6A and B). Except for high-transmission settings (*Pf*PR_2–10_ > 41%), a required efficacy of 70% was sufficient to attain the desired health goal for the majority of settings, for both delivery schedules (Fig. 6A and B, and Additional file 1: Figs. S6.5 and S7.4). This result was also reflected in the sensitivity analysis (Fig. 4E). Accordingly, the variation in intervention efficacy, across its investigated ranges, had little importance in driving the intervention impact. This suggests that, once a vector control intervention, such as attractive targeted sugar baits, has achieved a high killing efficacy (here ≥ 70%), a next step of optimizing other intervention characteristics, such as deployment coverage or duration, would lead to higher impact.

Our comprehensive analysis was applied to explore determinants of impact and required profiles of interventions across two seasonal settings (seasonal and perennial) and three types of indoor mosquito biting patterns (low, medium, and high). A detailed overview of impact determinants and optimal intervention profiles is presented in Additional file 1: Figs. S6.1‒S6.6 and S7.1‒S7.5, with additional key results summarized in Table 2.

## Discussion

In this study, we introduced a quantitative framework, using detailed simulation models of malaria transmission dynamics that enables for the first time a quantitative differentiation between operational, transmission setting, and intervention parameters to better understand the potential impact of novel interventions against malaria. The framework consists of (i) a comprehensive disease progression and transmission simulation model applied on a discrete, uniformly sampled set of input parameters; (ii) training of a GP emulator on the sampled set of parameters and corresponding impact outcomes; (iii) using sensitivity analysis to understand drivers of intervention impact; and (iv) applying a non-linear constrained optimization algorithm to explore intervention operational and effectiveness characteristics meeting various targets and deployment use-cases specified following iterative consultation with product development experts. Our work thus builds on recent applications of GPs in disease modelling and burden prediction for malaria [56].

The value of our approach is realized through iterative collaboration with product development experts, by providing model-based guidance throughout the development process, and by refining feedback on model predictions as interventions progress through development. For malaria, where multiple interventions are in development, it also offers an approach for product developers from diverse fields (such as therapeutics and insecticide development) to collaborate and incorporate knowledge of other interventions into their TPP development. The exchanges with stakeholders ensured a crucial discussion environment, guiding and supporting the methodology at various levels, from intervention profiling and defining relevant intervention use-cases to shaping research questions and subsequent analyses. Consequently, iterative exchanges with stakeholders have not only shaped the study approach, but have proven the value of this methodological framework in its versatility to adapt and address key questions along the product development pathway.

This quantitative framework can support the development of interventions from the beginning by generating evidence to inform and define evaluation criteria ensuring new products meet relevant health targets, while considering how these products may affect disease burden and epidemiology within a population. As shown here, this relies on iterative dialogue with stakeholders, to first define health targets, simulated scenarios, achievable intervention properties, and operational settings. The modelling part of the framework incorporates all this information as well as relevant disease transmission dynamics, building an *in-silico* system for testing developed interventions.

Through the sensitivity analysis part of the framework, for the five malaria interventions considered, our analysis showed which intervention characteristics drive impact and are thus crucial in achieving the defined health goal. Investigating how the importance of the various intervention characteristics changes across transmission and follow-up provides insights on the development processes to be prioritized. Our findings suggest that if monoclonal antibodies were to support preventing resurgence, then R&D efforts should focus on increasing and establishing antibody longevity. While bringing valuable quantitative insights to guide product development, our analysis of novel malaria interventions reproduces previous findings concerning intervention characteristics that are key drivers of impact. Previous studies have shown that intervention coverage is a major determinant of impact in the context of mass drug administration [41], of vaccines [57], as well as of vector control [26]. Furthermore, this analysis reaffirms previous work showing the ability of vector control interventions to achieve substantial reductions in malaria burden [58].

The optimization analysis part of the framework reveals the potential of the developed intervention and how its efficacy and coverage requirements change according to the defined health targets and deployment setting. The landscapes of intervention profiles help product developers gauge development and investment efforts and select promising products. Furthermore, our approach allows investigating combinations of new and existing interventions, identifying alternatives to alleviate shortcomings such as coverage limitations. To achieve a final TPP, several iterations of this analysis are required, to ensure that the optimal tradeoffs between intervention capabilities and target goals for a given setting are best achieved.

Using a detailed individual-based malaria transmission model like OpenMalaria brings significant advantages compared to a simpler and computationally less intensive Ross-MacDonald model. Individual-based models capture interactions between hosts, vectors, and parasites at the individual level, which provides a more realistic representation of the nonlinear transmission and epidemiological processes in the population as well as of the stochasticity of the modelled system [59]. Second, it allows a realistic implementation of interventions, allowing for explicitly modeling intervention characteristics relevant for defining TPPs such as deployment regimes, efficacy, half-life, or shape of decay as well as explicit action at the different stages of the transmission cycle. The advantage of the machine learning layer of our approach consists in building a simplified approximation of the relationships between intervention characteristics and resulting intervention impact. This approximation is very specific to the varied components across the model simulations (intervention characteristics, access to treatment and EIR) and to the resulting *Pf*PR_0–99_ reduction and allows significantly reducing the computational costs of running an individual-based model to explore the high-dimensional space of intervention characteristics.

Although in this analysis we used reduction of *Pf*PR_0–99_ as a health goal, this method can be applied to other continuous disease burden statistics as required (see Additional file 1: Fig. S4.4 for performance predicting malaria incidence reduction). The same rationale applies for investigating other deployment strategies, required doses of interventions, or additional intervention combinations. However, this approach, which uses a smooth GP model, is not tailored for classification and categorical health goals. Nevertheless, it could be adapted to these types of outputs by replacing the GP emulator with a predictive model/alternative algorithm suited for categorical data, such as support vector machines. While sensitivity analysis would still be applicable for identifying the drivers of the categorical outcomes, the optimization questions and analyses would need to be reformulated to be relevant for the chosen categorical outcomes.

As with all modelling studies, this approach is based on the model assumptions, simulation setup and parameterizations, thus the presented results are specific to this design. Furthermore, while the emulators capture not just the mean tendency of disease models dynamics, but also the inherent output variance caused by the stochasticity in the models [60], the estimations provided in this study are dependent on the performance of the trained emulator. This challenge was addressed with extensive adaptive sampling and testing to ensure a high level of accuracy of the trained emulators (Fig. 3, and Additional file 1: Figs. S4.1‒S4.3 and Table S4.1). Despite the intrinsic uncertainty, this framework is intended to provide guiding principles and an efficient means of exploring the high dimensional space of intervention characteristics that otherwise would not be possible. Evidently, this analysis relies on the representativeness of model assumptions of disease and transmission dynamics as well as of expert opinion of likely intervention parameterizations in absence of clinical knowledge. Lastly, this analysis only explored a subset of use-cases, transmission settings, and intervention combinations. Future work should focus on the most likely settings and relevant use-cases as interventions are being developed and corresponding TPP documents are being refined.

Moving beyond the work presented in this paper, this framework would allow combining simulation models with other sources of data describing geographical variation in disease, for example, modelled health systems or modelled prevalence [61] and would allow incorporating interactions of interventions with novel interventions for surveillance. Clinical trials for new interventions could thereby be prioritized to geographical settings, where public health impact is likely to be maximized, and where appropriate, to inform decisions on achieving non-inferiority or superiority endpoints [62]. A significant extension would be to incorporate economic considerations that may affect development decisions, including both R&D costs, as well as implementation and systems costs for final deployment.

## Conclusions

In this work, we provide mathematical tools for efficiently and quantitatively defining the minimum profiles of malaria interventions, as well as delivery approaches required to reach a desired health goal. Our framework can be extended and used for any disease where a valid model of disease progression or natural history of disease is available. It can be used to direct the design of novel interventions and to better understand how intervention-specific, epidemiological and systems factors jointly contribute to impact. Most immediately, this approach is highly relevant to define successful interventions against new diseases, and to support efficient, fast development of operational strategies.

Our framework tackles and moves beyond current challenges in product development. On one hand, it allows rigorous definition of TPPs by efficiently exploring high dimensional parameter spaces of disease dynamic models and the interventions, and on the other hand, it allows determinants of desired public health impact to be identified to inform tradeoffs between product characteristics and use-cases.

## Supplementary Information

**Additional file 1.** Includes additional methods, figures, and tables that complement the analysis and results presented in the main manuscript.

## Data Availability

All analysis code used for this study, as well as corresponding documentation, parameterizations, and configuration files for the software workflow necessary to generate the simulation data with OpenMalaria and to reproduce this analysis are available in the TPP_workflow repository, https://github.com/SwissTPH/TPP_workflow.

## Abbreviations

ATSB: Attractive targeted sugar bait
BMGF: Bill and Melinda Gates Foundation
EIR: Entomological inoculation rate
GP: Gaussian process
IPT: Intermittent preventive treatment
IVCC: Innovative Vector Control Consortium
PATH-MVI: Program for Appropriate Technology in Health—Malaria Vaccine Initiative
PCR: Polymerase chain reaction
*Pf*PR: *P**lasmodium falciparum* Prevalence of infections
R&D: Research and development
RDT: Rapid diagnostic test
SMC: Seasonal malaria chemoprevention
TPP: Target product profiles
WHO: World Health Organization
