# Supplementary material for: Leveraging mathematical models of disease dynamics and machine learning to improve development of novel malaria interventions
Source: Infect Dis Poverty. 2022 Jun 4;11:61. doi: 10.1186/s40249-022-00981-1 (PMC9167503; doi:10.1186/s40249-022-00981-1)
Supplement: Supplementary file 1 — Additional file 1. Includes additional methods, figures, and tables that complement the analysis and results presented in the main manuscript. [file 40249_2022_981_MOESM1_ESM.pdf]

Additional file 1

Leveraging mathematical models of disease dynamics and machine learning to improve development of novel disease interventions

Monica Golumbeanu[1,3]*, Guojing Yang[1,2,3]*, Flavia Camponovo[1,3,4], Erin M. Stuckey[5], Nicholas Hamon[6], Mathias Mondy[6], Sarah Rees[6], Nakul Chitnis[1,3], Ewan Cameron[7,8,9], and Melissa A. Penny[1,3]†

[1]Swiss Tropical and Public Health Institute, Basel, Switzerland
[2]Key Laboratory of Tropical Translational Medicine of Ministry of Education and School of Tropical Medicine and Laboratory Medicine; the First and Second Affiliated Hospital of Hainan Medical University; Hainan Medical University, Haikou, Hainan, P. R. China
[3]University of Basel, Basel, Switzerland
[4]Center for Communicable Disease Dynamics, Department of Epidemiology, Harvard T. H. Chan School of Public Health, Boston, MA 02115, USA
[5]The Bill & Melinda Gates Foundation, Seattle, WA, USA
[6]Innovative Vector Control Consortium, Liverpool, United Kingdom
[7]Malaria Atlas Project, Big Data Institute, University of Oxford, Oxford, UK
[8]Curtin University, Perth, Australia
[9]Telethon Kids Institute, Perth Children's Hospital, Perth, Australia
*These authors contributed equally.
†Correspondence to: melissa.penny@unibas.ch

Table of contents

1 Stakeholder engagement, role of TPP, model description, and interventions 3
1.1 Stakeholder engagement 3
1.2 Description of the OpenMalaria model of malaria transmission dynamics 5
1.2.1 Individual-based model of malaria transmission 5
Table S1.1. Overview of the OpenMalaria model components 6
Table S1.2. Estimated core parameters of OpenMalaria describing the natural history of the disease in human hosts 9
Table S1.3. OpenMalaria model parameters describing the dynamics of the mosquito feeding cycle. 11
1.2.2 Calibration of the disease model and description of simulation experiments. 11
1.2.3 Definition of intervention profiles. 12
1.2.4 Translation of input EIR to *Pf*PR$_{2-10}$ and *Pf*PR$_{0-99}$ 13
1.2.5 Definition of intervention impact and health goals 13
2 Disease scenarios 15
Fig. S2.1. Illustration of the yearly malaria transmission and prevalence patterns in simulated seasonal settings. 15
Fig. S2.2. Representation of decay and the range of efficacy and half-life against different parasite or vector targets for intervention-agnostic malaria interventions. 16
Fig. S2.3. Simulated distributions of true and patent (detected with PCR or RDT) *Pf*PR$_{0-99}$ and *Pf*PR$_{2-10}$ for various input EIR levels in absence of interventions. 18

Table S2.1. Specifications of the optimization procedure for TPP development 19
2.1 Building a disease model emulator with Gaussian processes 20
2.2 Training data 20
2.3 Gaussian process emulators 21
3 Results: Disease model simulation 22
Fig. S3.1. Examples of OpenMalaria simulation outputs. 22
Fig. S3.2. Distributions of prevalence reduction following yearly deployment of single interventions. 23
Fig. S3.3. Distributions of prevalence reduction following yearly deployment of combinations of interventions. 24
Fig. S3.4. Distributions of prevalence reduction following deployment of single and combinations of interventions twice per year. 25
Fig. S3.5. Simulations reaching malaria elimination before intervention deployment. 26
4 Sensitivity analysis and emulator performance results 27
4.1 Identifying impact determinants through sensitivity analysis 27
4.2 Results: Sensitivity analysis and emulator performance 28
Fig. S4.1. Assessment of the performance of the trained GP depending on the training set size. 28
Fig. S4.2. Performance of the trained GP emulators predicting immediate intervention impact. 29
Fig. S4.3. Performance of the trained GP emulators predicting long-term intervention impact. . 30
Fig. S4.4. Performance of trained GP emulators predicting incidence reduction........................ 31
Fig. S4.5. Relationships between input intervention parameters and the predicted immediate *Pf*$PR_{0\text{-}99}$ reduction with the trained GP emulator. 32
Fig. S4.6. Relationships between input intervention parameters and the predicted long-term *Pf*$PR_{0\text{-}99}$ reduction with the trained GP emulator. 33
Fig. S4.7. Execution time of OpenMalaria simulations and GP emulator training. 34
Table S4.1. Performance of the trained GP emulators predicting immediate and long-term intervention impact 35
5 Finding minimal intervention properties and results for key determinants of impact 36
5.1 Finding minimal intervention properties 36
5.2 Results: Key determinants of impact 38
Fig. S5.1. Key drivers of impact for immunological malaria interventions across different transmission settings. 38
Fig. S5.2. Key drivers of impact for vector control malaria interventions across different transmission settings. 39
6 Results: Feasible landscapes of optimal, constrained intervention profiles 40
Fig. S6.1. Feasible landscapes of optimal, constrained intervention profiles (TPPs) for an anti-infective monoclonal antibody deployed once per year. 40
Fig. S6.2. Feasible landscapes of optimal, constrained intervention profiles (TPPs) for an anti-infective monoclonal antibody deployed twice per year. 41
Fig. S6.3. Feasible landscapes of optimal, constrained intervention profiles (TPPs) for an anti-infective vaccine deployed once per year. 42
Fig. S6.4. Feasible landscapes of optimal, constrained intervention profiles (TPPs) for a transmission-blocking vaccine deployed once per year. 43
Fig. S6.5. Feasible landscapes of optimal, constrained intervention profiles (TPPs) for attractive targeted sugar baits deployed once or twice per year. 44

Fig. S6.6. Feasible landscapes of optimal, constrained intervention profiles (TPPs) for eave tubes deployed once per year. 45
7 Results: Optimal intervention profiles 46
Fig. S7.1. Optimal intervention profiles (TPPs) for anti-infective monoclonal antibodies under various deployment regimes to achieve a PfPR0-99 reduction of at least 70%. 46
Fig. S7.2. Optimal intervention profiles (TPPs) for anti-infective vaccines under various deployment regimes to achieve a PfPR0-99 reduction of at least 70%. 47
Fig. S7.3. Optimal intervention profiles (TPPs) for transmission-blocking vaccines under various deployment regimes to achieve a *Pf*PR$_{0\text{-}99}$ reduction of at least 70%. 48
Fig. S7.4. Optimal intervention profiles (TPPs) for attractive targeted sugar baits under various deployment regimes to achieve a PfPR0-99 reduction of at least 70%. 49
Fig. S7.5. Optimal intervention profiles (TPPs) for eave tubes to achieve a PfPR0-99 reduction of at least 70%. 50

1 Stakeholder engagement, role of TPP, model description, and interventions

1.1 Stakeholder engagement

By offering a comprehensive snapshot of the development process at any given point in time, a TPP constitutes a vital reference for dialogue between various stakeholders to guide decisions on the development direction (1-5). A well-constructed TPP is thus essential for efficient resource allocation and success during development (4, 5). However, the process of establishing TPPs relies on minimal clinical or quantitative evidence. They are often set by expert opinion and consensus is based on limited quantitative consideration of the complex dynamics of disease or on predictions of the likely intervention impact while achieving the identified unmet health needs (6). Furthermore, few malaria TPPs consider operational aspects such as deployment coverage in addition to product-specific characteristics such as efficacy or half-life. This has implications for the appropriate definition of intervention effectiveness characteristics according to local health systems and health targets (4, 6). To ensure a to ensure a realistic representation of novel malaria interventions and of the simulated disease transmission settings in our approach, there was active engagement and regular communication exchanges with different expert groups during development of this methodological framework for guiding Target Product Profiles (TPPs) of novel malaria interventions.

We collaborated with several product development partnerships. For each new intervention in the multi-stakeholder portfolios, we conducted several expert group discussion sessions to catalogue the ranges of potential intervention effectiveness; potential delivery strategies; parasite or vector targets; and likely properties (age target, mass intervention, yearly deployment) in terms of mode of action (target), efficacy, and duration; and use-cases/delivery The stakeholders involved in these discussions were the Bill & Melinda Gates Foundation (BMGF), the Innovative Vector Control Consortium (IVCC), and the Program for Appropriate Technology in Health - Malaria Vaccine Initiative (PATH-MVI) and the WHO (7).

Regular presentations of this ongoing analysis were provided to BMGF. These presentations covered relevant model assumptions for implementing different interventions, focusing on aspects

where stakeholder feedback was needed, as well as intermediary results where model predictions for the interventions were shown. This ensured a dynamic iterative exchange where stakeholders were directly involved in shaping intervention characteristics, allowing them to see how their input was incorporated in this methodology (Table 1) and how these assumptions influenced the impact predictions of this analysis. These exchanges helped us design and refine the means of communication for this methodological approach and model predictions to a wider stakeholder audience. For this purpose, communication elements were used, such as tables summarizing the key quantitative results (e.g., Table 2), clear definitions of modeled intervention properties (see Additional file 1: section 1.2.3, and Table 1), schematic diagrams offering an intuitive representation for the methodological processes (Fig. 1 and 2), and a standardized system of plots showing results for the different interventions explored (Fig. 2-6). There were three types of standardized plots. First, sensitivity analysis plots (Fig. 2B and 4) displaying, for each intervention, the relative importance of the intervention characteristics in determining the impact on $Pf\mathrm{PR}_{0\text{-}99}$ reduction. Relative importance was calculated following global sensitivity analysis using Sobol' indices (see Additional file 1: section 4.1 for a detailed description and calculations). These plots facilitated visual communication of key drivers of intervention impact and importantly how these drivers changed across various transmission settings, across immediate versus late follow-up of intervention impact, as well as across interventions. Second, landscapes of minimum intervention properties plot displaying, for different malaria transmission levels, an overview of the minimum intervention requirements in terms of coverage, efficacy, and half-life for a wide range of target $Pf\mathrm{PR}_{0\text{-}99}$ reductions (Fig. 2C - left panel, Fig. 5A, C, and E and 6A and C) (see Additional file 1: section 5.1 for a detailed description of the optimization process). These plots allowed for communication of overall intervention capabilities in reducing malaria prevalence, as well as their minimal requirements across different malaria transmission settings and for a wide range of $Pf\mathrm{PR}_{0\text{-}99}$ reduction targets. These requirements defined the profiles of intervention characteristics. Third, minimum profiles of properties plot displaying for different malaria transmission levels, the estimated minimum intervention requirements in terms of coverage, efficacy, and half-life for a chosen $Pf\mathrm{PR}_{0\text{-}99}$ reduction target (Fig. 2C – right, 5B*,* D, and F*,* and 6B and D) (see Additional file 1: section 5.1 for a detailed description of the optimization process). These plots facilitated the visual communication of the minimal intervention profiles and their variance across a range of different transmission settings for a chosen $Pf\mathrm{PR}_{0\text{-}99}$ reduction target. Furthermore, they allowed direct comparison of the required intervention profiles between immediate and long follow-up, across different deployment regimes (once versus twice per year), as well as across different intervention strategies (medical interventions deployed separately or in combination with an antimalarial drug).

The communication elements established above were instrumental to present proof-of-concept and intermediate analyses. For example, an intermediate result was that the determinants of impact of novel interventions were different in low as compared with moderate transmission settings. This motivated exploring a wide range of *Pf*PR settings to ascertain how impact determinants were modulated as transmission increased. Another early result was that the efficacy, coverage, and half-life requirements of monoclonal antibodies and vaccines were significantly high, and that it was infeasible to reach high targets of prevalence reduction in high-transmission settings deploying these interventions alone. The subsequent discussions led to the exploration of deploying combinations of these interventions with an antimalarial drug and to refining target health goals based on the capabilities of the interventions to reduce burden. Results were presented

during several stakeholder meetings and included in reports along with outlining prospects for future work.

1.2 Description of the OpenMalaria model of malaria transmission dynamics

1.2.1 Individual-based model of malaria transmission

OpenMalaria is an individual-based model of malaria transmission that considers the natural history of malaria in humans linked with a deterministic, entomological model of the mosquito oviposition cycle and malaria transmission in mosquitoes (8, 9) (Table S1.1). The modelled transmission cycle (Fig. 1) considers the chain of processes following infection of a human host, simulating malaria infection in individuals and modelling infection characteristics such as parasite density, duration of infection, infectivity to mosquitoes, and health outcomes such as anemia, other morbidities, or mortality. OpenMalaria specifically captures heterogeneity in host exposure, susceptibility, and immune response, taking into consideration the effects of several factors such as acquired immunity, human demography structure, and seasonality (10-13). Furthermore, the model includes a detailed representation of the health system (14), and a wide range of human and vector control interventions while tracking multiple health outcomes over time (Fig. 1 and Table S1.1).

In OpenMalaria, the pattern of yearly malaria infection in the absence of interventions is determined by the input entomological inoculation rate (EIR) (Additional file 1: Fig. S2.1). Each simulated infected human host has an associated parasite density and duration of infection (modelled individually and capturing disease effects such as immunity, infectiousness to mosquitoes, morbidity, or mortality). Setting-specific characteristics such as population demographics, mosquito species entomological characteristics or seasonality are explicitly modelled and a wide range of human and vector interventions can be applied, affecting the transmission cycle at various stages. Various health outcomes are monitored over time, including *Plasmodium falciparum* prevalence of infections (*Pf*PR), uncomplicated clinical disease, severe disease in and out of hospital, and malaria mortality. Disease dynamics model assumptions have been described and validated with field data in previous studies, notably in (15) and very recently revisited in (16). Several major consensus modeling studies have shown the validity of OpenMalaria predictions regarding the public health impact of the RTS,S vaccine (17), mass drug administration (18), or vector control (19). Additional validation studies conducted using published data on various malaria indicators such as EIR, *Pf*PR, incidence, and mortality have shown that OpenMalaria accurately captures the seasonally-dependent relationships between these indicators (20).

OpenMalaria has been widely documented and validated against a multitude of field studies, compared to existing models, and used in extensive studies to provide evidence for the epidemiological effects of various interventions (15, 17, 21-24). It comprises 14 model variants based on distinct sets of assumptions of its epidemiology and transmission components (25). For the present analysis, the “base” simulation model was used. Mathematical equations of the OpenMalaria model, its assumptions, calibration, and validation have been thoroughly described in numerous publications (see the Background section), and are therefore not specified here, however, an overview of the key modelled disease processes and assumptions along with the

corresponding references where model descriptions have been elaborated are provided in Table S1.1. In the sections below, an overview of the calibration and simulation settings used for this study has been provided.

Table S1.1. Overview of the OpenMalaria model components

**Name and *references***	**Description of core assumptions**
Key modelled epidemiological processes (base model)	
**Malaria infection of humans** *(26) and eq. 1-4 of Additional file 1 in (15)*	- Determined by EIR which is a model input and affects the force of infection in the simulated setting<br>- Considers an age-dependent exposure of human hosts to mosquitoes (correlating with body-surface area)<br>- The relationship between infection rates and EIR is defined and fitted with data from The Gambia, Nigeria and Kenya in (26)
**Infection progression in humans: asexual parasite densities and immunity** *(10, 15, 26, 27) and eq. 5-15 of Additional file 1 in (15)*	- Blood-stage parasite density depends on the time since infection and is affected by naturally acquired immunity. Acquired immunity reduces parasite density of subsequent infections.<br>- The duration of infection follows a log-normal distribution and is estimated from a malaria therapy dataset ((27) and eq. 1 in (10))<br>- Immunity (both pre-erythrocytic and blood-stage) develops progressively following consequent episodes of exposure to infection and total parasitemia seen by an individual in their lifetime.<br>- Super-infection is possible with cumulative parasite densities<br>- The parasite density in a host at a given time is defined and fitted with data from Ghana, Nigeria and Tanzania in (10)
**Transmission from infected humans to mosquitoes** *(11, 15, 28) and eq. 16-21 of Additional file 1 in (15)*	- Infectivity to mosquitoes depends on the density of parasites present in the human (including a time-lag for gametocyte development)<br>- The fraction of resulting infected mosquitoes after feeding on a human host follows a binomial distribution<br>- The relationship between infectivity to mosquitoes and parasite density was defined and fitted in (11) with data from malaria therapy collected in Georgia between 1940 and 1963 and available from (27)<br>- The age-specific contribution to overall infectiousness to mosquitoes was validated in (11) against field data collected from Liberia, The Gambia, Tanzania, Kenya, Papua New Guinea and Cameroon.
**Clinical illness, morbidity, mortality, and anemia** *(12, 13, 15, 29) and eq. 22-32 of Additional file 1 in (15)*	- Acute clinical illness depends on human host parasite densities and their pyrogenic threshold which evolves over time depending on the individual exposure history<br>- Acute morbidity episodes can be uncomplicated or evolve to severe episodes; a proportion of the severe episodes leads to deaths<br>- The probability of a clinical malaria episode was defined and fitted with data from Senegal in (13)<br>- The probabilities that a clinical episode becomes severe and the risk of mortality for a severe episode are defined and fitted to field data from over 10 African countries in (12)
Modelled characteristics of the transmission setting	

**Population age structure** *(12, 30)*	- Informed by health and demographic surveillance data from Tanzania
**Transmission seasonality** *(20, 26)*	- Seasonally forced, the same transmission pattern is reproduced each year in absence of interventions, displayed in Fig. S2.1. Seasonal patterns are inputs to the model and users can define any patterns as needed e.g., perennial, one or two peak seasonal patterns, etc.
**Case management** *(14)*	- Modelled through a comprehensive decision tree-based model defined and validated in (14) which determines the corresponding treatment implications depending on the occurring clinical events such as fevers and seeking of care<br>- Its representation includes specification of access to official or non-official care, access to hospital for severe cases, diagnostic tests (use, specificity, sensitivity, and threshold of detection), treatments for first, second line and non-official care, effects of treatment, case fatality rate, case sequelae and cure rates
**Entomological setting** *(9)*	- Comprehensive simulation of the mosquito lifecycle and behavior towards human and animal hosts (biting, resting) embedded in a dynamic entomological model of the mosquito feeding cycle defined in (9)<br>- Multiple vector species can be simulated simultaneously
<u>Modelled interventions and their action</u>	
**Vector control** *(19, 31, 32)*	- Acts on the availability of the protected human hosts to mosquitoes (deterrence), on the probability that a mosquito bites a protected human host (preprandial effect) and on the probability that a mosquito survives host feeding (postprandial effect)
**Drugs and Vaccines** *(17, 33)*	- Act at various levels of the parasite life cycle (transmission blocking, anti-infective, blood-stage clearance) and their action is defined by their initial efficacy, half-life, and decay
**Deployment characteristics**	- Interventions can be deployed for several rounds to a targeted group of individuals and specified coverages (proportion of the population covered by the intervention)
<u>Simulation regimes and model variants</u>	
**Time steps**	- Simulation outputs are tracked every 5 days
**Model variants** (25)	- Varying assumptions in immunity decay, treatment and heterogeneity of transmission are covered in 14 model variants described in (25). In the present study, we use the base model (parameterization described in Table 2 in (25) under the denomination R0001)
<u>Software availability and documentation</u>	
Source code and wiki page available on GitHub: https://github.com/SwissTPH/openmalaria/	

The OpenMalaria individual based, stochastic model of malaria transmission in humans was originally developed in 2003-2006 (15), with mosquito dynamics updated in 2008 (8) and an additional 13 structural model variants developed and parameterized in 2012 (25). In this table we summarize the key model components and assumptions and reference the previous studies where these assumptions have been quantified, fitted, and validated using field epidemiological data.

Table S2.2. Estimated core parameters of OpenMalaria describing the natural history of the disease in human hosts

Parameter	Estimated value from (25) (model R0001)
Lower limit of success probability of inoculations at high EIR	0.049
Critical value of EIR	0.032 inoculations/person-night
Lower limit of success probability of inoculations in immune individuals	0.14
Critical value of cumulative number of entomological inoculations	1’514.4 inoculations
Steepness of relationship between success of inoculation and the critical value of cumulative number of entomological inoculations	2.04
Variation between hosts on parasite densities (variance of log-normal distribution)	10.174
Critical value of cumulative number of parasite days	3.52 parasite-days/µl x 10^{-7}
Critical value of cumulative number of infections	97.3 infections
Maternal protection at birth	0.90
Decay of maternal protection	2.53 per year
Fixed variance component for densities	0.66 $[\ln(\text{parasites}/\mu l)]^2$
Critical value of cumulative number of infections for variance in parasite densities	0.92 infections
Critical value in determining an increase of the pyrogenic threshold	6’502.3 parasites/µL
Factor determining an increase in the pyrogenic threshold	143’000 $\text{parasites}^2 \mu l^{-2} \text{day}^{-1}$
Density bias (non Garki)	0.177
Mass action parameter	1
Case fatality for severe episodes in the community compared to hospital	2.09
Co-morbidity intercept relevant to indirect mortality	0.019
Non-malaria intercept for infant mortality rate	49.5 deaths/1000 live births

Density bias (Garki)	4.796
Parasitemia threshold for severe episodes type B_1	784’000 parasites/µL
Immune penalty	1
Immune effector decay	1
Prevalence of co-morbidity/susceptibility at birth relevant to severe episodes	0.092
Decay rate of the pyrogenic threshold	2.5 per year
Critical value of parasite density in determining an increase in the pyrogenic threshold	0.6 parasites/µl
Asexual immunity decay	0
Pyrogenic threshold at birth	296.302 parasites/µl
Idete multiplier	2.798
Critical age of co-morbidity	0.117

These parameters have been previously estimated in (15) and (25) through model calibration (see Additional file 1: section 1.2.2, and Table S1.1) and were kept unchanged during all the simulations in the present study. EIR: entomological inoculation rate.

Table S1.3. OpenMalaria model parameters describing the dynamics of the mosquito feeding cycle.

Parameter	Value
Duration of the period when a mosquito is resting	3 days
Extrinsic incubation period (time required for sporozoite development)	11 days
Proportion of mosquitoes which are host seeking on same day as oviposition	0.313
Maximum proportion of the day that a mosquito spends host-seeking	0.33
Probability that a mosquito survives the feeding cycle	0.623
Probability that a mosquito successfully bites the chosen human host	0.95
Probability that a mosquito escapes the human host and finds a resting place after biting	0.95
Probability that a mosquito successfully rests after finding a resting site	0.99
Probability that a mosquito successfully lays eggs given that it has rested	0.88
Human blood index: the proportion of resting mosquitoes which fed on human blood during their last feed	0.5

These parameters have been previously estimated in (8) and were kept unchanged during all the simulations in the present study.

1.2.2 Calibration of the disease model and description of simulation experiments.

The present analysis is based on a previously calibrated version of the model that reflects demographic, epidemiology, entomology, health system, and seasonality of a health facility catchment area in Tanzania (12, 14, 34). Core calibration parameters were previously estimated using a genetic algorithm approach, with optimization of a weighted sum over 10 objective functions (25). These objective functions represent key epidemiological relationships captured from available study survey and study site data as follows (along with figures displaying data and model fits from the reference studies): age patterns of incidence after interventions (Fig. 5 from (26)), age patterns of prevalence (Fig. 4 in (10)), age patterns of parasite density (Fig. 6 in (10)), age patterns of the multiplicity of infections (Fig. 5 in (10)), age patterns of clinical malaria incidence (Fig. 1 in (13)), age patterns of the parasite density threshold for clinical attacks (Fig. 4 in (13)), hospitalization rate in relation to prevalence in children (Fig. 2 in (12)), age patterns of hospitalization in relation to severe malaria (Fig. 4 in (12)), malaria specific mortality in children less than 5-years-old (Fig. 7 and 8 in (12)), and indirect malaria infant mortality rate (Fig. 9 in (12)). Full calibration procedure details have been described in (25) and (16), and the estimated parameters have been previously summarized in Table 1 in (15), in Table 3 in (25), and in Additional file 1: Table S1 in (16). A summary of the model parameters has been provided in Additional file 1: Tables S1.2 and S1.3.

As summarized in the Methods section, the simulated human population size in this analysis was 10,000 individuals, with its age structure informed by data collected from a health and

demographic surveillance site in Ifakara, Tanzania, available through the INDEPTH network (30). For all simulations, it is assumed there were no imported infections during the entire study period.

Health system characteristics (Additional file 1: Table S1.1) were defined through parameterization of a case management model based on data provided by the Tanzanian National Malaria Control Program (14). To define the simulated case management level, the probability of effective cure within two weeks from the onset of fever (E_{14}) was varied within the interval [0-0.8] corresponding to a probability of seeking care (access to treatment) within 5 days from the onset of fever (E_5) within the interval [0.04-0.5] (21). During model simulations, the case management level was held constant over time.

Mosquito entomological parameters and seasonal exposure patterns were estimated from field studies conducted in the Namawala and Michenga villages located nearby Ifakara in Tanzania (35, 36). Two archetypal seasonal settings were simulated: a seasonal exposure setting with one transmission peak in September estimated from the mentioned field studies (Fig. S2.1), and a perennial setting with uniform, constant exposure throughout the year. Two mosquito species were present in the simulated settings: endophagic (indoor-biting, human blood index of 0.99) and exophagic (outdoor-biting, human blood index of 0.5), respectively. The ratios between the population sizes of indoor and outdoor mosquito species were classified into three levels corresponding to high (indoor proportion of 0.8 of total mosquito population), mid (indoor proportion of 0.5) and low biting (indoor proportion of 0.2). The extent of malaria transmission in each simulation was defined by the annual entomological inoculation rate (EIR). For each simulation, EIR was sampled from the interval [1-25] leading to a simulated range of *Plasmodium falciparum* parasite rate or prevalence (*Pf*PR) distributions across the various transmission settings (Additional file 1: Fig. S2.1 and S2.3, and Table S2.1).

1.2.3 Definition of intervention profiles.

As summarized in the Methods section, a standardized representation for each malaria intervention was built. Accordingly, a malaria intervention was characterized through the targets of the transmission life cycle it affects, along with the efficacy, half-life, and decay of its effect (Fig. 1, Additional file 1: Fig. S2.2, and Table 1). The efficacy of a therapeutic or immunologic intervention was quantified by its ability to clear parasites or to prevent infection, while for mosquito-targeted interventions (vector control) it corresponded to the ability of the intervention to kill or to prevent mosquitoes from biting humans. For each intervention, its efficacy decayed over time according to a specific decay type (defined in Fig. S2.2). Intervention coverage was quantified by the percentage of the population affected by the respective intervention. Where interventions were applied to individual humans they were equally applied across ages, and not targeted to certain populations. Geographical setting characteristics such as entomological inoculation rates (EIR), seasonality, case-management coverage, as well as transmission and vector characteristics were also included in the simulation specifications (Fig. 1, Table 1).

The following intervention targets were defined in the transmission cycle (Fig. 1): “anti-infective” as acting at the liver stage and preventing occurrence of a new infection, “blood stage clearance” as clearing blood-stage parasites by administration of a drug, “transmission blocking” as preventing parasite development into gametocytes, “mosquito life-cycle killing effect” as killing

mosquitoes during different stages of their life cycle, for example, before a blood meal (pre-prandial killing) and/or after a blood meal (post-prandial killing). Furthermore, mosquitoes are affected by vector control interventions according to their indoor and outdoor biting patterns.

The length of the intervention effect was described by half-life for exponential, sigmoidal, or biphasic decay profiles, or by duration for step-like decay profiles. Generally, half-life refers to the half-life of intervention efficacy decay, representing the time in which the initial intervention efficacy has been reduced by 50% (Additional file 1: Fig. S2.2, Table 1). The full duration of this effect is equivalent to the entire decay time. For simplicity, since only one intervention had a step-like decay, we used the words half-life and duration interchangeably.

Each intervention or combination of interventions was applied as mass intervention targeting to all ages equally, along with continuous case management. As part of development of this approach, targeting of particular populations or age groups was not examined. Mass intervention packages were deployed for a long model warm-up period (150 years) and were then implemented in June and/or December for three years (Fig. 3, Additional file 1: Fig. S3.1). Coverage at deployment time refers to the percentage of the population covered by the intervention's initial efficacy, irrespective of how many doses/applications are required to reach that coverage, assuming that the necessary doses have previously occurred. The base scenario XML files used for these simulations can be found at https://github.com/SwissTPH/TPP_workflow/tree/master/Intervention_scenarios.

1.2.4 Translation of input EIR to *Pf*$PR_{2\text{-}10}$ and *Pf*$PR_{0\text{-}99}$

For each simulation, OpenMalaria requires the definition of the intensity and seasonality of malaria exposure specified through the input EIR level and its yearly profile in the absence of interventions (Additional file 1: Fig. S2.1). EIR is an appropriate measure for reflecting transmission intensity (37), however it is difficult to measure in the field and its interpretation in the context of intervention impact is difficult to capture when looking at the effects of drugs and vaccines (38, 39). For this reason, although EIR is the force of infection input for all OpenMalaria simulations, simulation outcomes and downstream analyses at the corresponding median *Pf*$PR_{2\text{-}10}$ were reported before interventions were deployed. True infection prevalence was reported and not patent PCR or RDT-detected. To do so, the continuous EIR space was discretized into unit-wide intervals and the median *Pf*PR was calculated across the obtained *Pf*PR for all simulations at each discrete interval (Additional file 1: Fig. S2.3).

1.2.5 Definition of intervention impact and health goals

A comprehensive set of simulated scenarios was built by uniformly sampling the parameter space of setting and intervention characteristics. To estimate the impact of the deployed interventions, in each simulation, the reduction in *Pf*$PR_{0\text{-}99}$ attributable to the deployed intervention was calculated. *Pf*$PR_{0\text{-}99}$ reduction was calculated by comparing the initial average prevalence in the year before any interventions were deployed to the average annual prevalence obtained in the first (short follow-up) and third year (long follow-up) after interventions deployment (Fig. 3 and Additional file 1: Fig. S3.1). Consequently, the defined health goals corresponded to a given minimum threshold of *Pf*$PR_{0\text{-}99}$ reduction that the deployed interventions should achieve.

Additional file 1: Figures S3.2-S3.4 present the distributions of obtained *Pf*PR_{0-99} reduction for the OpenMalaria simulation experiments covering all the interventions and deployments investigated in the present study. In seasonal, low-transmission settings (EIR < 2) a high proportion of simulations reached elimination before any intervention was deployed and were removed from the analysis (Additional file 1: Fig. S3.5). Since this happened for over 75% of simulations at EIR < 2, we did not investigate optimal intervention profiles for transmission settings with EIR < 2. Arguably, for settings close to elimination, a different health goal, such as the probability of elimination, would be more appropriate which is outside the scope of this study, which focuses on reducing *Pf*PR_{0-99}.

2 Disease scenarios

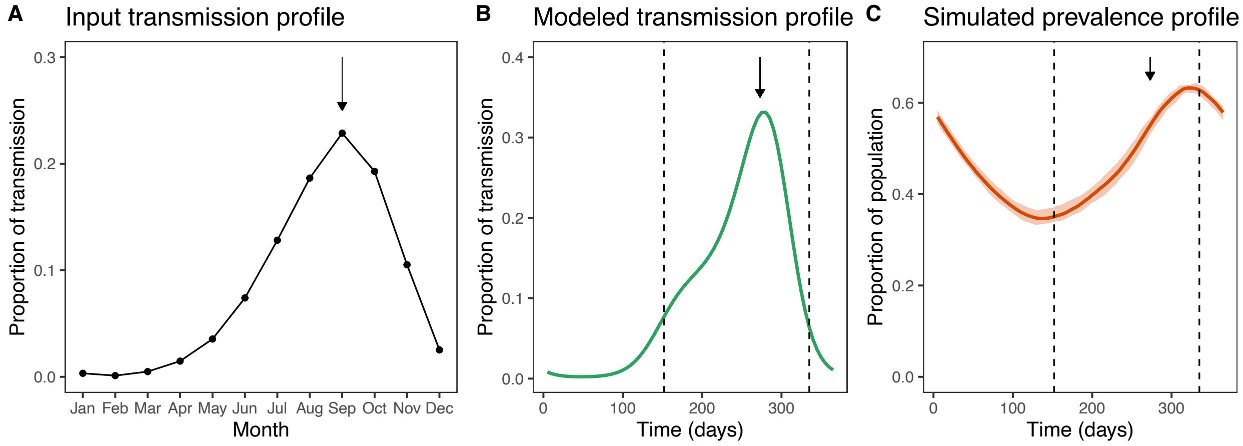

Fig. S2.1. Illustration of the yearly malaria transmission and prevalence patterns in simulated seasonal settings.

(A) Observed, normalized, monthly seasonal pattern of malaria EIR in Namawala, Tanzania extracted from (12). (B) Corresponding input, 5-day seasonal EIR pattern used in OpenMalaria simulations, obtained by scaling and extrapolating the monthly seasonality profile from (12) to 5-day time steps. For this example, the simulated input EIR was 7.78 infectious bites per person per year. (C) Resulting simulated yearly *Pf*PR_{0-99} profile. In all figures, the arrows indicate the month of September, the peak of transmission and show the delay between the peak of transmission and the resulting peak in malaria prevalence. The dotted vertical lines on figures (B) and (C) indicate the deployment times of first and second rounds of malaria interventions when applicable.

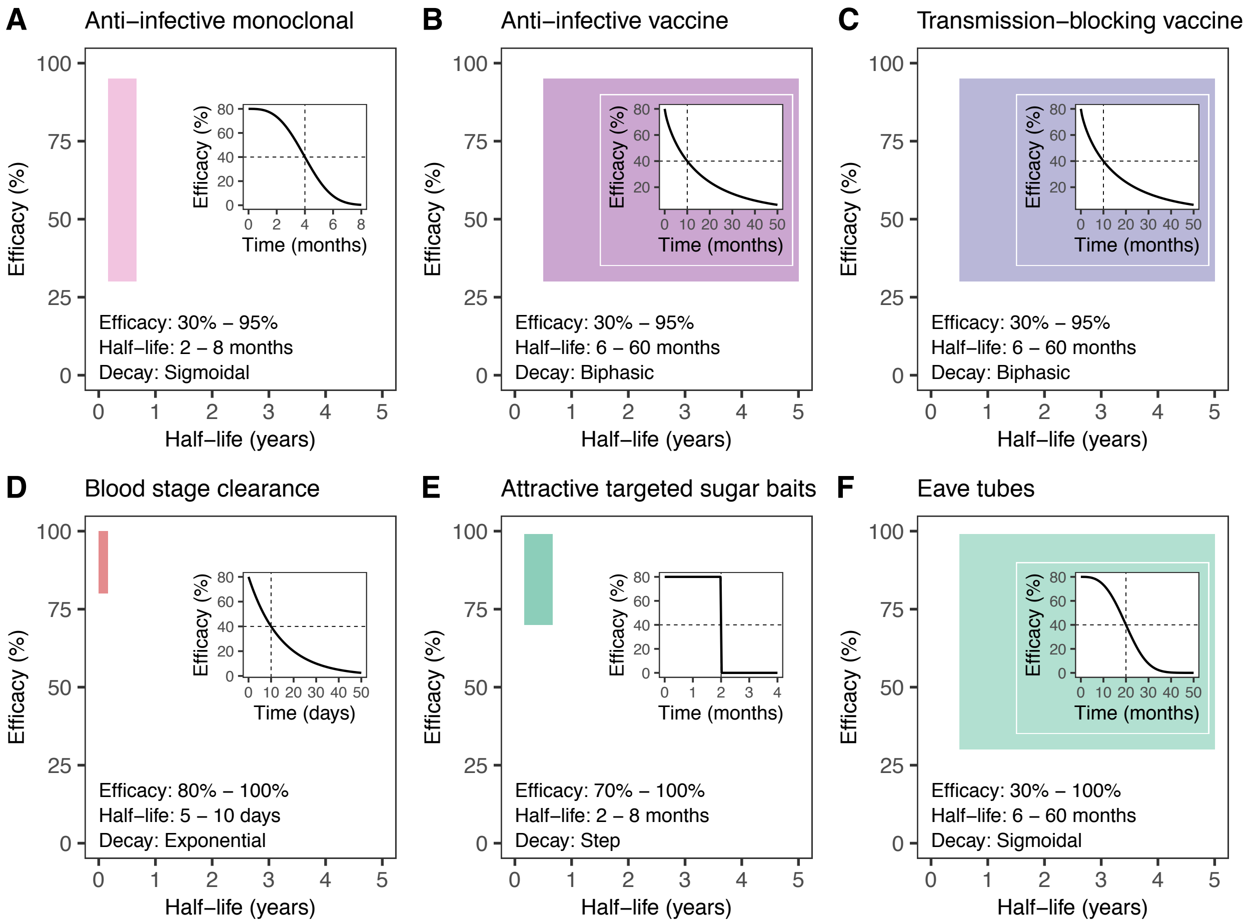

Fig. S2.2. Representation of decay and the range of efficacy and half-life against different parasite or vector targets for intervention-agnostic malaria interventions.

The simulated malaria interventions (A–F) were modeled in terms of their targets in the malaria transmission cycle. The effect of each intervention is represented through the half-life of its decay (x-axis) as well as the initial efficacy (y-axis). The color blocks represent the range of parameter space of efficacy and half-life of decay considered in the current analysis for each intervention. The half-life and the color block do not represent the entire duration of effect, as that depends on the decay shape chosen for each intervention. The decay shape for each intervention is displayed in the right side insert of each plot where the dotted lines specify the half-life and corresponding half of the intervention efficacy. The definitions of all the parameter ranges for all interventions are provided in each figure on the lower left side and detailed in Table S2.1.

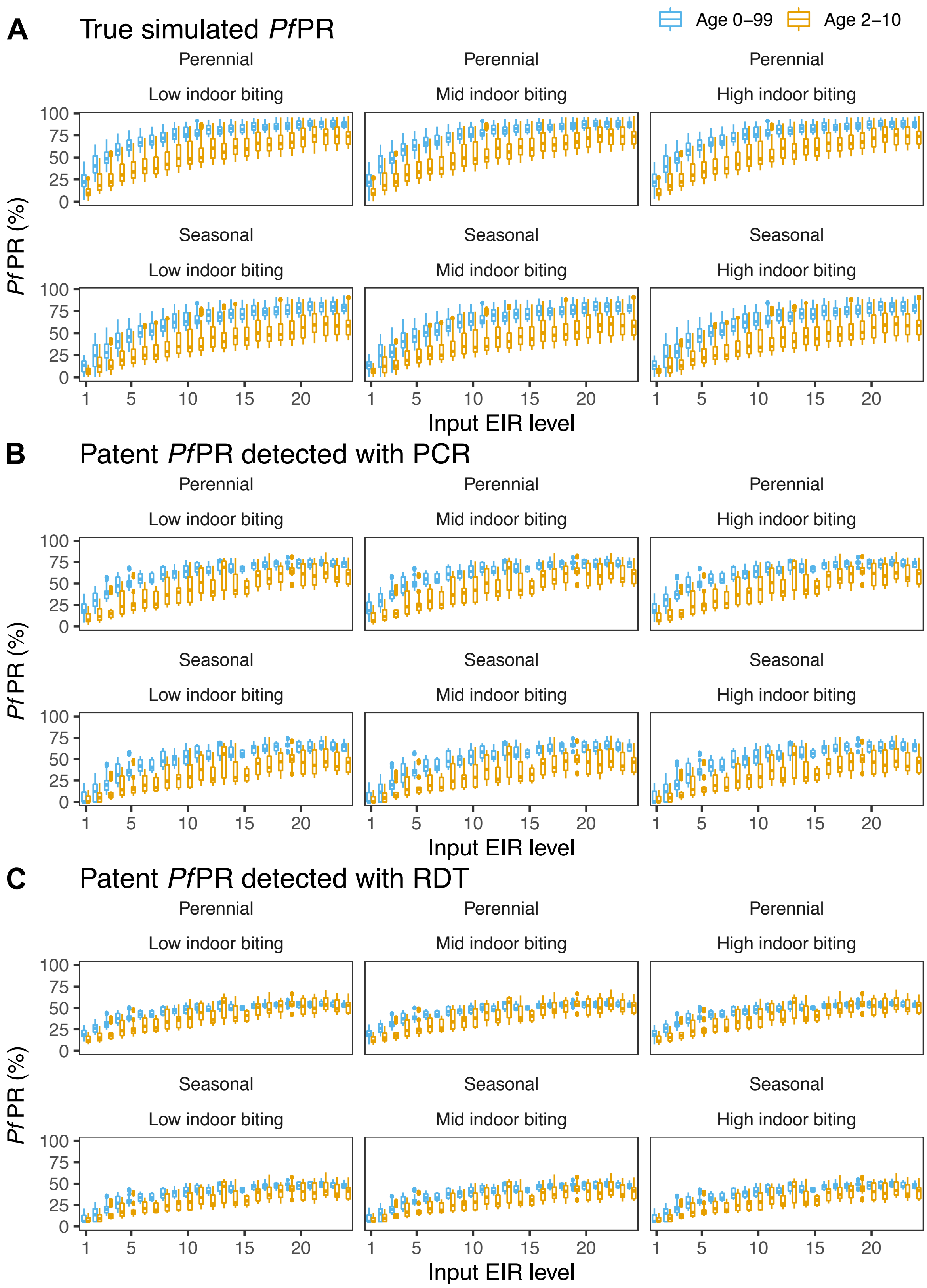
A
True simulated PfPR
Age 0–99
Age 2–10
Perennial
Low indoor biting
Mid indoor biting
High indoor biting
Seasonal
PfPR (%)
Input EIR level
B
Patent PfPR detected with PCR
C
Patent PfPR detected with RDT

Fig. S2.3. Simulated distributions of true and patent (detected with PCR or RDT) *Pf*PR$_{0\text{-}99}$ and *Pf*PR$_{2\text{-}10}$ for various input EIR levels in absence of interventions.

The input entomological inoculation rate (EIR) defines the simulated malaria transmission level. In every simulation experiment, EIR was uniformly sampled from the interval [1, 25]. In figures (A)–(C), each panel corresponds to a simulated setting and presents the distributions of true (A), patent with PCR (B) and patent with RDT (C) *Plasmodium falciparum* prevalence (*Pf*PR, shown with boxplots, blue for 0-99-year-old and orange for 2-10-year-old) at varying EIR levels (x-axis). The 6 represented settings are defined by the seasonality pattern (perennial shown in the first row, or seasonal shown in the second row of each figure) and mosquito indoor biting behavior (low- shown in the first column, mid- shown in the second column or high-indoor biting shown in the third column of each figure). Each EIR level on the x-axis is defined as a set of continuous input EIR values which range between the current level and the current level-1, e.g., an input EIR level of 1 contains EIR values in the interval (0, 1]. For each EIR level and setting, the case management levels, i.e., the probability of seeking care (access to treatment) within 5 days from the onset of fever (E_5), was varied within the interval [0.04-0.5]. PCR: polymerase chain reaction. RDT: rapid diagnostic test.

Table S2.1. Specifications of the optimization procedure for TPP development

Intervention	Minimized profile	Intervention properties constraints	Specifications of combination therapies
Anti-infective monoclonal antibody (Sigmoidal decay)	Coverage	Coverage ∈ [0–80%]<br>Efficacy = 85%<br>Half-life = 4 months	Blood stage drug:<br>Efficacy = 90%<br>Half-life = 10 days
	Efficacy	Coverage = 60%<br>Efficacy ∈ [30%–95%]<br>Half-life = 4 months	
	Half-life	Coverage = 60%<br>Efficacy = 85%<br>Half-life ∈ [2–8 months]	
Anti-infective vaccine (Biphasic decay)	Coverage	Coverage ∈ [0–80%]<br>Efficacy = 85%<br>Half-life = 7 months	Blood stage drug:<br>Efficacy = 90%<br>Half-life = 10 days
	Efficacy	Coverage = 60%<br>Efficacy ∈ [30%–95%]<br>Half-life = 7 months	
	Half-life	Coverage = 60%<br>Efficacy = 85%<br>Half-life ∈ [2 months–5 years]	
Transmission-blocking vaccine (Biphasic decay)	Coverage	Coverage ∈ [0–80%]<br>Efficacy = 85%<br>Half-life = 7 months	Blood stage drug:<br>Efficacy = 90%<br>Half-life = 10 days
	Efficacy	Coverage = 60%<br>Efficacy ∈ [30%–95%]<br>Half-life = 7 months	
	Half-life	Coverage = 60%<br>Efficacy = 85%<br>Half-life ∈ [2 months–5 years]	
Attractive targeted sugar baits (Step decay)	Coverage	Coverage ∈ [0–80%]<br>Efficacy = 85%<br>Half-life = 4 months	Not applicable
	Efficacy	Coverage = 60%<br>Efficacy ∈ [70%–99%]<br>Half-life = 4 months	
	Half-life	Coverage = 60%<br>Efficacy = 85%<br>Half-life ∈ [2–8 months]	
Eave tubes (Sigmoidal decay)	Coverage	Coverage ∈ [0–80%]<br>Efficacy = 85%<br>Half-life = 3 years	Not applicable
	Efficacy	Coverage = 60%<br>Efficacy ∈ [30–99%]<br>Half-life = 3 years	
	Half-life	Coverage = 60%<br>Efficacy = 85%<br>Half-life ∈ [6 months–5 years]	

For each intervention, we successively identified the minimum profiles of the intervention coverage, efficacy, and half-life. Precisely, we optimized each parameter separately (column "Minimized profile"), according to its feasibility constraints while setting the two other parameters to the specified fixed values (column "Intervention properties constraints"). When

deployed in combination with other drugs or vaccines, the additional interventions had fixed properties as well (column "Specifications of combination therapies").

2.1 Building a disease model emulator with Gaussian processes

As stated in the Methods, since it was computationally intensive and challenging to run an exhaustive number of simulations in order to explore the entire parameter space for diverse combinations of interventions, settings, and deployments using OpenMalaria, machine learning techniques and kernel methods were applied. Precisely, starting from a training dataset of simulations generated with OpenMalaria, Gaussian process (GP) models (40) were used to infer the relationship between simulation variables (e.g., intervention coverage, half-life, efficacy, etc.) and corresponding intervention impact (*Pf*PR$_{0\text{-}99}$ reduction). This approach allowed a fast, simplified predictive model that could provide estimates of the disease model output for any new inputs to be built without running new OpenMalaria model simulations.

Gaussian process models are non-parametric models which define a prior probability distribution over a collection of functions using a kernel, smoothing function. Precisely, given the relationship

$$y = f(\boldsymbol{x}) + \varepsilon$$

where y is the *Pf*PR$_{0\text{-}99}$ reduction here, and $\boldsymbol{x}$ represents the set of intervention parameters $x_1, \ldots, x_n$, the main assumption of a GP is that

$$P\big(f(x_1), f(x_2), \ldots, f(x_n)\big) \sim N(\mu, \Sigma)$$

where

$$\Sigma_{x_i,x_j} = K\big(x_i, x_j\big)$$

is the covariance matrix of the Gaussian distribution, μ is its mean, and K is a kernel function (40). Once data are observed, the posterior probability distribution of the functions consistent with the observed data can be derived, which is then used to infer outcomes at unobserved locations in the parameter space (40). The intuition behind a GP model is based on the "smoothness" relationship between its components. Accordingly, points which are close in the input parameter space will lead to close points in the output space.

2.2 Training data

For each intervention and setting, a training dataset was built using discrete Latin hypercube uniform sampling (41) across the input parameter space (defined in Table 1). This sampling scheme ensured uniform coverage of the parameter space and a representative set of points spanning the variability of the predicted output across the entire space. Ten stochastic realizations (replicates) of each sampled data point were considered. OpenMalaria was run on the sampled data and *Pf*PR$_{0\text{-}99}$ was calculated for short and long follow-up. The size of the training set was varied between 10 and 1,000 points (100-10,000 including replicates) for several simulation experiments (Additional file 1: Fig. S4.1) and the performance of the trained GP was assessed via the Pearson correlation coefficient r^2. The minimum training set size which led to $r^2 > 0.95$ was selected for the remaining simulation experiments.

2.3 Gaussian process emulators

For each transmission setting and intervention, a GP model with a Gaussian kernel was trained for a 5-fold cross-validation scheme using the training dataset with OpenMalaria simulations. For training the GP, the R package HetGP version 1.1.1 was used (42, 43). HetGP is a powerful implementation of GP models, featuring heteroskedastic GP modeling embedded in a fast and efficient maximum-likelihood-based inference scheme.

GP performance was assessed by calculating the correlation between true and predicted outputs on out-of-sample test sets, as well as the mean squared error (Additional file 1: Fig. S4.2 and S4.3 and Table S4.1). Precisely, the training set was split into 5 subsets and, iteratively, 4 of these subsets were used for training the GP, while the remaining set was used as an out-of-sample test set during the cross-validation procedure. After assessing the prediction error obtained during the cross-validation procedure, the GP was trained using the entire training set.

Furthermore, since the trained GP model provides the mean and variance for each predicted output, this probabilistic representation was used to assess the uncertainty of the trained model across the entire parameter space and to refine the GP model through adaptive sampling (44-46). Accordingly, new training points from high-uncertainty regions of the parameter space were iteratively sampled and the model was updated with the new training samples. Precisely, 100,000 points were sampled using Latin hypercube sampling and the variance of the predicted output was evaluated with the previously trained GP emulator. Samples from "rare" regions of the parameter space containing fewer outputs were prioritized. To do so, the output *Pf*PR_{0-99} range was classified into 4 bins and the numbers of predicted outcomes corresponding to the 100,000 sampled points that fell in each bin (output density) calculated. Of the sampled points, 30 points (300 including replicates) were chosen proportional to the output density in each bin and with the highest predicted variance. This procedure was repeated five times to ensure that the correlation between true and predicted values on an out-of-sample test set had reached a plateau. Finally, a separate out-of-sample test set was built to assess the overall performance of the GP (Additional file 1: Fig. S4.2, S4.3, and Table S4.1).

3 Results: Disease model simulation

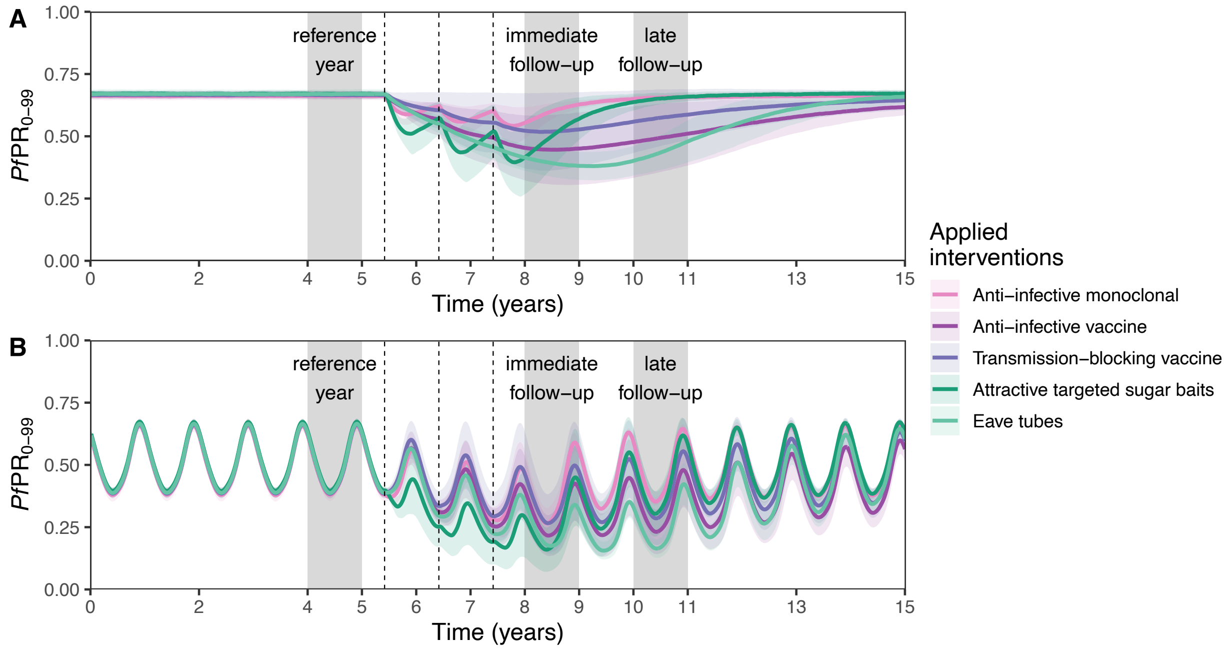

Fig. S3.1. Examples of OpenMalaria simulation outputs.
Time series of simulated malaria *Pf*PR$_{0\text{-}99}$ in a perennial (A) and seasonal (B) setting. Both figures display the prevalence of malaria cases, *Pf*PR$_{0\text{-}99}$, (y-axis) across time (x-axis). Interventions targeting different stages in the malaria transmission cycle (different colors) are applied once per year at the beginning of June (vertical dotted lines, in this example for three years of deployment). The effect of each intervention is assessed by evaluating the *Pf*PR$_{0\text{-}99}$ reduction in all ages relative to the year prior deployment (first grey block). Two outcomes are assessed, following an immediate and late follow-up (second and third grey blocks), depending on whether the average prevalence is calculated across the next year after deployment, or across the third year after deployment, respectively.

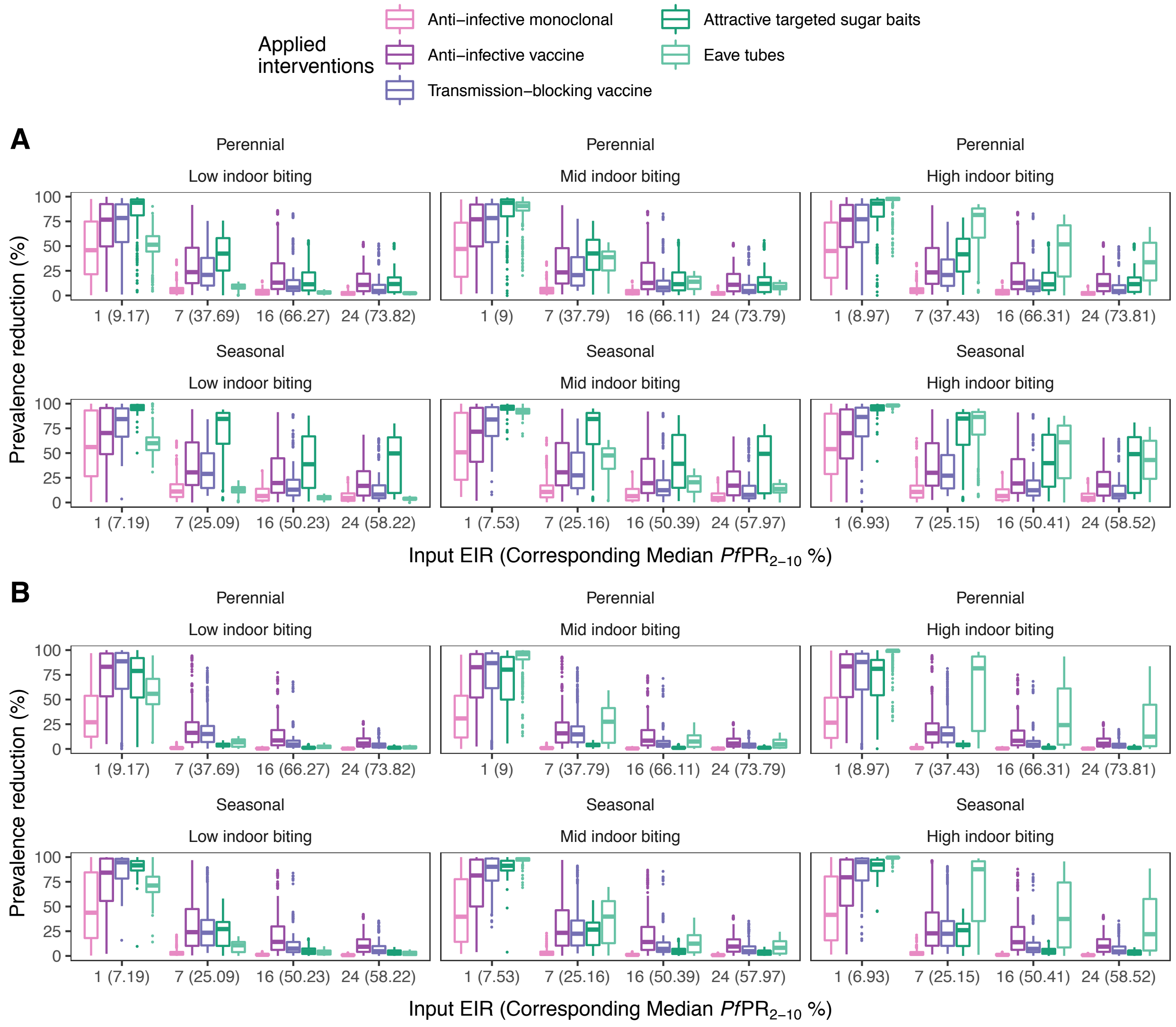

Fig. S3.2. Distributions of prevalence reduction following yearly deployment of single interventions.

Prevalence reduction was calculated by comparing the initial prevalence in the year before any interventions were deployed to the yearly prevalence obtained in the following year (short follow-up, panel A) and in the third year (long follow-up, panel B) after deployment of interventions. Each individual figure corresponds to a simulated setting and presents the distributions of *Pf*PR_{0-99} reduction (shown with boxplots) at varying EIR as well as corresponding simulated *Pf*PR_{2-10} levels (x-axis). Each boxplot displays the interquartile range (box), the median value (horizontal line), the largest and smallest values within 1.5 times the interquartile range (whiskers), and the remaining outside values (points). The 6 represented settings in each panel are defined by the seasonality pattern (perennial or seasonal) and mosquito indoor biting behavior (low, mid, or high indoor biting). Each EIR level on the x-axis is defined as a set of continuous input EIR values which range between the current level and the current level-1, e.g., an input EIR level of 1 contains all EIR values in the interval (0, 1]. The definitions and ranges of all the EIR levels is included in Table S2.1.

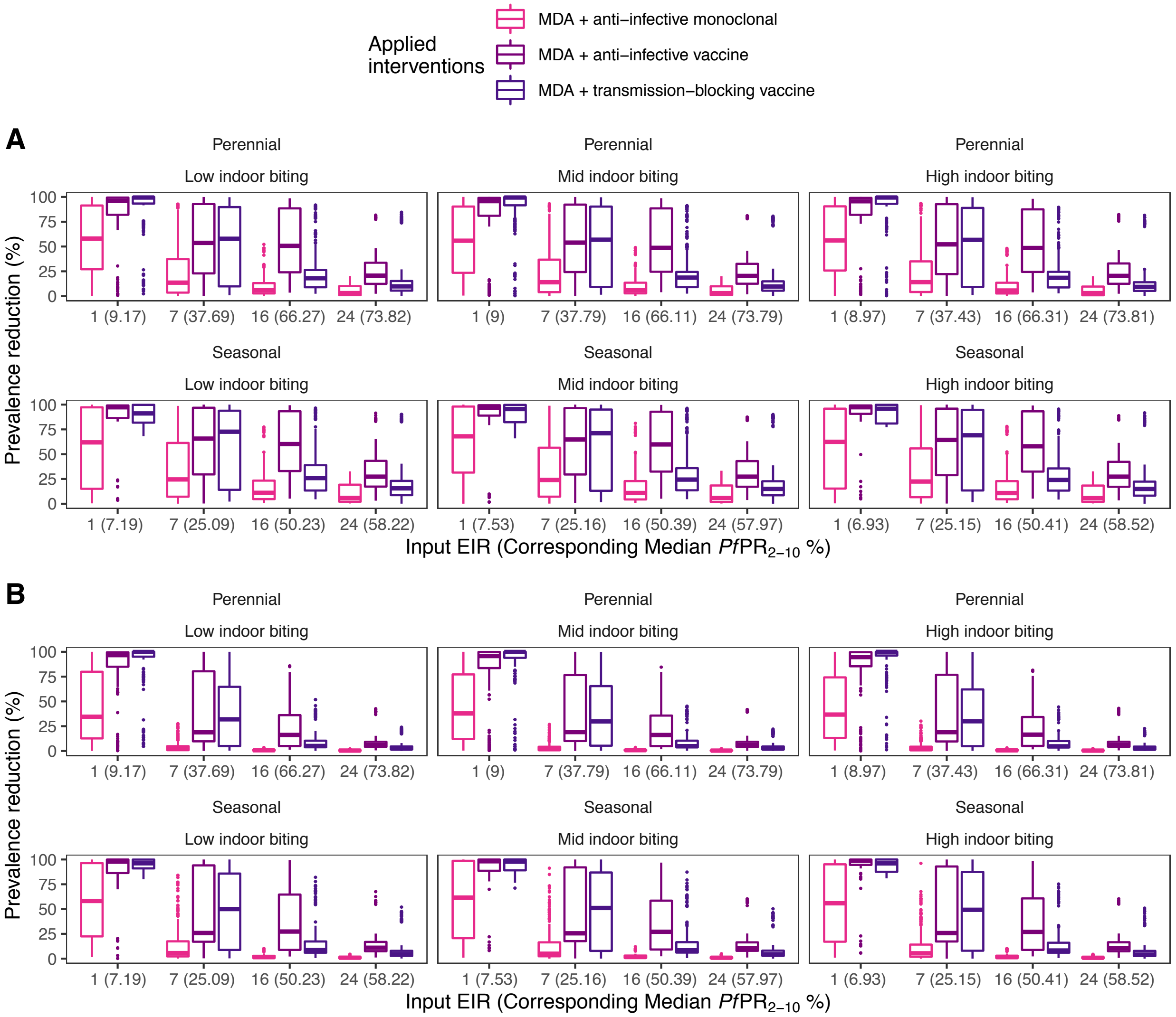

Fig. S3.3. Distributions of prevalence reduction following yearly deployment of combinations of interventions.

Prevalence reduction was calculated by comparing the initial prevalence in the year before any interventions were deployed to the yearly prevalence obtained in the following year (short follow-up, panel A) and in the third year (long follow-up, panel B) after deployment of interventions. Each individual figure corresponds to a simulated setting and presents the distributions of *Pf*$PR_{0\text{-}99}$ reduction (shown with boxplots) at varying EIR as well as the corresponding simulated *Pf*$PR_{2\text{-}10}$ levels (x-axis). Each boxplot displays the interquartile range (box), the median value (horizontal line), the largest and smallest values within 1.5 times the interquartile range (whiskers), and the remaining outside values (points). The 6 represented settings in each panel are defined by the seasonality pattern (perennial or seasonal) and mosquito indoor biting behavior (low, mid, or high indoor biting). Each EIR level on the x-axis is defined as a set of continuous input EIR values which range between the current level and the current

level-1, e.g., an input EIR level of 1 contains EIR values in the interval (0, 1]. The definitions and ranges of all the EIR levels are included in Table S2.1. MDA: mass drug administration.

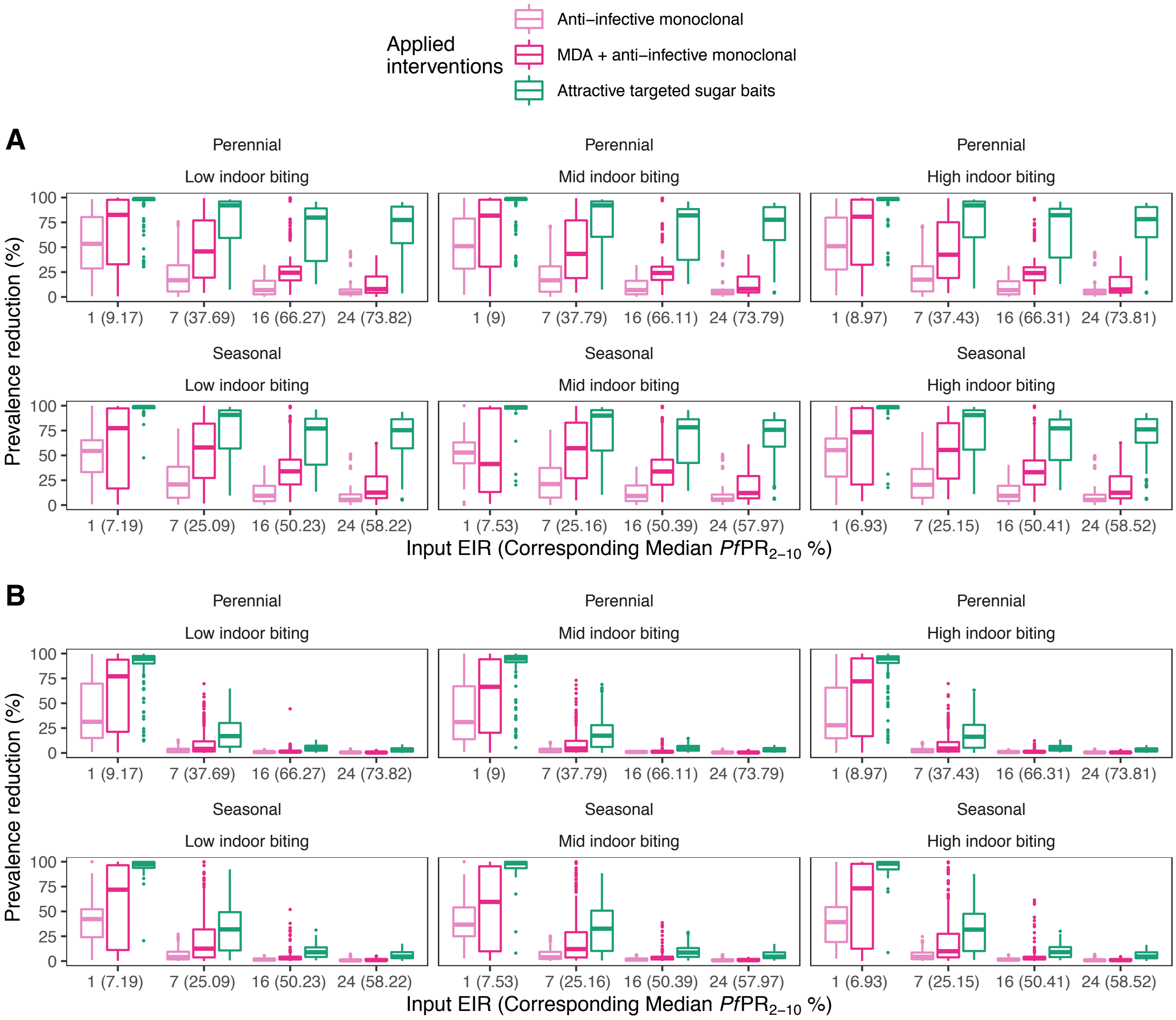

Fig. S3.4. Distributions of prevalence reduction following deployment of single and combinations of interventions twice per year.

Prevalence reduction was calculated by comparing the initial prevalence in the year before any interventions were deployed to the yearly prevalence obtained in the following year (short follow-up, panel A) and in the third year (long follow-up, panel B) after deployment of interventions. Each individual figure corresponds to a simulated setting and presents the distributions of *Pf*$PR_{0\text{-}99}$ reduction (shown with boxplots) at varying EIR as well as corresponding simulated *Pf*$PR_{2\text{-}10}$ levels (x-axis). Each boxplot displays the interquartile range (box), the median value (horizontal line), the largest and smallest values within 1.5 times the interquartile range (whiskers), and the remaining outside values (points). The 6 represented settings in each panel are defined by the seasonality pattern (perennial or seasonal) and mosquito indoor biting behavior (low, mid, or high indoor biting). Each EIR level on the x-axis is defined

as a set of continuous input EIR values which range between the current level and the current level-1, e.g., an input EIR level of 1 contains EIR values in the interval (0, 1]. The definitions and ranges of all the EIR levels for all simulated settings is included in Table S2.1. MDA: mass drug administration.

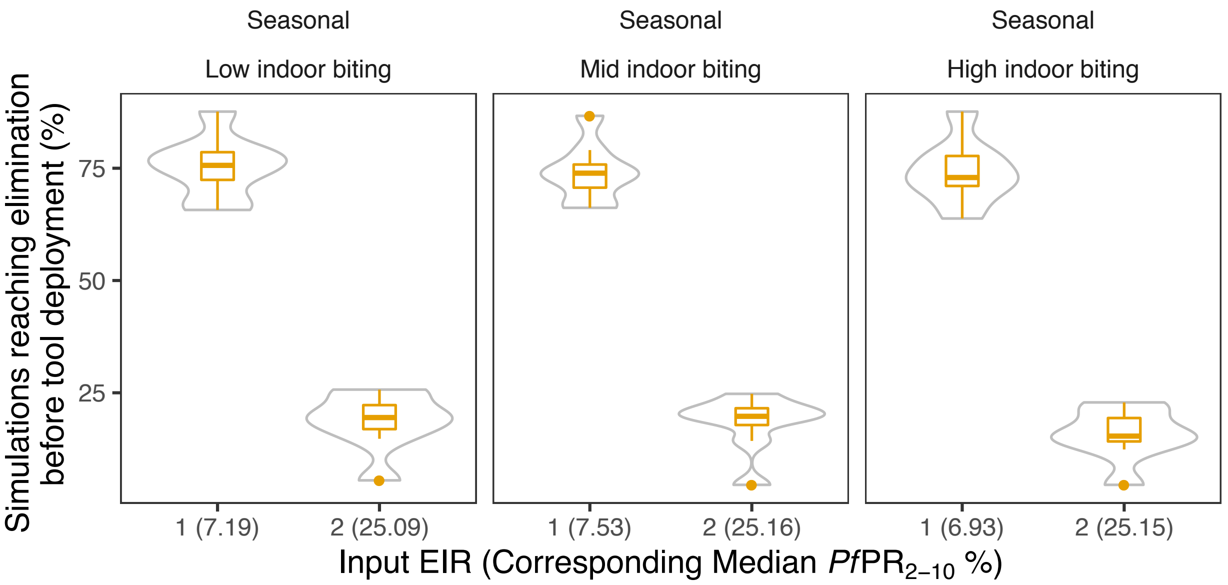

Fig. S3.5. Simulations reaching malaria elimination before intervention deployment. The violin plots and boxplots in each panel present the distributions of the percentage of simulations reaching malaria elimination ($Pf\mathrm{PR}_{0\text{-}99} = 0$) before intervention deployment (this can arrive due to case management and only occurs in seasonal settings), across all simulated interventions and intervention combinations.

4 Sensitivity analysis and emulator performance results

4.1 Identifying impact determinants through sensitivity analysis

To estimate the contribution of each model input and its interactions with the other inputs to the variance of the model outcome, a global sensitivity analysis based on variance decomposition (47) was conducted. This analysis shows which input parameters have higher impact on the model outcome. It relies on the decomposition of the output variance in a sum of individual input parameter conditional variances:

$$\mathrm{Var}(Y) = \sum_{i} V_i + \sum_{i}\sum_{j>i} V_{ij} + \cdots + V_{12\ldots d}$$

where Y is the model outcome (in this case, $Pf\mathrm{PR}_{0\text{-}99}$ reduction), d is the number of model inputs, and the conditional variances defined as:

$$V_i = \mathrm{Var}(E(Y|x_i))$$

$$V_{ij} = \mathrm{Var}\left(E\left(Y|x_i, x_j\right)\right) - V_i - V_j$$

$$V_{ijk} = \mathrm{Var}\left(E\left(Y|x_i, x_j, x_k\right)\right) - V_{ij} - V_{jk} - V_{ik} - V_i - V_j - V_k$$

$$\ldots$$

with $x_1, \ldots, x_n$ representing the model input parameters.

Based on the above decomposition of output variance, the first order sensitivity index is defined as:

$$S_i = \frac{V_i}{\mathrm{Var}(Y)}$$

and corresponds to the proportion of output variance assigned to the main effect of X_i, i.e., regardless of its interactions with other model inputs (47, 48).

To account for the contribution of each model input, as well as the variance of its interactions with other inputs to the variability of the model output, the total effect sensitivity index is used:

$$T_i = 1 - \frac{\mathrm{Var}(E(Y|x_{\sim i}))}{\mathrm{Var}(Y)}$$

where $\sim i$ stands for all indices except i (47, 48).

In the above decomposition of model output variance, by replacing the expressions of the sensitivity indexes, the following properties can be deduced:

$$\sum_{i} S_i + \sum_{i}\sum_{j>i} S_{ij} + \cdots + S_{12\ldots d} = 1$$

and

$$\sum_{i} T_i \geq 1\,.$$

To compute the sensitivity indexes, the function *soboljansen* from the R package *sensitivity* (49) was used. The function estimates the sensitivity indices through MCMC sampling, using a Monte Carlo approximation for computing conditional expectations. Within the sampling scheme, 100,000 points to estimate the sensitivity indices were sampled.

Calculating the sensitivity indices defined above, the variance of the GP emulator output was thus decomposed into proportions attributable to intervention characteristics, i.e., intervention efficacy,

half-life, and deployment coverage, as well as access to care. Using the main effects, the relative importance r_i of each characteristic as a proxy for impact determinants was defined as follows:

$$r_i = \frac{S_i}{\sum_{i=1}^{d} Si}$$

where d is the number of intervention characteristics and $\sum_{i=1}^{d} r_i = 1$.

4.2 Results: Sensitivity analysis and emulator performance

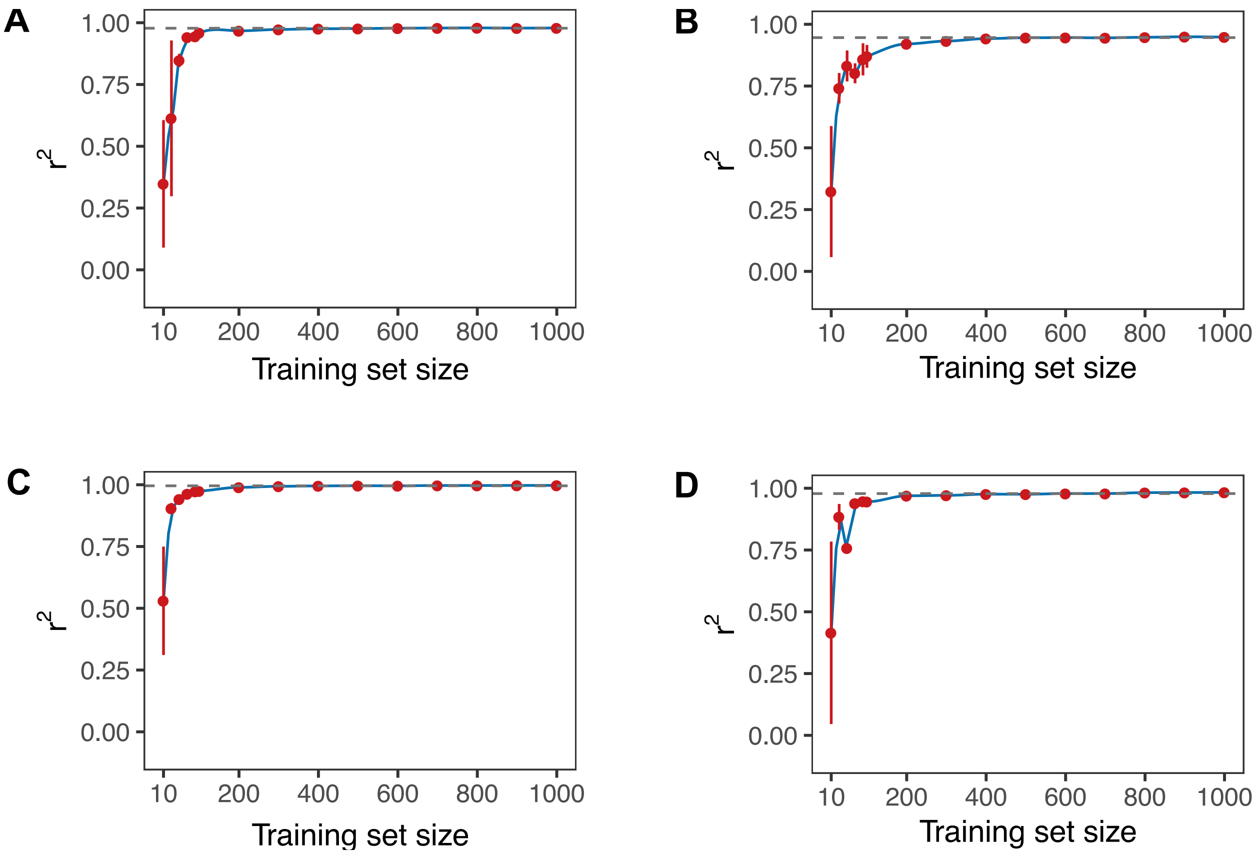

Fig. S4.1. Assessment of the performance of the trained GP depending on the training set size.

Each figure presents the Pearson correlation coefficient r^2 between true and predicted values on a broad range of out-of-sample test sets of varying length, when simulating deployment of an anti-infective monoclonal antibody deployed once per year (A) or twice per year (B) as well as in combination with a blood-stage drug once (C) or twice per year (D).

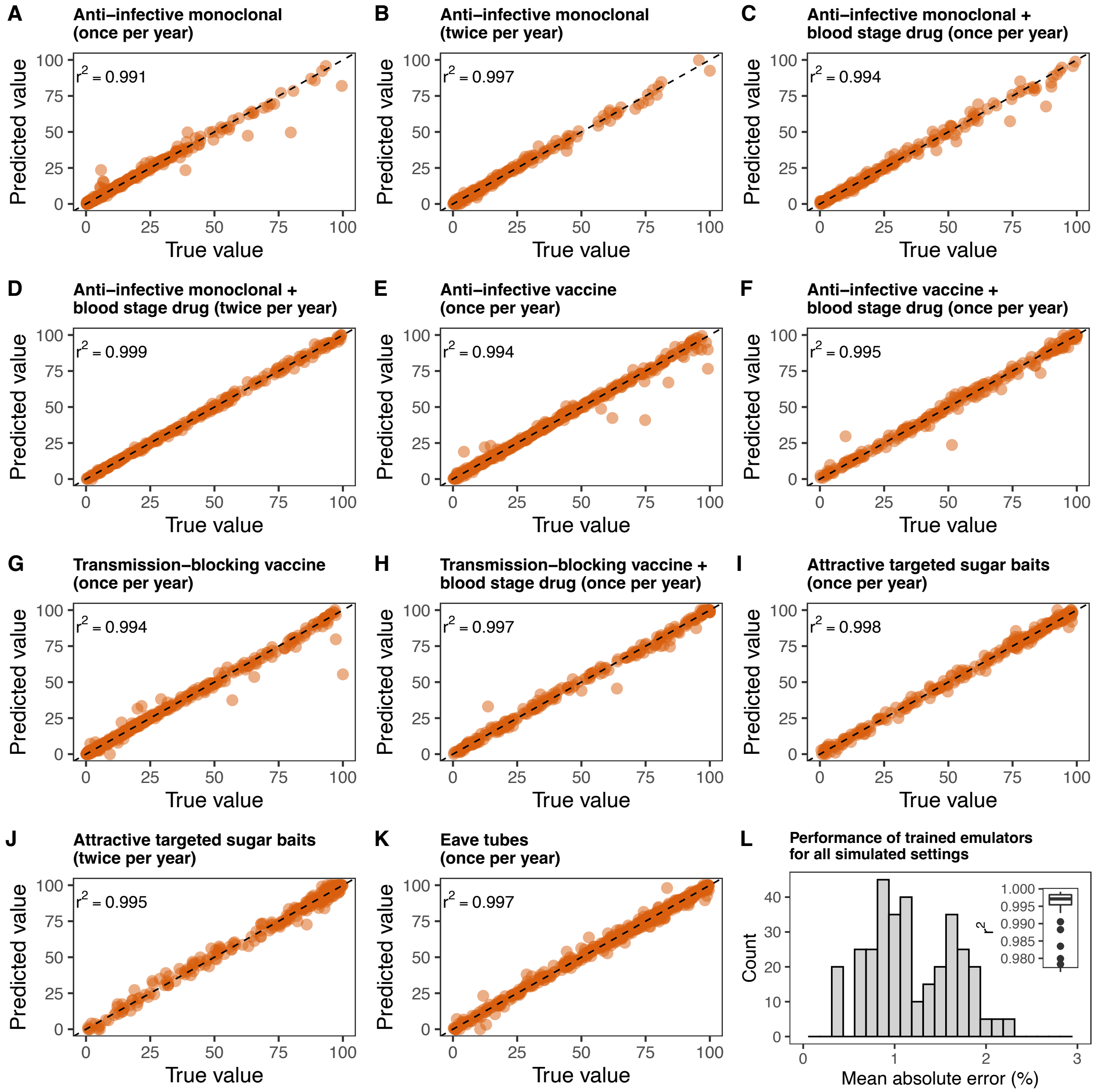

Fig. S4.2. Performance of the trained GP emulators predicting immediate intervention impact.

For a wide range of deployed interventions and transmission settings (see Methods), GP emulators were trained to predict the immediate impact of each intervention, i.e., the resulting average *Pf*PR$_{0\text{-}99}$ reduction in the year following deployment of the intervention. The performance of the trained emulators was assessed by inspecting the Pearson correlation coefficient (r^2) and the mean absolute error between true and predicted values on an out-of-sample test set. Figures (A) – (K) display the true and predicted values of each trained emulator across all deployed interventions in a seasonal transmission setting with high indoor biting. Figure (L) summarizes r^2 and the mean absolute error of all the trained emulators for all simulated transmission settings and interventions (the simulated settings were defined by seasonality and mosquito biting patterns, see Table S2.1 for detailed values per setting).

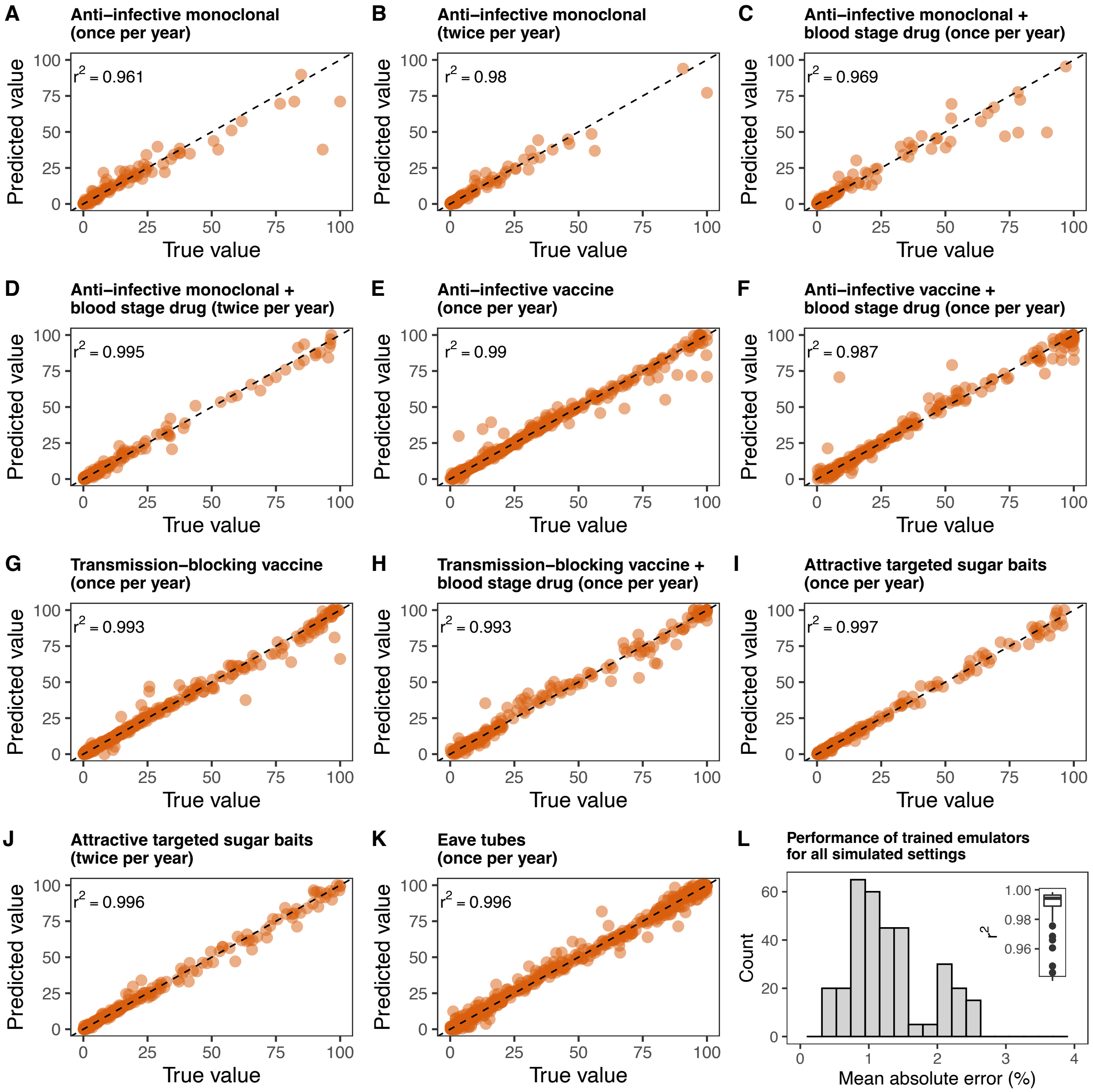

Fig. S4.3. Performance of the trained GP emulators predicting long-term intervention impact.

For a wide range of deployed interventions and transmission settings (see Methods section), GP emulators were trained to predict the immediate impact of each intervention, i.e., the resulting average $Pf\text{PR}_{0\text{-}99}$ reduction in the third year following deployment of the intervention. The performance of the trained emulators was assessed by inspecting the Pearson correlation coefficient (r^2) and the mean absolute error between true and predicted values on an out-of-sample test set. Figures (A) – (K) display the true and predicted values of each trained emulator across all deployed interventions in a seasonal transmission setting with high indoor biting. Figure (L) summarizes r^2 and the mean absolute error of all the trained emulators for all simulated transmission settings and interventions (the simulated settings were defined by seasonality and mosquito biting patterns, see Table S2.1 for detailed values per setting).

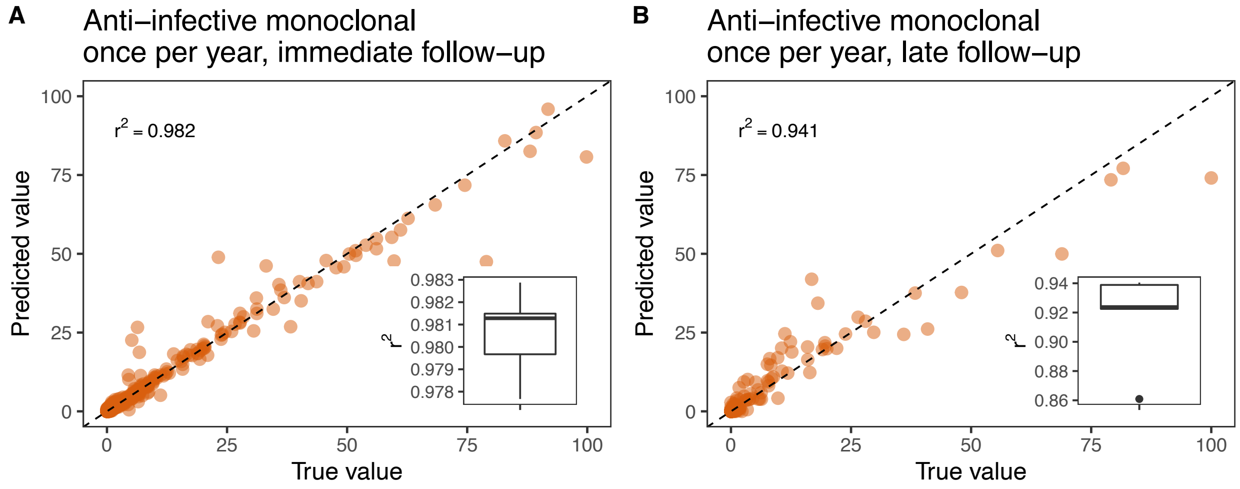

Fig. S4.4. Performance of trained GP emulators predicting incidence reduction. *Plasmodium falciparum* malaria incidence reduction in all ages in the first (A) and third (B) year following deployment of a monoclonal antibody intervention. The performance of the trained emulators was assessed by inspecting the Pearson correlation coefficient (r^2) in a cross-validation scheme (lower right boxplot displays the distribution of r^2 obtained on the left-out test sets during cross-validation) as well as on an out-of-sample test set (upper left corner).

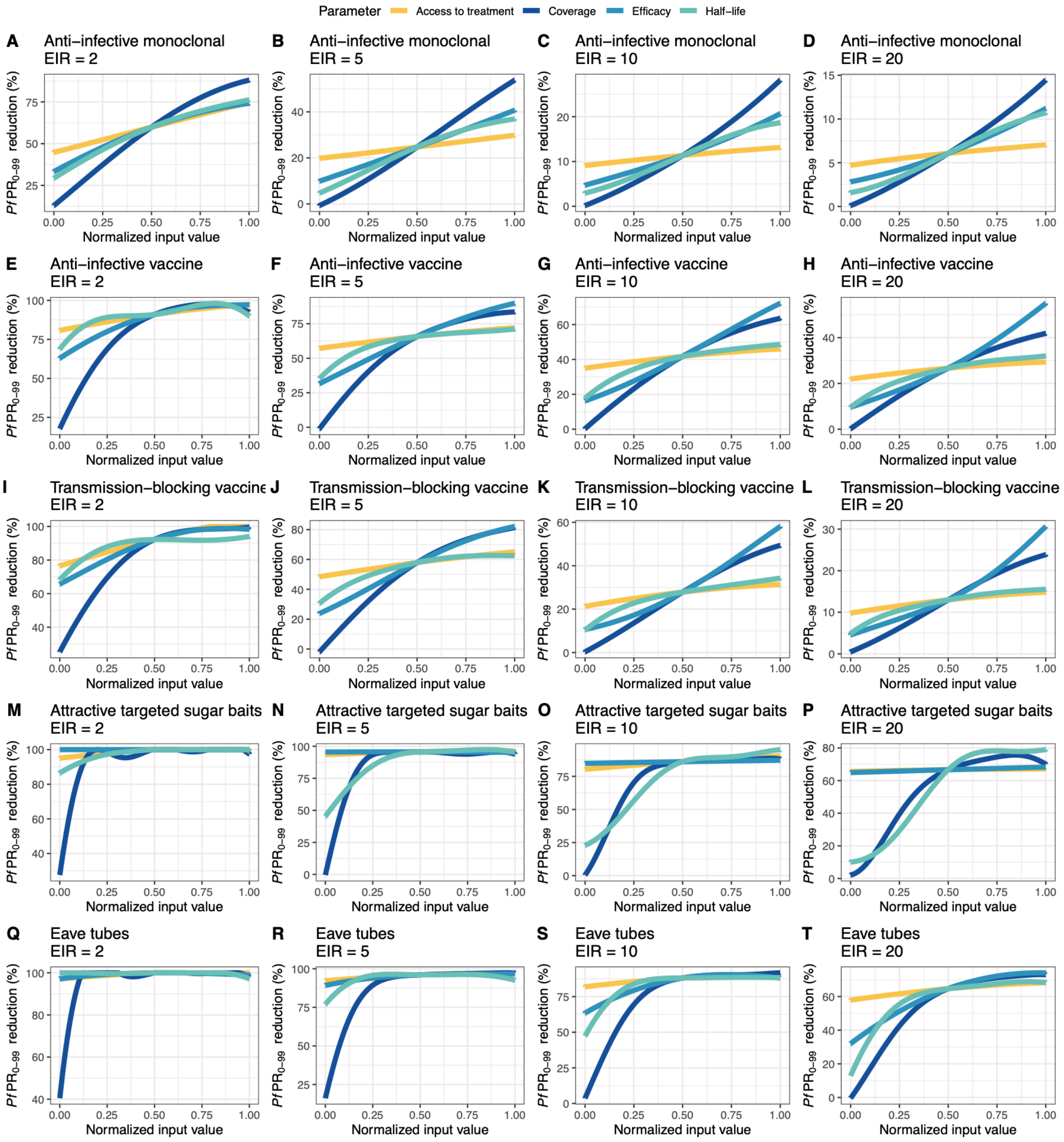

Fig. S4.5. Relationships between input intervention parameters and the predicted immediate *Pf*PR$_{0\text{-}99}$ reduction with the trained GP emulator.

Each parameter (intervention characteristic) was varied in turn across its defined range (parameter ranges are defined in Table 1) while the remaining parameters were set to their average values. The figures display the immediate *Pf*PR$_{0\text{-}99}$ reduction predicted with the GP emulator. A min-max normalization was used to display the varying input values of each of the parameters.

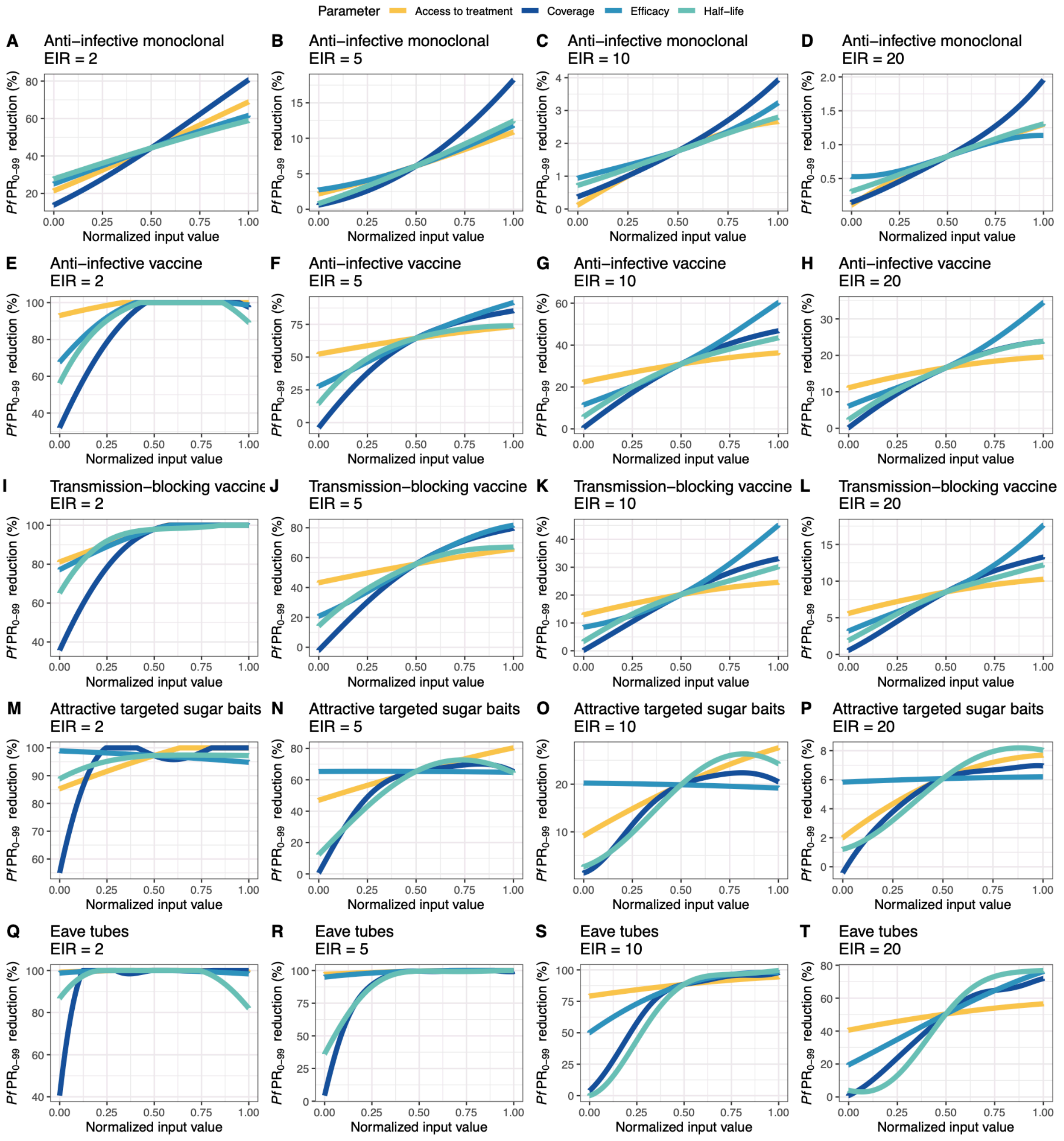

Fig. S4.6. Relationships between input intervention parameters and the predicted long-term *Pf*PR$_{0\text{-}99}$ reduction with the trained GP emulator.

Each parameter (intervention characteristic) was varied in turn across its defined range (parameter ranges are defined in Table 1) while the remaining parameters were set to their average values. The figures display the long-term *Pf*PR$_{0\text{-}99}$ reduction predicted with the GP emulator. A min-max normalization was used to display the varying input values of each of the parameters.

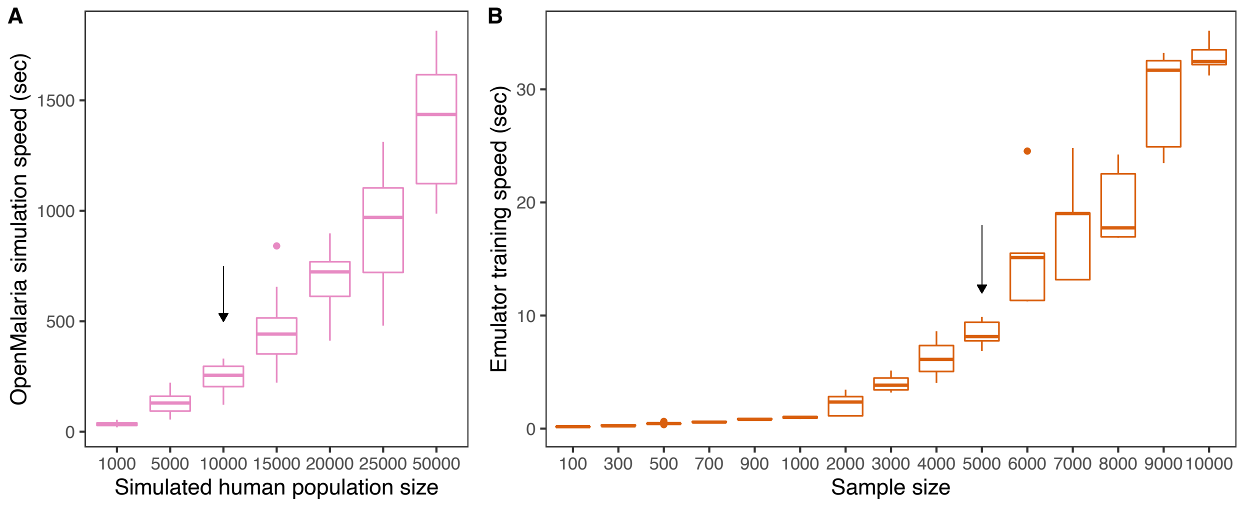

Fig. S4.7. Execution time of OpenMalaria simulations and GP emulator training. (A) CPU execution time of a single OpenMalaria simulation for varying population size. The arrow indicates the population size of 10,000 human hosts used in the present study. (B) CPU time required for training the GP emulator using training sets of OpenMalaria simulations of varying size. The arrow indicates the typical sample size used in the present analysis. In both figures, execution times were estimated 5 times in each case and the resulting distribution was displayed (boxplots).

Table S4.1. Performance of the trained GP emulators predicting immediate and long-term intervention impact

Intervention(s) (deployment)	Training set size	Test set size	Cross-validation r^2 and (mean error)	Test set r^2 and (mean error)
Anti-infective monoclonal antibody (once/year)	10000	5000	**Immediate:** 0.99 (1.02%)<br>**Long:** 0.96 (1.15%)	**Immediate:** 0.99 (0.63%)<br>**Long:** 0.97 (0.68%)
Anti-infective monoclonal antibody (twice/year)	5000	2500	**Immediate:** 0.99 (1.11%)<br>**Long:** 0.97 (1.32%)	**Immediate:** 0.99 (0.91%)<br>**Long:** 0.99 (0.83%)
Anti-infective monoclonal antibody + Blood stage drug (once/year)	10000	5000	**Immediate:** 0.99 (1.34%)<br>**Long:** 0.96 (1.74%)	**Immediate:** 0.99 (1.18%)<br>**Long:** 0.98 (1.05%)
Anti-infective monoclonal antibody + Blood stage drug (twice/year)	5000	2500	**Immediate:** 0.99 (1.26%)<br>**Long:** 0.97 (1.98%)	**Immediate:** 0.99 (0.98%)<br>**Long:** 0.99 (1.12%)
Anti-infective vaccine (once/year)	10000	5000	**Immediate:** 0.99 (1.08%)<br>**Long:** 0.99 (1.3%)	**Immediate:** 0.99 (0.99%)<br>**Long:** 0.99 (1.16%)
Anti-infective vaccine + Blood stage drug (once/year)	5000	2500	**Immediate:** 0.99 (1.18%)<br>**Long:** 0.99 (1.63%)	**Immediate:** 0.99 (1.57%)<br>**Long:** 0.99 (2.25%)
Transmission-blocking vaccine (once/year)	10000	5000	**Immediate:** 0.99 (1.13)<br>**Long:** 0.99 (1.25%)	**Immediate:** 0.99 (0.89%)<br>**Long:** 0.99 (1.07%)
Transmission-blocking vaccine + Blood stage drug (once/year)	5000	2500	**Immediate:** 0.99 (1.25%)<br>**Long:** 0.99 (1.53%)	**Immediate:** 0.99 (1.68%)<br>**Long:** 0.99 (2.23 %)
Attractive targeted sugar baits (once/year)	5000	2500	**Immediate:** 0.99 (1.26%)<br>**Long:** 0.98 (1.71%)	**Immediate:** 0.99 (1.98%)<br>**Long:** 0.99 (1.19%)
Attractive targeted sugar baits (twice/year)	5000	2500	**Immediate:** 0.99 (1.09%)<br>**Long:** 0.99 (1.98%)	**Immediate:** 0.99 (1.03%)<br>**Long:** 0.99 (1.29%)
Eave tubes (once/year)	10000	5000	**Immediate:** 0.99 (1.11%)<br>**Long:** 0.99 (1.3%)	**Immediate:** 0.99 (0.89%)<br>**Long:** 0.99 (1.26%)

For each modelled transmission setting defined by case management level and mosquito biting patterns and for each intervention (Table S2.1), a comprehensive set of simulation scenarios was built by sampling uniformly the parameter space (defined in Table S2.1) and simulation with OpenMalaria. In this manner, a training and a test set were constructed. The training set was used to train, for each setting and intervention, a Heteroskedastic GP model in a 5-fold cross-validation procedure. The performance of the trained GP was assessed by computing the Pearson correlation coefficient r^2 as well as the mean error between the true and predicted outcomes on both out-of-sample cross-validation and test sets. For each intervention and follow-up (immediate or long-term), the average r^2 and mean error for all the GP models trained across 6 settings (seasonal or perennial, high, medium, or low mosquito indoor biting) are reported.

5 Finding minimal intervention properties and results for key determinants of impact

5.1 Finding minimal intervention properties

As summarized in the above and in the Methods, the trained GP models for each transmission setting and intervention were used within a general-purpose optimization scheme to identify minimum intervention properties that reach a defined *Pf*PR$_{0-99}$ reduction goal given operational and intervention constraints.

Let

$$g(\boldsymbol{x}) = g(x_1, x_2, x_3, x_4)$$

denote the GP model predicting the mean prevalence reduction obtained after deploying an intervention with given characteristics in a transmission setting, with

$$x_1 = tool\ coverage$$
$$x_2 = tool\ half-life$$
$$x_3 = tool\ efficacy$$
$$x_4 = access\ to\ treatment.$$

For various levels of *Pf*PR$_{0-99}$ denoted with p_k, each intervention characteristic was optimized separately, keeping the remaining characteristics as well as the level of case management fixed to pre-set levels. Precisely, the optimization procedure searches for

$$\min(x_i) |\ x_{\sim i}$$

such as

$$g(\boldsymbol{x}) \geq p_k$$

with the constraints:

$$l_i \leq x_i \leq u_i,$$

where l_i and u_i are the lower and upper bounds of x_i, respectively and the notation $\sim i$ is used to represent all the intervention characteristics except i. A detailed description of the parameter specifications during optimization for each intervention is provided in Additional file 1: Table S2.1.

To solve the above optimization problem, a general nonlinear augmented Lagrange multiplier method (50, 51) implemented in the R package *Rsolnp* (52) was used. To ensure optimality of the obtained solutions and to avoid local minima, 10 random restarts were chosen among 1,000 uniformly sampled input parameter sets and the optimization procedure was run separately for each restart (implemented in function *gosolnp* in the same R package). To capture the variance of the optimal intervention profile, since the output of a GP model is a distribution, the above optimization problem was solved for several cases, and the distribution of the obtained minima are reported when:

$$(i)\ \ g(\boldsymbol{x}) = \mu$$
$$(ii)\ g(\boldsymbol{x}) = \mu \pm \sigma$$
$$(iii)\ g(\boldsymbol{x}) = \mu \pm 2\sigma$$

where μ is the predicted mean of the GP model and σ is the standard deviation. Where the nonlinear optimization algorithm did not find any solutions, an additional fine grid search of 10,000 uniformly sampled data points was performed.

Under the simulated levels of case management, before intervention deployment, in seasonal settings, at low-transmission (simulated EIR < 2, corresponding simulated true *Pf*PR$_{2-10}$ < 11.7%), over 75% of simulations reached malaria elimination (*Pf*PR$_{0-99}$ = 0) (Additional file 1:

Fig. S3.5). For this reason, the space of obtained prevalence reductions following intervention deployment was rather sparse and the obtained optima were not reliable and often did not converge. Therefore, it was chosen to report minimum intervention profiles for settings with true *Pf*PR_{2-10} >= 11.7% (with RDTs this yields a patent *Pf*PR_{2-10} >= 5.8%).

5.2 Results: Key determinants of impact

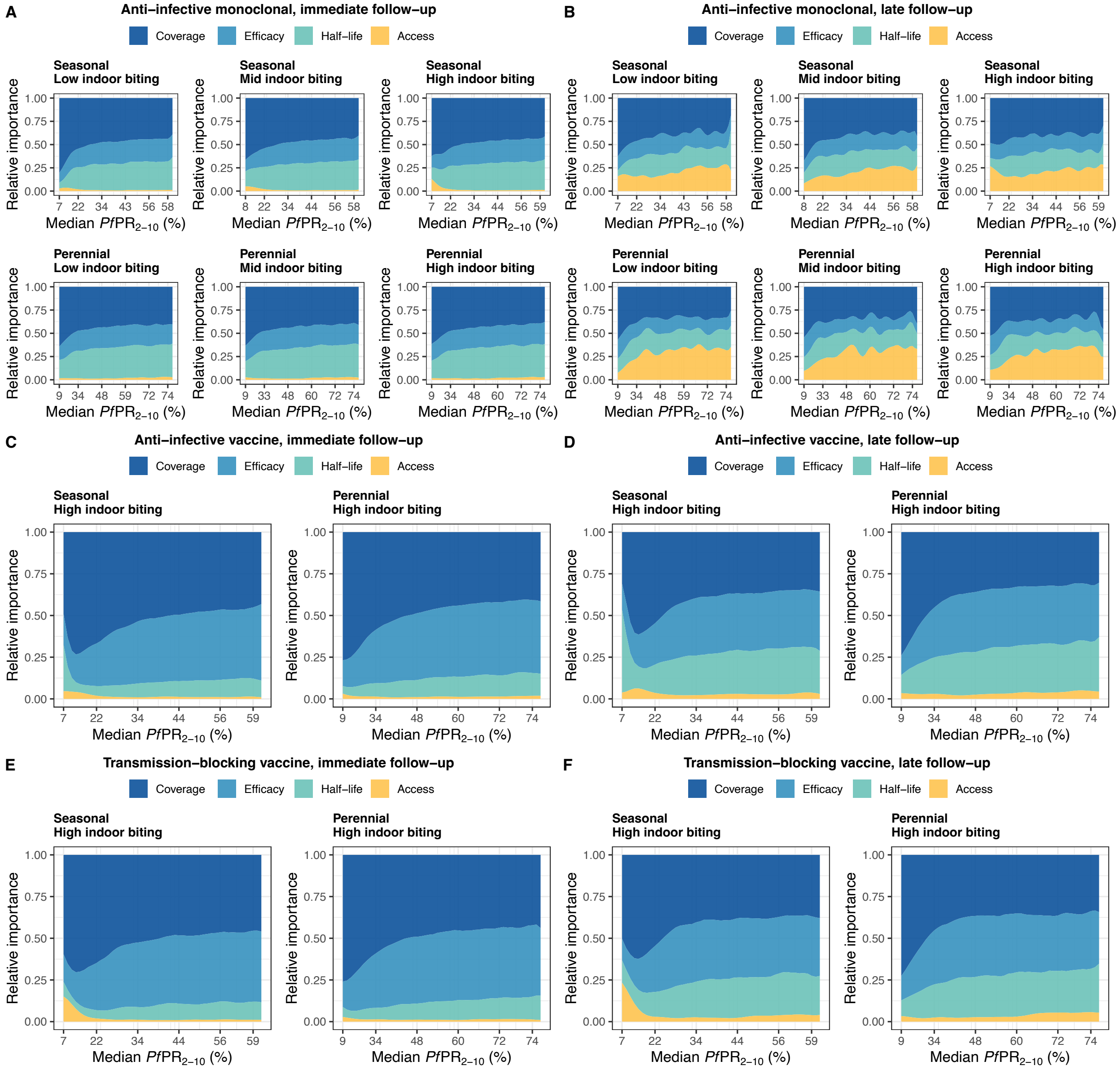

Fig. S5.1. Key drivers of impact for immunological malaria interventions across different transmission settings.

Results of sensitivity analysis identifying the determinants of intervention impact on *Pf*PR$_{0\text{-}99}$ reduction for anti-infective monoclonal antibodies (A, B), anti-infective vaccines (C, D) and transmission-blocking vaccines (E, F). The distinct colors represent proportions of the GP emulator output variance (relative importance) attributable to intervention efficacy, half-life, deployment coverage, as well as health system access. Determinants of impact are shown for both immediate and late follow-up when interventions are applied once per year for three years in different transmission settings (see full intervention specifications in the Additional file 1: section 1.2.3). The transmission settings are defined by two seasonal settings (seasonal and perennial) and three types of mosquito biting patterns (low, medium. and high indoor biting). The mosquito biting patterns had little to no effect on the results of the sensitivity analysis for

these immunological interventions (see results for all settings for monoclonal antibodies in figures A and B). Therefore, only the results for seasonal and perennial settings with high indoor mosquito biting are displayed for the vaccine interventions.

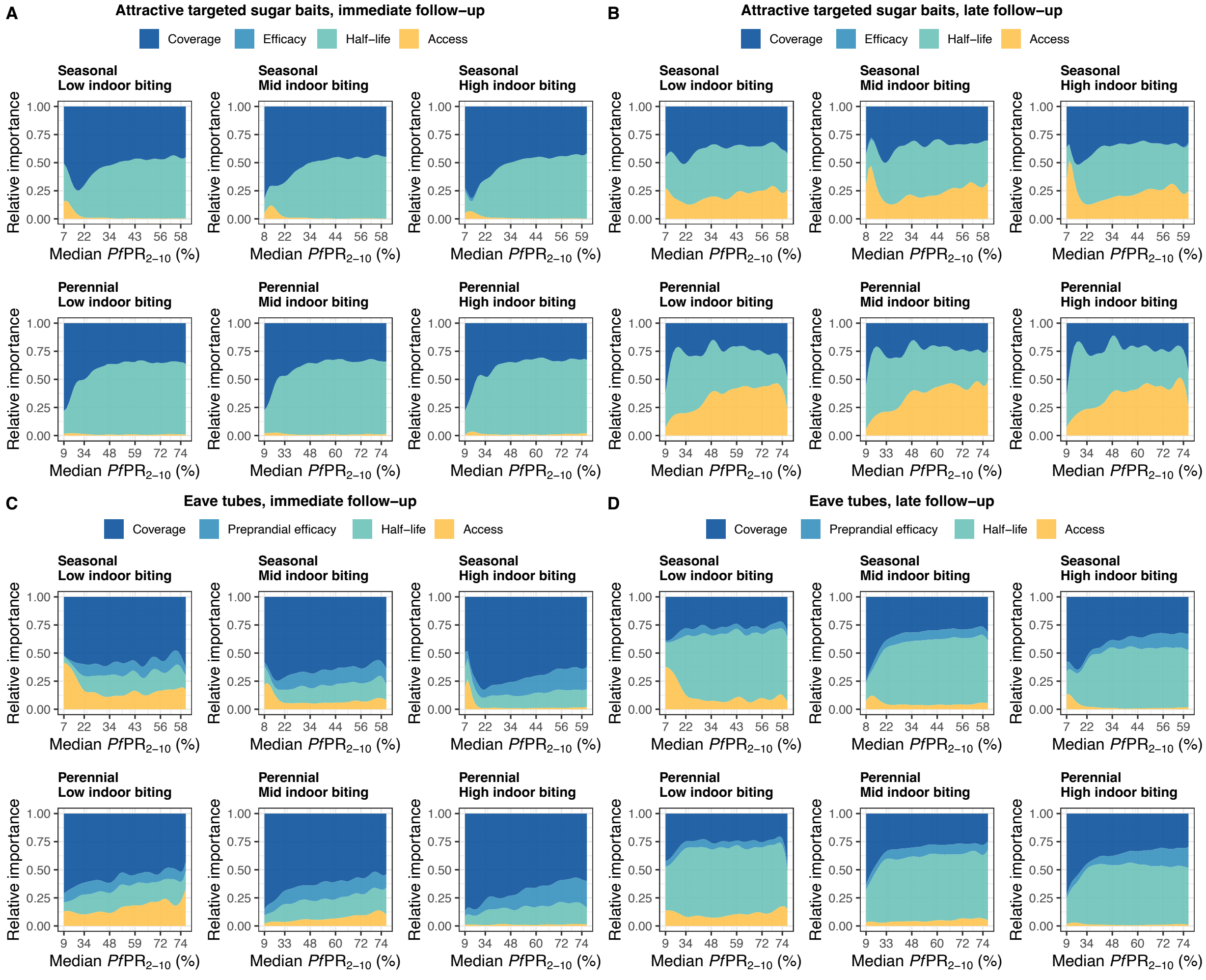

Fig. S5.2. Key drivers of impact for vector control malaria interventions across different transmission settings.

Results of sensitivity analysis identifying the determinants of intervention impact on PfPR$_{0-99}$ reduction for attractive targeted sugar baits (A, B) and eave tubes (C, D). The distinct colors represent proportions of the GP emulator output variance (relative importance) attributable to intervention efficacy, half-life, deployment coverage, as well as health system access. Determinants of impact are shown for both immediate and late follow-up when interventions are applied once per year for three years in different transmission settings (see full intervention specifications in the Additional file 1: section 1.2.3). The transmission settings are defined by two seasonal settings (seasonal and perennial) and three types of mosquito biting patterns (low, medium, and high indoor biting). Like for the immunological interventions in the previous figure, we see limited difference between key drivers for attractive targeted sugar baits in different biting settings as mosquitoes sugar feed before indoor or outdoor biting. In contrast, we observe that intervention properties of eave tubes rather than health system access to treatment

are larger drivers of impact in indoor biting settings, as mosquitoes in those settings will be more likely to contact the eave tube.

6 Results: Feasible landscapes of optimal, constrained intervention profiles

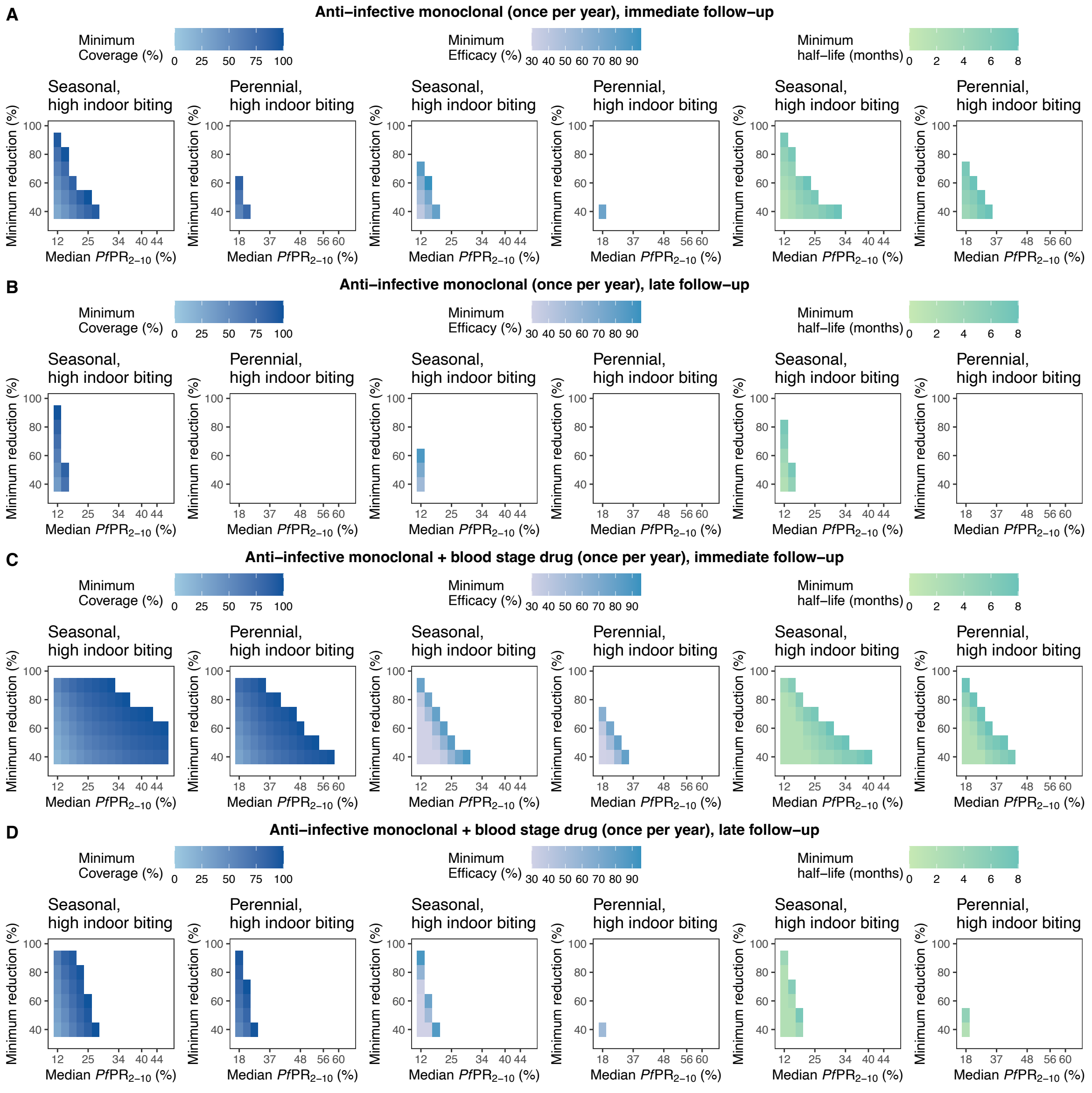

Fig. S6.1. Feasible landscapes of optimal, constrained intervention profiles (TPPs) for an anti-infective monoclonal antibody deployed once per year.

The heatmaps represent landscapes of optimal, constrained intervention characteristic profiles (minimum coverage, efficacy, and half-life) required to achieve various health goals (quantified by minimal reduction in $PfPR_{0-99}$, y-axis) across different simulated true $PfPR_{2-10}$ settings (rounded values, x-axis) with seasonal transmission and high indoor mosquito biting. Each intervention characteristic was minimized in turn, while keeping the other characteristics fixed

(fixed parameter values for each optimization are specified in Table S2.2). Results are shown for an anti-infective monoclonal antibody delivered alone and assessing immediate (A) and late (B) follow up, as well as when delivered in combination with a blood stage drug assessing immediate (C) and late (D) follow-up. The simulated case management level (E_5) for all the displayed optimization analyses was assumed 25%.

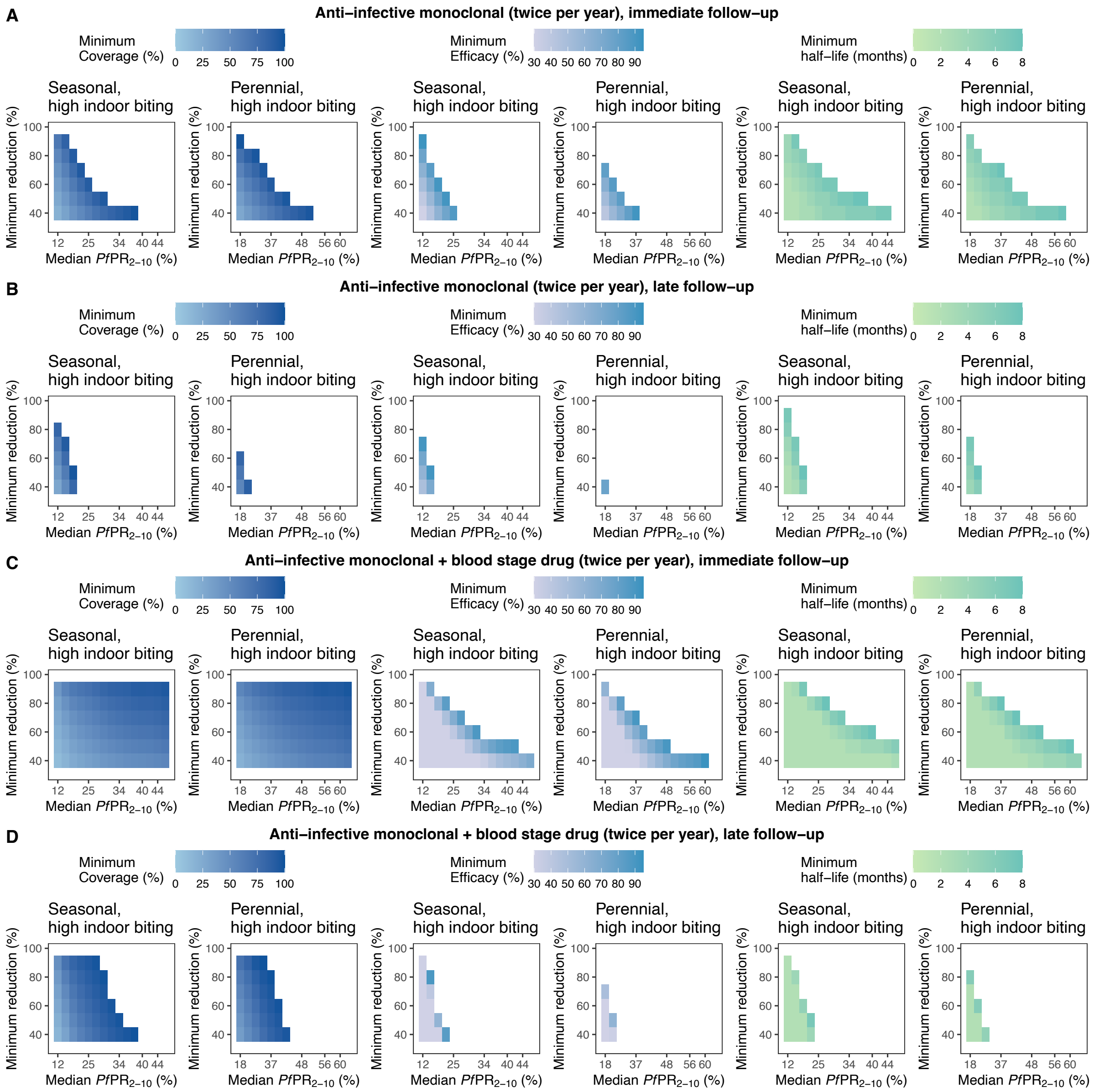

Fig. S6.2. Feasible landscapes of optimal, constrained intervention profiles (TPPs) for an anti-infective monoclonal antibody deployed twice per year.

The heatmaps represent landscapes of optimal, constrained intervention characteristic profiles (minimum coverage, efficacy, and half-life) required to achieve various health goals (quantified by minimal reduction in *Pf*PR$_{0-99}$, y-axis) across different simulated true *Pf*PR$_{2-10}$ settings (rounded values, x-axis) with seasonal transmission and high indoor mosquito biting. Each intervention characteristic was minimized in turn, while keeping the other characteristics fixed

(fixed parameter values for each optimization are specified in Table S2.2). Results are shown for an anti-infective monoclonal antibody delivered alone and assessing immediate (A) and late (B) follow up, as well as when delivered in combination with a blood stage drug assessing immediate (C) and late (D) follow-up. The simulated case management level (E_5) for all the displayed optimization analyses was assumed 25%.

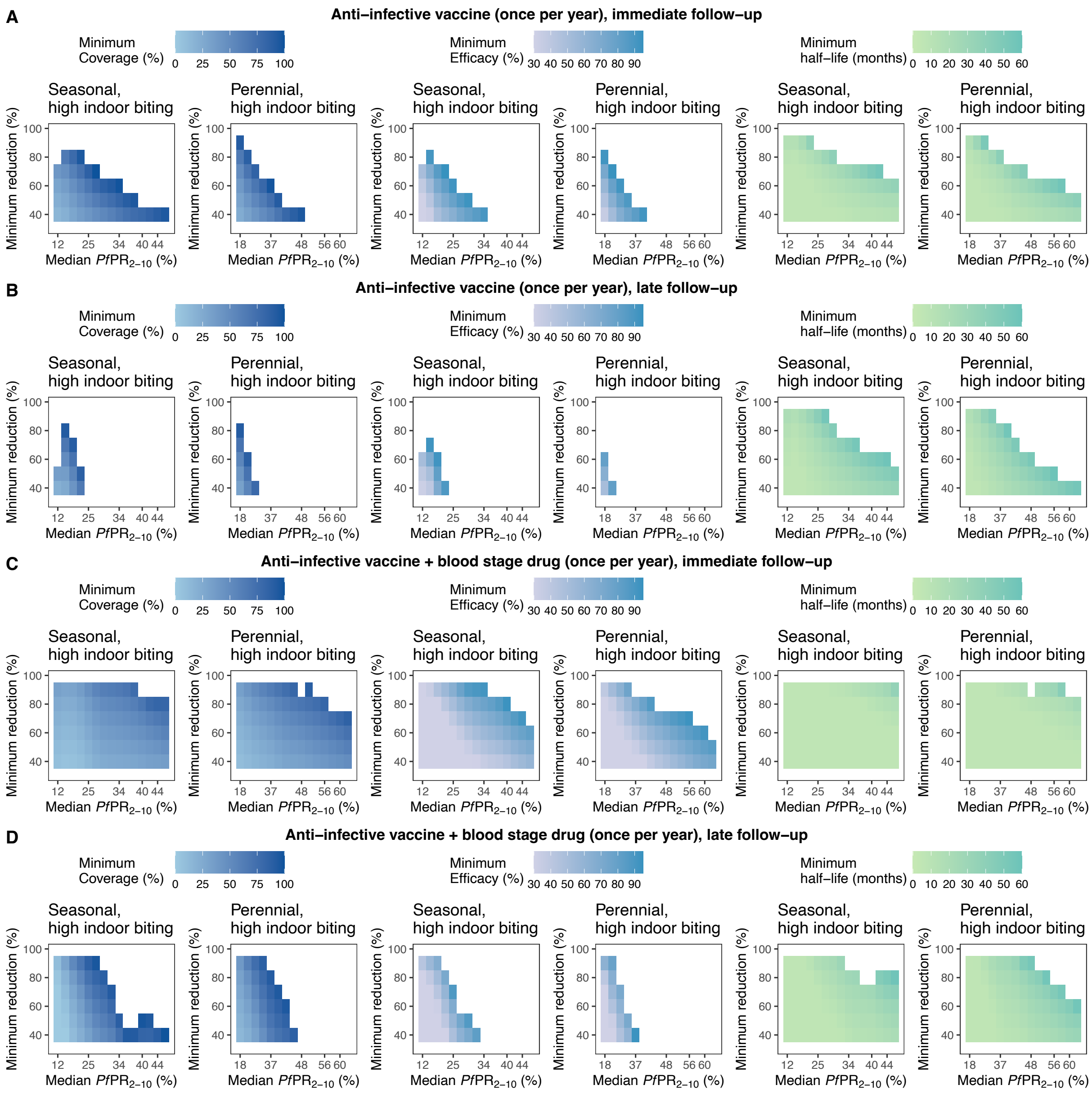

Fig. S6.3. Feasible landscapes of optimal, constrained intervention profiles (TPPs) for an anti-infective vaccine deployed once per year.

The heatmaps represent landscapes of optimal, constrained intervention characteristic profiles (minimum coverage, efficacy, and half-life) required to achieve various health goals (quantified by minimal reduction in *Pf*$PR_{0\text{-}99}$, y-axis) across different simulated true *Pf*$PR_{2\text{-}10}$ settings (rounded values, x-axis) with seasonal transmission and high indoor mosquito biting. Each intervention characteristic was minimized in turn, while keeping the other characteristics fixed

(fixed parameter values for each optimization are specified in Table S2.2). Results are shown for an anti-infective vaccine delivered alone and assessing immediate (A) and late (B) follow up, as well as when delivered in combination with a blood stage drug assessing immediate (C) and late (D) follow-up. The simulated case management level (E_5) for all the displayed optimization analyses was assumed 25%.

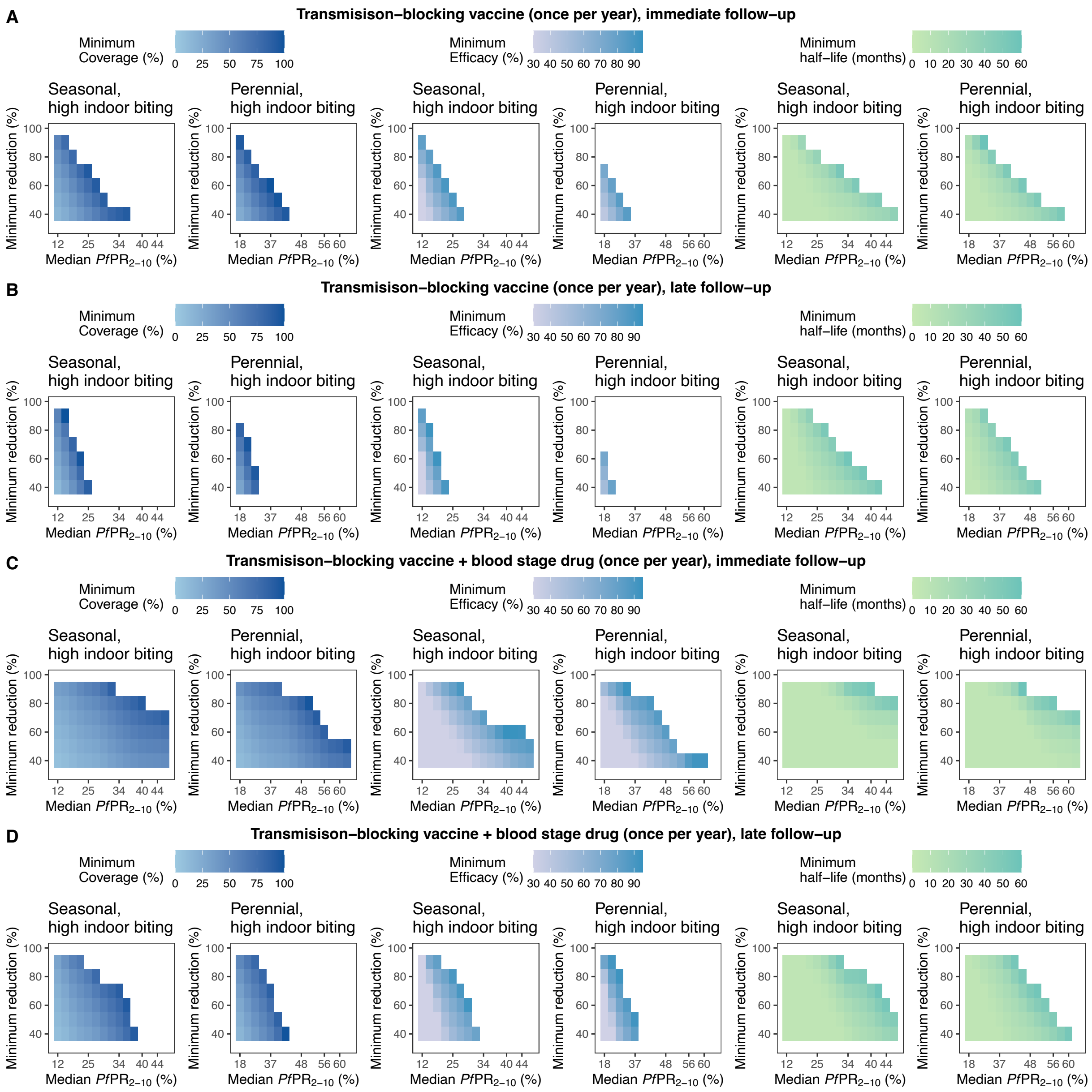

Fig. S6.4. Feasible landscapes of optimal, constrained intervention profiles (TPPs) for a transmission-blocking vaccine deployed once per year.

The heatmaps represent landscapes of optimal, constrained intervention characteristic profiles (minimum coverage, efficacy, and half-life) required to achieve various health goals (quantified by minimal reduction in *Pf*$PR_{0\text{-}99}$, y-axis) across different simulated true *Pf*$PR_{2\text{-}10}$ settings (rounded values, x-axis) with seasonal transmission and high indoor mosquito biting. Each

intervention characteristic was minimized in turn, while keeping the other characteristics fixed (fixed parameter values for each optimization are specified in Table S2.2). Results are shown for a transmission-blocking vaccine delivered alone and assessing immediate (A) and late (B) follow up, as well as when delivered in combination with a blood stage drug assessing immediate (C) and late (D) follow-up. The simulated case management level (E_5) for all the displayed optimization analyses was assumed 25%.

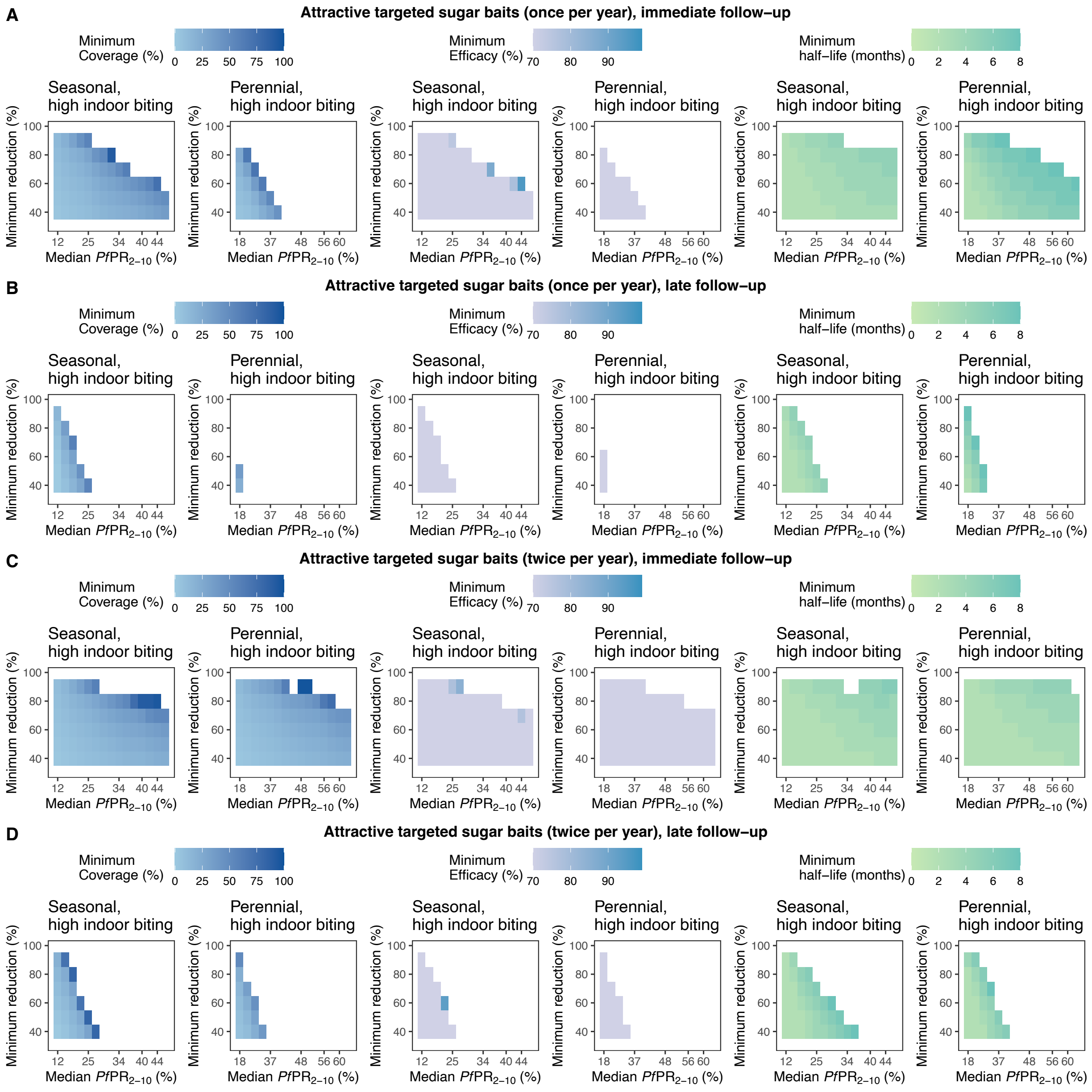

Fig. S6.5. Feasible landscapes of optimal, constrained intervention profiles (TPPs) for attractive targeted sugar baits deployed once or twice per year.

The heatmaps represent landscapes of optimal, constrained intervention characteristic profiles (minimum coverage, efficacy, and half-life) required to achieve various health goals (quantified by minimal reduction in *Pf*PR$_{0-99}$, y-axis) across different simulated true *Pf*PR$_{2-10}$ settings

(rounded values, x-axis) with seasonal transmission and high indoor mosquito biting. Each intervention characteristic was minimized in turn, while keeping the other characteristics fixed (fixed parameter values for each optimization are specified in Table S2.2). Results are shown for attractive targeted sugar baits delivered alone once per year and assessing immediate (A) and late (B) follow up, as well as when delivered twice per year assessing immediate (C) and late (D) follow-up. The simulated case management level (E_5) for all the displayed optimization analyses was assumed 25%.

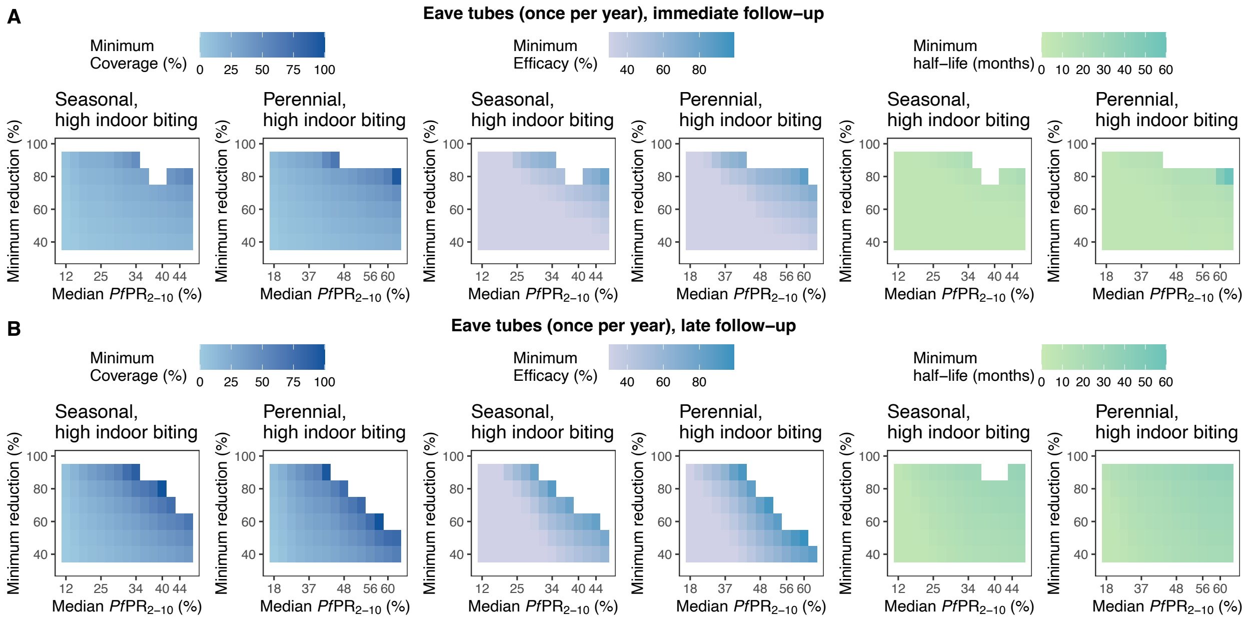

Fig. S6.6. Feasible landscapes of optimal, constrained intervention profiles (TPPs) for eave tubes deployed once per year.

Heatmaps represent landscapes of optimal, constrained intervention characteristic profiles (minimum coverage, efficacy, and half-life) required to achieve various health goals (quantified by minimal reduction in *Pf*PR$_{0\text{-}99}$, y-axis) across different simulated true *Pf*PR$_{2\text{-}10}$ settings (rounded values, x-axis) with seasonal or perennial transmission and high indoor mosquito biting (results for other biting patterns not shown as they are similar). Each intervention characteristic was minimized in turn, while keeping the other characteristics fixed (fixed parameter values for each optimization are specified in Table S2.2). Results are shown for eave tubes delivered alone and assessing immediate (A) and late (B) follow up. The simulated case management level (E_5) for all the displayed optimization analyses was assumed 25%.

7 Results: Optimal intervention profiles

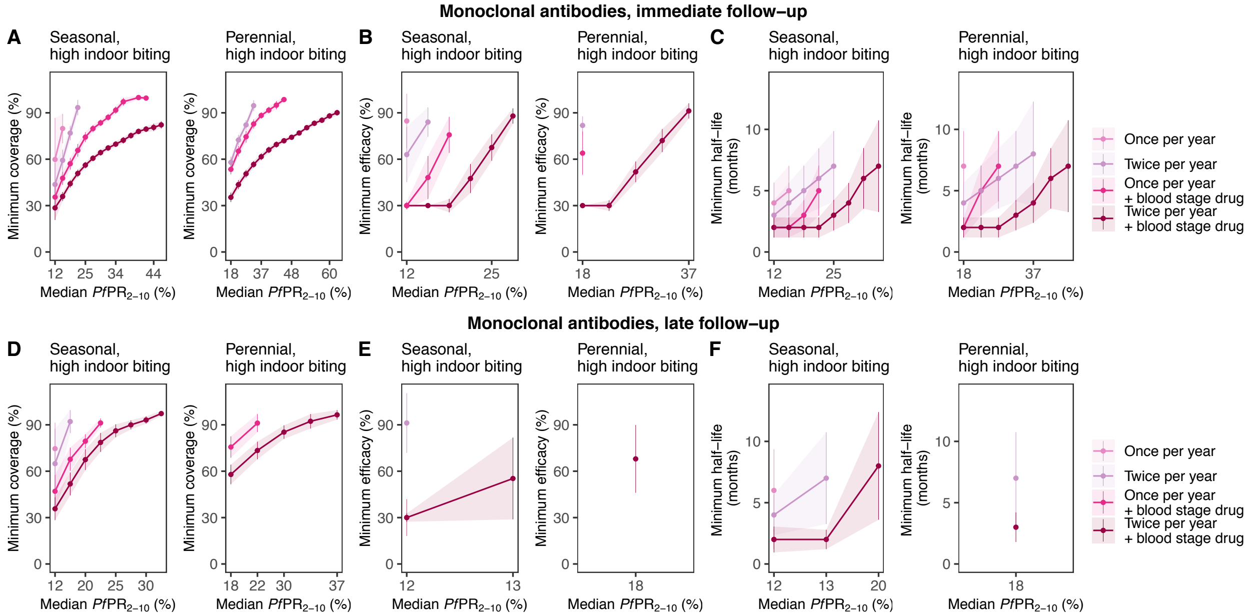

Fig. S7.1. Optimal intervention profiles (TPPs) for anti-infective monoclonal antibodies under various deployment regimes to achieve a PfPR0-99 reduction of at least 70%.

Each figure displays minimum, constrained intervention characteristic profiles (minimum coverage, efficacy, and half-life, y-axis) required to achieve a minimal reduction in *Pf*PR$_{0-99}$ of 70% across different simulated true *Pf*PR$_{2-10}$ settings (rounded values, x-axis) with seasonal transmission and high indoor mosquito biting. Each intervention characteristic was minimized in turn, while keeping the other characteristics fixed (fixed parameter values for each optimization are specified in Table S2.2). Results are shown when assessing *Pf*PR$_{0-99}$ reduction at immediate (A-C) and late (D-F) follow up. The simulated case management level (E$_5$) for all the displayed optimization analyses was assumed 25%.

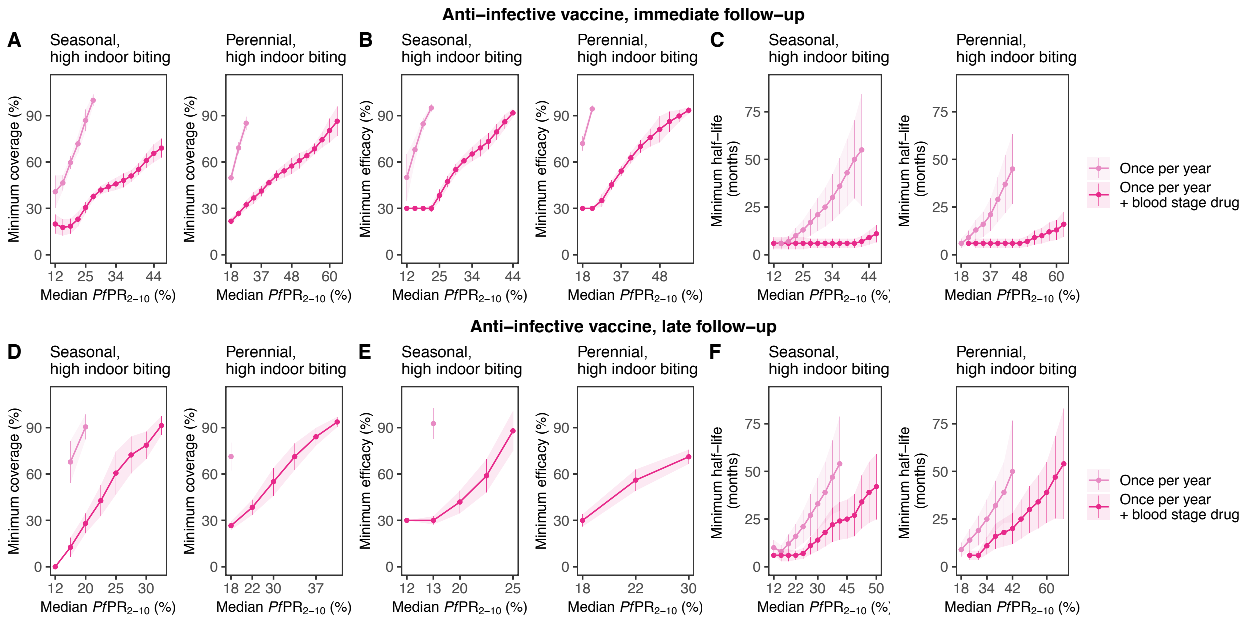

Fig. S7.2. Optimal intervention profiles (TPPs) for anti-infective vaccines under various deployment regimes to achieve a PfPR0-99 reduction of at least 70%.

Each figure displays minimum, constrained intervention characteristic profiles (minimum coverage, efficacy, and half-life, y-axis) required to achieve a minimal reduction in *Pf*PR$_{0-99}$ of 70% across different simulated true *Pf*PR$_{2-10}$ settings (rounded values, x-axis) with seasonal transmission and high indoor mosquito biting. Each intervention characteristic was minimized in turn, while keeping the other characteristics fixed (fixed parameter values for each optimization are specified in Table S2.2). Results are shown when assessing *Pf*PR$_{0-99}$ reduction at immediate (A-C) and late (D-F) follow up. The simulated case management level (E_5) for all the displayed optimization analyses was assumed 25%.

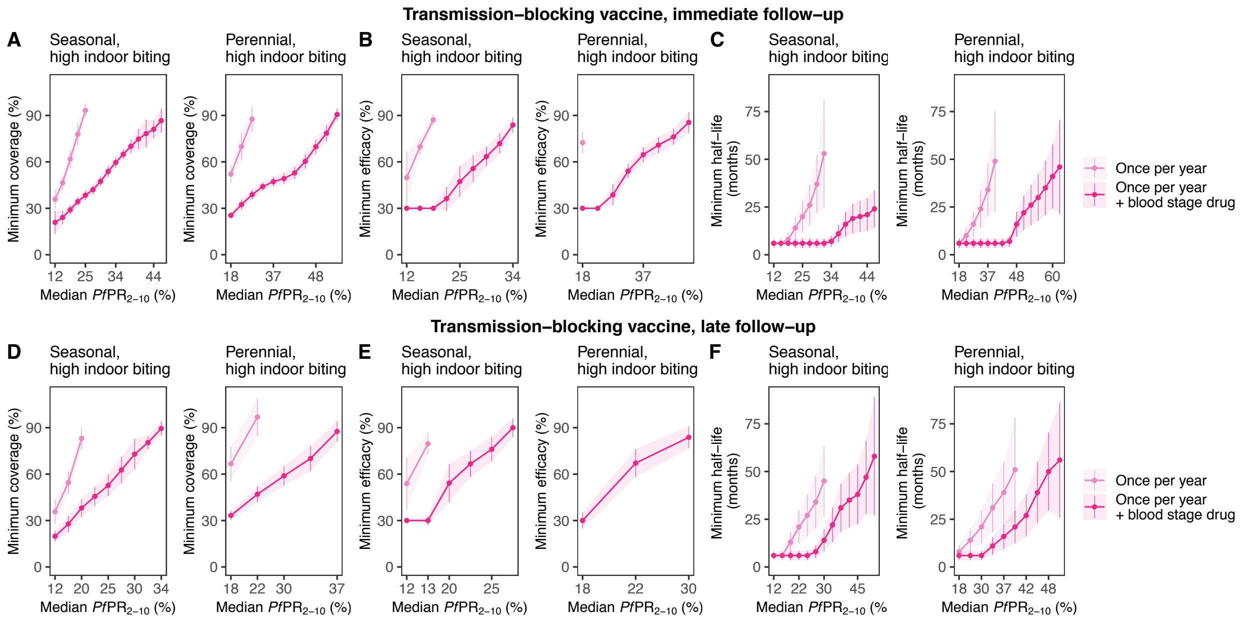

Fig. S7.3. Optimal intervention profiles (TPPs) for transmission-blocking vaccines under various deployment regimes to achieve a *Pf*PR$_{0\text{-}99}$ reduction of at least 70%.

Each figure displays minimum, constrained intervention characteristic profiles (minimum coverage, efficacy, and half-life, y-axis) required to achieve a minimal reduction in *Pf*PR$_{0\text{-}99}$ of 70% across different simulated true *Pf*PR$_{2\text{-}10}$ settings (rounded values, x-axis) with seasonal transmission and high indoor mosquito biting. Each intervention characteristic was minimized in turn, while keeping the other characteristics fixed (fixed parameter values for each optimization are specified in Table S2.2). Results are shown when assessing *Pf*PR$_{0\text{-}99}$ reduction at immediate (A-C) and late (D-F) follow up. The simulated case management level (E_5) for all the displayed optimization analyses was assumed 25%.

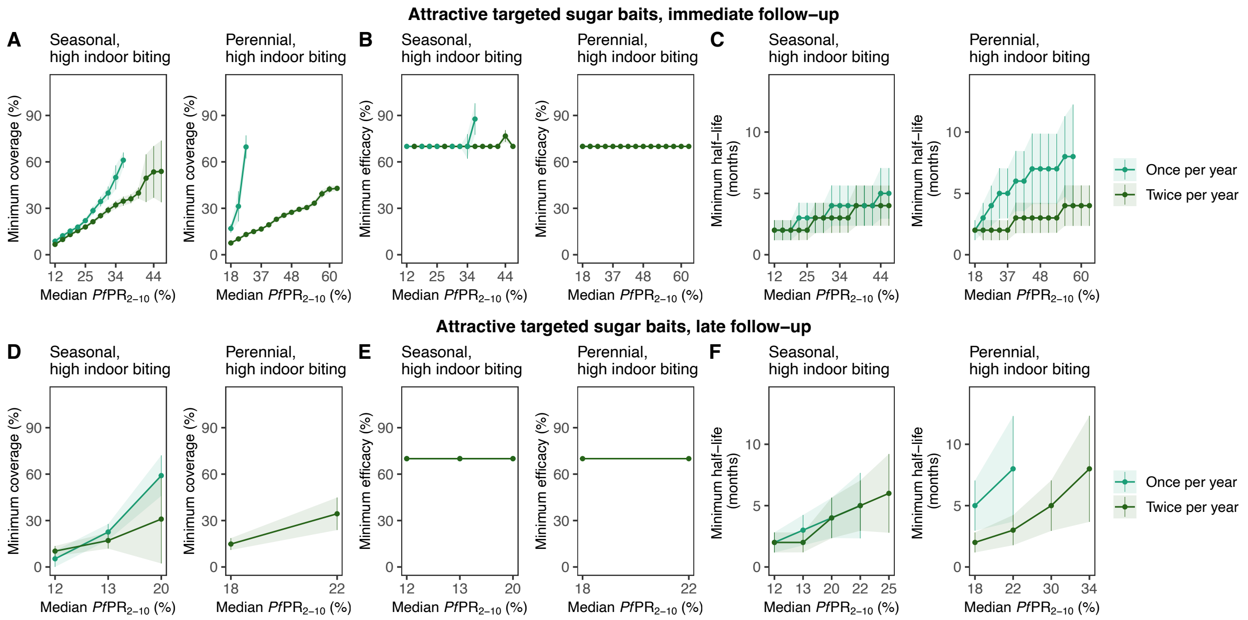

Fig. S7.4. Optimal intervention profiles (TPPs) for attractive targeted sugar baits under various deployment regimes to achieve a PfPR0-99 reduction of at least 70%.

Each figure displays minimum, constrained intervention characteristic profiles (minimum coverage, efficacy, and half-life, y-axis) required to achieve a minimal reduction in *Pf*PR$_{0\text{-}99}$ of 70% across different simulated true *Pf*PR$_{2\text{-}10}$ settings (rounded values, x-axis) with seasonal transmission and high indoor mosquito biting. Each intervention characteristic was minimized in turn, while keeping the other characteristics fixed (fixed parameter values for each optimization are specified in Table S2.2). Results are shown when assessing *Pf*PR$_{0\text{-}99}$ reduction at immediate (A-C) and late (D-F) follow up. The simulated case management level (E_5) for all the displayed optimization analyses was assumed 25%.

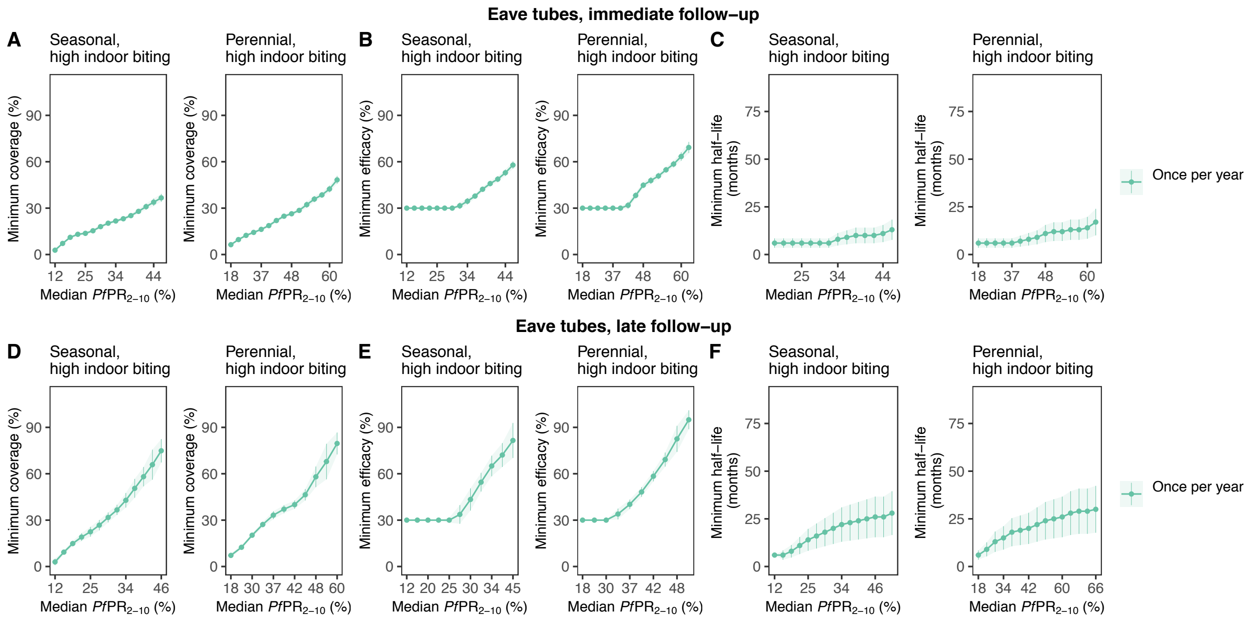

Fig. S7.5. Optimal intervention profiles (TPPs) for eave tubes to achieve a PfPR0-99 reduction of at least 70%.

Each figure displays minimum, constrained intervention characteristic profiles (minimum coverage, efficacy, and half-life, y-axis) required to achieve a minimal reduction in PfPR0-99 of 70% across different simulated true PfPR2-10 settings (rounded values, x-axis) with seasonal transmission and high indoor mosquito biting. Each intervention characteristic was minimized in turn, while keeping the other characteristics fixed (fixed parameter values for each optimization are specified in Table S2.2). Results are shown when assessing PfPR0-99 reduction at immediate (A-C) and late (D-F) follow up. The simulated case management level (E5) for all the displayed optimization analyses was assumed 25%.

References

1. Brooks A, Nunes JK, Garnett A, Biellik R, Leboulleux D, Birkett AJ, et al. Aligning new interventions with developing country health systems: Target product profiles, presentation, and clinical trial design. Global Public Health. 2012;7(9):931-45.
2. Chin R, Lee BY. Principles and practice of clinical trial medicine: Elsevier; 2008.
3. Burrows JN, Duparc S, Gutteridge WE, van Huijsduijnen RH, Kaszubska W, Macintyre F, et al. New developments in anti-malarial target candidate and product profiles. Malar J. 2017;16(1):26.
4. Cocco P, Ayaz-Shah A, Messenger MP, West RM, Shinkins B. Target Product Profiles for medical tests: a systematic review of current methods. BMC medicine. 2020;18:1-12.
5. Abeku TA. Response to malaria epidemics in Africa. Emerg Infect Dis. 2007;13:681-6.
6. Lee BY, Burke DS. Constructing target product profiles (TPPs) to help vaccines overcome post-approval obstacles. Vaccine. 2010;28(16):2806-9.
7. Feachem RG, Chen I, Akbari O, Bertozzi-Villa A, Bhatt S, Binka F, et al. Malaria eradication within a generation: ambitious, achievable, and necessary. The Lancet. 2019;394(10203):1056-112.
8. Chitnis N, Smith T, Steketee R. A mathematical model for the dynamics of malaria in mosquitoes feeding on a heterogeneous host population. J Biol Dyn. 2008;2(3):259-85.
9. Chitnis N, Hardy D, Smith T. A periodically-forced mathematical model for the seasonal dynamics of malaria in mosquitoes. Bull Math Biol. 2012;74(5):1098-124.
10. Maire N, Smith T, Ross A, Owusu-Agyei S, Dietz K, Molineaux L. A model for natural immunity to asexual blood stages of Plasmodium falciparum malaria in endemic areas. The American journal of tropical medicine and hygiene. 2006;75(2_suppl):19-31.
11. Ross A, Killeen G, Smith T. Relationships between host infectivity to mosquitoes and asexual parasite density in Plasmodium falciparum. The American journal of tropical medicine and hygiene. 2006;75(2_suppl):32-7.
12. Ross A, Maire N, Molineaux L, Smith T. An epidemiologic model of severe morbidity and mortality caused by Plasmodium falciparum. The American journal of tropical medicine and hygiene. 2006;75(2_suppl):63-73.
13. Smith T, Ross A, Maire N, Rogier C, Trape J-F, Molineaux L. An epidemiologic model of the incidence of acute illness in Plasmodium falciparum malaria. The American journal of tropical medicine and hygiene. 2006;75(2_suppl):56-62.
14. Tediosi F, Maire N, Smith T, Hutton G, Utzinger J, Ross A, et al. An approach to model the costs and effects of case management of plasmodium falciparum malaria in sub-saharan Africa. The American Journal of Tropical Medicine and Hygiene. 2006;75(2_suppl):90-103.
15. Smith T, Killeen GF, Maire N, Ross A, Molineaux L, Tediosi F, et al. Mathematical modeling of the impact of malaria vaccines on the clinical epidemiology and natural history of Plasmodium falciparum malaria: overview. The American Journal of Tropical Medicine and Hygiene. 2006;75(2_suppl):1-10.
16. Reiker T, Golumbeanu M, Shattock A, Burgert L, Smith TA, Filippi S, et al. Machine learning approaches to calibrate individual-based infectious disease models. medRxiv. 2021.
17. Penny MA, Verity R, Bever CA, Sauboin C, Galactionova K, Flasche S, et al. Public health impact and cost-effectiveness of the RTS, S/AS01 malaria vaccine: a systematic comparison of predictions from four mathematical models. The Lancet. 2016;387(10016):367-75.

18. Brady OJ, Slater HC, Pemberton-Ross P, Wenger E, Maude RJ, Ghani AC, et al. Role of mass drug administration in elimination of Plasmodium falciparum malaria: a consensus modelling study. The Lancet Global Health. 2017;5(7):e680-e7.
19. Gatton ML, Chitnis N, Churcher T, Donnelly MJ, Ghani AC, Godfray HCJ, et al. The importance of mosquito behavioural adaptations to malaria control in Africa. Evolution: international journal of organic evolution. 2013;67(4):1218-30.
20. Stuckey EM, Smith T, Chitnis N. Seasonally Dependent Relationships between Indicators of Malaria Transmission and Disease Provided by Mathematical Model Simulations. PLOS Computational Biology. 2014;10(9):e1003812.
21. Penny MA, Maire N, Bever CA, Pemberton-Ross P, Briët OJT, Smith DL, et al. Distribution of malaria exposure in endemic countries in Africa considering country levels of effective treatment. Malaria Journal. 2015;14(1):384.
22. Smith NR, Trauer JM, Gambhir M, Richards JS, Maude RJ, Keith JM, et al. Agent-based models of malaria transmission: a systematic review. Malaria journal. 2018;17(1):1-16.
23. Smith T, Maire N, Ross A, Penny M, Chitnis N, Schapira A, et al. Towards a comprehensive simulation model of malaria epidemiology and control. Parasitology. 2008;135(13):1507-16.
24. Reiker T, Chitnis N, Smith TA. Modelling reactive case detection strategies for interrupting transmission of Plasmodium falciparum malaria. Malaria Journal. 2019;18(1):259.
25. Smith T, Ross A, Maire N, Chitnis N, Studer A, Hardy D, et al. Ensemble Modeling of the Likely Public Health Impact of a Pre-Erythrocytic Malaria Vaccine. PLOS Medicine. 2012;9(1):e1001157.
26. Smith T, Maire N, Dietz K, Killeen GF, Vounatsou P, Molineaux L, et al. Relationship between the entomologica inoculation rate and the force of infection for Plasmodium Falciparum malaria. The American Journal of Tropical Medicine and Hygiene. 2006;75(2_suppl):11-8.
27. Collins WE, Jeffery GM. A retrospective examination of the patterns of recrudescence in patients infected with Plasmodium falciparum. The American journal of tropical medicine and hygiene. 1999;61(1_Supplement):44-8.
28. Killeen GF, Ross A, Smith T. Infectiousness of malaria-endemic human populations to vectors. The American journal of tropical medicine and hygiene. 2006;75(2_suppl):38-45.
29. Ross A, Smith T. The effect of malaria transmission intensity on neonatal mortality in endemic areas. The American journal of tropical medicine and hygiene. 2006;75(2_suppl):74-81.
30. Ekström AM, Clark J, Byass P, Lopez A, De Savigny D, Moyer CA, et al. INDEPTH Network: contributing to the data revolution. The Lancet Diabetes & Endocrinology. 2016;4(2):97.
31. Korenromp E, Mahiané G, Hamilton M, Pretorius C, Cibulskis R, Lauer J, et al. Malaria intervention scale-up in Africa: effectiveness predictions for health programme planning tools, based on dynamic transmission modelling. Malaria Journal. 2016;15(1):417.
32. Yukich JO, Chitnis N. Modelling the implications of stopping vector control for malaria control and elimination. Malaria journal. 2017;16(1):1-16.
33. Camponovo F, Ockenhouse CF, Lee C, Penny MA. Mass campaigns combining antimalarial drugs and anti-infective vaccines as seasonal interventions for malaria control, elimination and prevention of resurgence: a modelling study. BMC Infect Dis. 2019;19(1):920.
34. Maire N, Tediosi F, Ross A, Smith T. Predictions of the epidemiologic impact of introducing a pre-erythrocytic vaccine into the expanded program on immunization in sub-

Saharan Africa. The American Journal of Tropical Medicine and Hygiene. 2006;75(2_suppl):111-8.
35. Kileen GF, Ross A, Smith T. Infectiousness of malaria-endemic human populations to vectors. The American Journal of Tropical Medicine and Hygiene. 2006;75(2_suppl):38-45.
36. Smith TC, Charlwood JDD, Kihonda J, Mwankusye S, Billingsley PF, Meuwissen J, et al. Absence of seasonal variation in malaria parasitaemia in an area of intense seasonal transmission. Acta tropica. 1993;54 1:55-72.
37. Drakeley C, Schellenberg D, Kihonda J, Sousa C, Arez A, Lopes D, et al. An estimation of the entomological inoculation rate for Ifakara: a semi-urban area in a region of intense malaria transmission in Tanzania. Tropical Medicine & International Health. 2003;8(9):767-74.
38. Shaukat AM, Breman JG, McKenzie FE. Using the entomological inoculation rate to assess the impact of vector control on malaria parasite transmission and elimination. Malaria journal. 2010;9(1):122.
39. Tusting LS, Bousema T, Smith DL, Drakeley C. Measuring changes in Plasmodium falciparum transmission: precision, accuracy and costs of metrics. Advances in parasitology. 84: Elsevier; 2014. p. 151-208.
40. Rasmussen CE, Williams CKI. Gaussian Processes for Machine Learning (Adaptive Computation and Machine Learning): The MIT Press; 2005.
41. Stein M. Large Sample Properties of Simulations Using Latin Hypercube Sampling. Technometrics. 1987;29(2):143-51.
42. Binois M, Gramacy RB, Ludkovski M. Practical heteroscedastic gaussian process modeling for large simulation experiments. Journal of Computational and Graphical Statistics. 2018;27(4):808-21.
43. Binois M, Gramacy R. hetGP: Heteroskedastic Gaussian Process Modeling and Design under Replication. R package version. 2017;1(0).
44. Gopakumar AM, Balachandran PV, Xue D, Gubernatis JE, Lookman T. Multi-objective optimization for materials discovery via adaptive design. Scientific reports. 2018;8(1):3738.
45. Dehghannasiri R, Xue D, Balachandran PV, Yousefi MR, Dalton LA, Lookman T, et al. Optimal experimental design for materials discovery. Computational Materials Science. 2017;129:311-22.
46. Binois M, Huang J, Gramacy RB, Ludkovski M. Replication or Exploration? Sequential Design for Stochastic Simulation Experiments. Technometrics. 2019;61(1):7-23.
47. Sobol′ IM. Global sensitivity indices for nonlinear mathematical models and their Monte Carlo estimates. Mathematics and Computers in Simulation. 2001;55(1):271-80.
48. Saltelli A, Tarantola S, Campolongo F, Ratto M. Sensitivity Analysis in Practice: A Guide to Assessing Scientific Models: Halsted Press; 2004.
49. Team RC. R: A language and environment for statistical computing. Vienna, Austria; 2013.
50. Hestenes MR. Multiplier and gradient methods. Journal of Optimization Theory and Applications. 1969;4(5):303-20.
51. Ye Y. Interior algorithms for linear, quadratic, and linearly constrained non-linear programming: Ph. D. thesis, Department of ESS, Stanford University; 1987.
52. Ghalanos A, Theussl S. Rsolnp: general non-linear optimization using augmented Lagrange multiplier method. R package version. 2012;1.
